# DNA Replication across α-l-(3′-2′)-Threofuranosyl Nucleotides Mediated by Human DNA Polymerase η

**DOI:** 10.1021/acs.biochem.4c00387

**Published:** 2024-09-11

**Authors:** Rachana Tomar, Pratibha P. Ghodke, Amritraj Patra, Elizabeth Smyth, Alexander Pontarelli, William Copp, F. Peter Guengerich, John C. Chaput, Christopher J. Wilds, Michael P. Stone, Martin Egli

**Affiliations:** † Department of Chemistry, Vanderbilt Ingram Cancer Center, and Vanderbilt Center for Structural Biology, Vanderbilt University, Nashville, Tennessee 37235, United States; ‡ Department of Biochemistry, School of Medicine, Vanderbilt Ingram Cancer Center, and Vanderbilt Center for Structural Biology, Vanderbilt University, Nashville, Tennessee 37232, United States; § Department of Chemistry and Biochemistry, Concordia University, Montréal, Québec H4B 1R6, Canada; ∥ Department of Pharmaceutical Sciences, University of California, Irvine, California 92697, United States

## Abstract

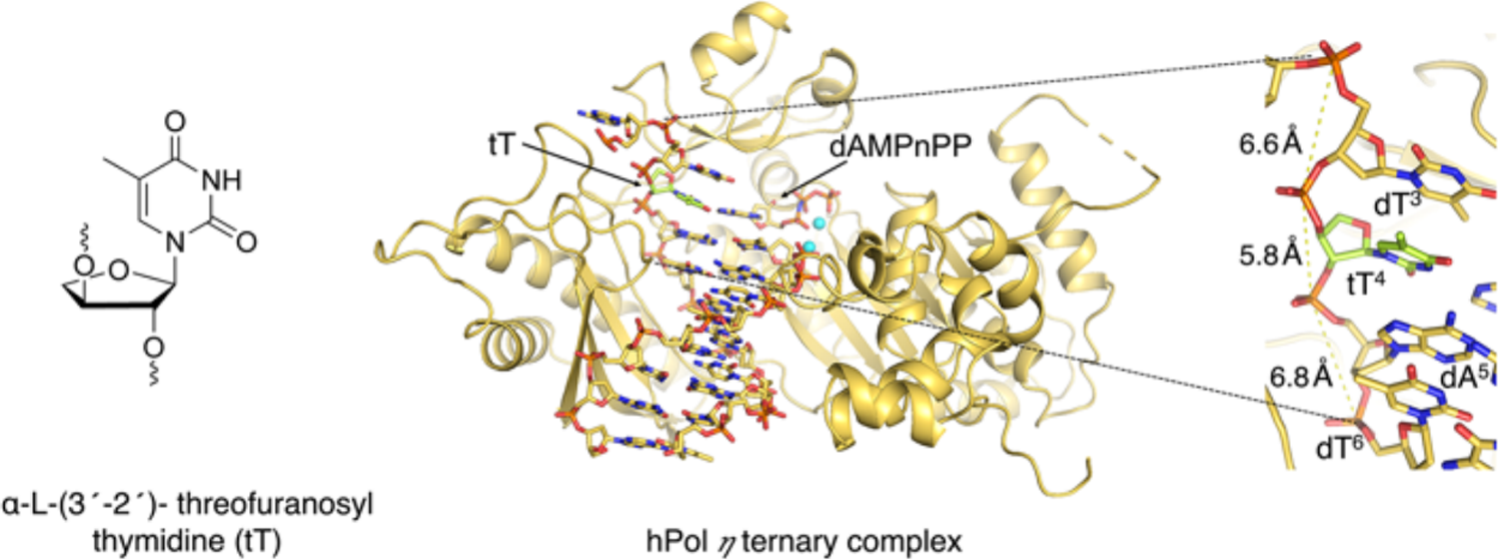

α-l-(3′-2′)-Threofuranosyl nucleic acid (TNA) pairs with itself, cross-pairs with DNA and RNA, and shows promise as a tool in synthetic genetics, diagnostics, and oligonucleotide therapeutics. We studied *in vitro* primer insertion and extension reactions catalyzed by human trans-lesion synthesis (TLS) DNA polymerase η (hPol η) opposite a TNA-modified template strand without and in combination with *O*^4^-alkyl thymine lesions. Across TNA-T (tT), hPol η inserted mostly dAMP and dGMP, dTMP and dCMP with lower efficiencies, followed by extension of the primer to a full-length product. hPol η inserted dAMP opposite *O*^4^-methyl and -ethyl analogs of tT, albeit with reduced efficiencies relative to tT. Crystal structures of ternary hPol η complexes with template tT and *O*^4^-methyl tT at the insertion and extension stages demonstrated that the shorter backbone and different connectivity of TNA compared to DNA (3′ → 2′ versus 5′ → 3′, respectively) result in local differences in sugar orientations, adjacent phosphate spacings, and directions of glycosidic bonds. The 3′-OH of the primer’s terminal thymine was positioned at 3.4 Å on average from the α-phosphate of the incoming dNTP, consistent with insertion opposite and extension past the TNA residue by hPol η. Conversely, the crystal structure of a ternary hPol η·DNA·tTTP complex revealed that the primer’s terminal 3′-OH was too distant from the tTTP α-phosphate, consistent with the inability of the polymerase to incorporate TNA. Overall, our study provides a better understanding of the tolerance of a TLS DNA polymerase vis-à-vis unnatural nucleotides in the template and as the incoming nucleoside triphosphate.

## Introduction

Why pentose and not hexose, and if pentose, why ribofuranosyl nucleic acid as opposed to another choice of an aldose sugar-linked phosphate backbone? These questions, raised by Eschenmoser and colleagues, marked the beginning of a systematic study into the chemical etiology of nucleic acid structure.^1−4^ To gain insight into nature’s choice of the extant nucleic acids, phosphodiester-based oligonucleotides featuring alternative sugars in place of the natural ribose or 2′-deoxyribose sugar were synthesized and systematically evaluated for the ability to self-pair and cross-pair with RNA and DNA.^1−8^ Among the synthetic genetic polymers evaluated, α-l-threofuranosyl nucleic acid (TNA) with a backbone of tetrose sugars connected by a 3′ → 2′ phosphodiester linkage showed a remarkable ability to form stable Watson–Crick duplexes with DNA, RNA, and TNA.^1,2^ TNA’s more closely spaced phosphates relative to the natural backbone despite a pseudotransdiaxial orientation of 3′- and 2′-phosphate groups on the five-membered tetrose ring confer excellent nuclease resistance^9^ and superior biological stability.^2,10^ Abiogenesis of pyrimidine threonucleotides from glycolaldehyde and cyanamide in the presence of phosphate raises the intriguing possibility of TNA as prebiotic alternative to RNA.^1−4,11,12^ Its simplicity and ability to cross-pair with both DNA and RNA render TNA a promising molecular tool for antisense, RNAi, and aptamer applications as well as catalytic threozymes.^13−20^

Structural studies provided insights into the pairing properties of TNA and hybrids between TNA and DNA or RNA.^13^ The atomic-resolution X-ray crystal structure of a B-form DNA duplex [d(CGCGAA)tTd(TCGCG)]_2_^21^ with a TNA-T (tT) showed that the four-carbon threose sugar is easily accommodated within an otherwise natural DNA duplex and stacking interactions are retained.^22^ TNA/RNA heteroduplexes were thermodynamically more stable than TNA/DNA duplexes because TNA adopts an A-form-like helical geometry and DNA is unable to fully adapt to the conformational constraints of the more rigid TNA backbone with uniformly C4′-*exo* puckered tetroses.^23,24^ Moreover, the thermal stability of TNA/DNA heteroduplexes showed a strong purine dependence, with heteroduplexes of higher TNA purine contents displaying increased melting temperatures.^25^ The NMR solution structure of the TNA octamer t(CGAATTCG) confirmed the formation of a right-handed, antiparallel double helix with Watson–Crick base-pairing.^26^

For TNA to be considered a simpler precursor to RNA, in addition to a plausible prebiotic synthetic path and the ability to cross-pair with RNA, TNA also has to fold into three-dimensional structures that enable ligand binding and catalytic properties.^13^ Thus, TNA or chimeric TNA-RNA oligonucleotides were shown to act as templates to facilitate the nonenzymatic oligomerization of RNA, thereby mimicking the process of genetic takeover of TNA by RNA.^27,28^ Moreover, an RNA-dependent RNA polymerase (pol) ribozyme was shown to catalyze the polymerization of RNA monomer building blocks on a TNA template.^29^ As TNA-dependent TNA pol activity has remained unattainable so far, multiple efforts were made to engineer DNA-dependent TNA pols and TNA-dependent DNA pols, including Therminator, KOD-RSGA, SuperScript II, Kod RI, and Bst DNA pol.^30−35^ Few DNA polymerases such as Therminator, Deep Vent (exo-), HIV Reverse transcriptase, and Bst pol I are known to utilize TNA-based nucleotide triphosphates for TNA synthesis.^30,31,36−39^ These allow for efficient and faithful transfer of genetic information back and forth between TNA and DNA and, hence, enable *in vitro* selection of functional TNA molecules.^13,36−38^ The limited number of studies with TNA-compatible engineered pols or natural DNA pols regarding their ability for information transfer from one type of nucleic acid to another^40^ motivated us to explore the behavior of the Y-family human DNA pol η (hPol η) vis-à-vis TNA. hPol η is known to tolerate a wide variety of DNA adducts/lesions.^41−45^ Further, we are also interested in evaluating the propensity of hPol η to handle adducted nucleobases with a TNA backbone.

**Scheme 1:**
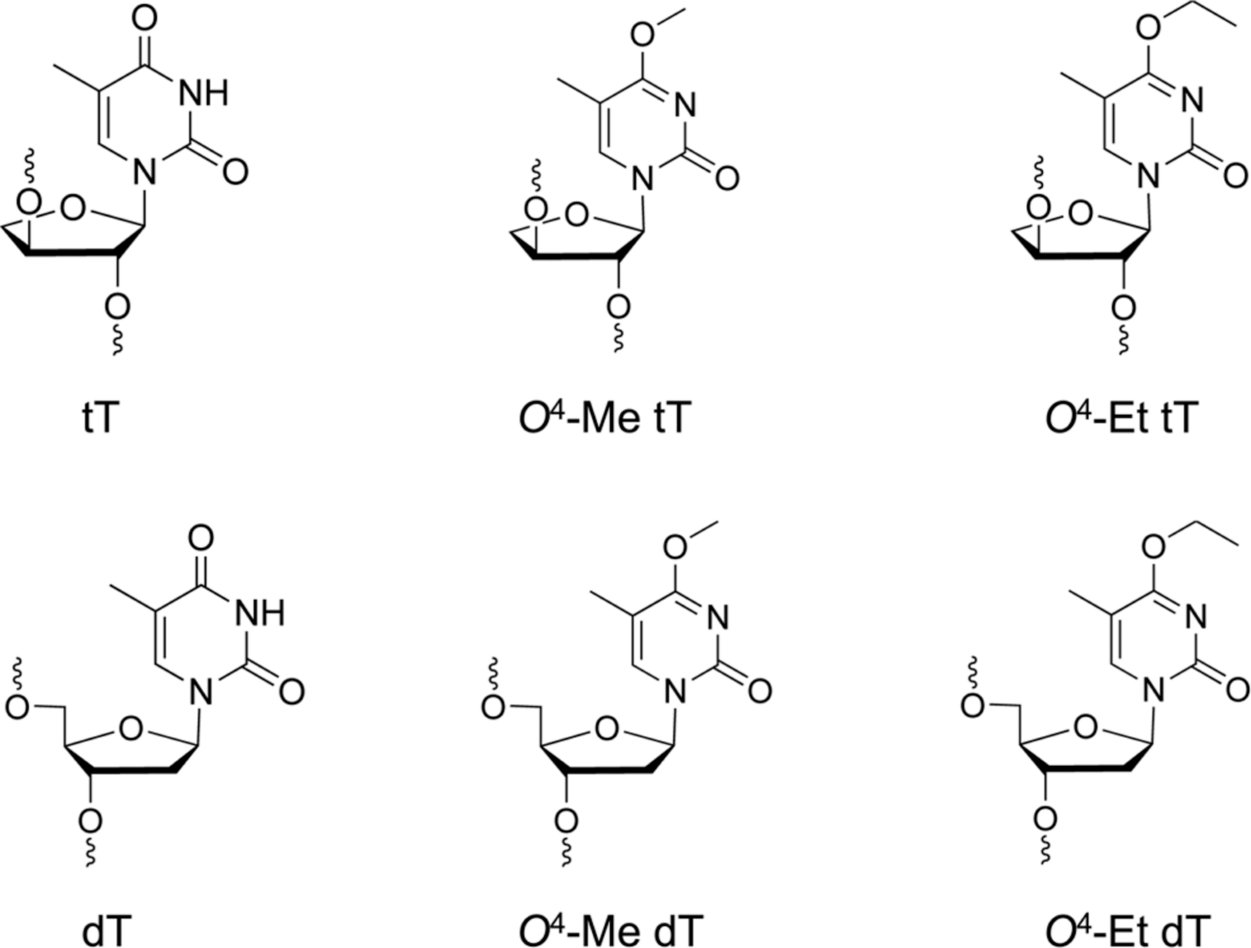
Structures of TNA T (tT), *O*^4^-Me tT, and *O*^4^-Et tT (Top Row) and the Corresponding 2′-Deoxynucleosides (Bottom Row)

**Scheme 2:**
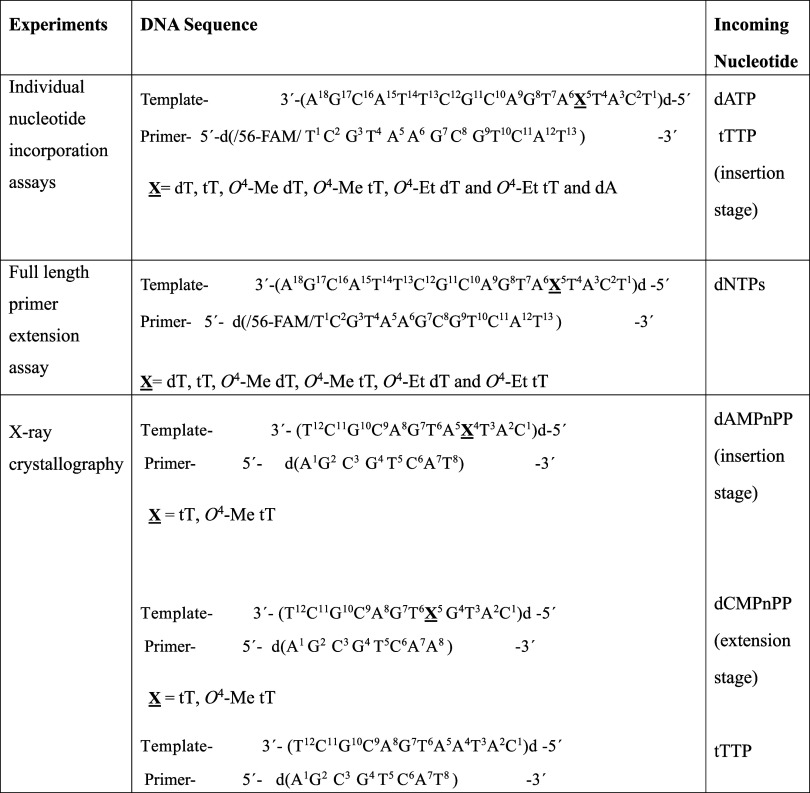
Template and Primer Sequences Used in the *In Vitro* hPol η-Catalyzed Replication Studies

In the present study, we synthesized DNA oligonucleotides containing a site-specific threose nucleotide (tT) including adducted thymine *O*^4^-alkylated tT (Schemes 1 and 2) and investigated *in vitro* synthesis of DNA past tT and *O*^4^-alkylated tT by hPol η. Biochemical assays in combination with X-ray crystallographic investigations of ternary hPol η complexes with tT-containing template DNA and incoming dNTPs in the insertion and extension steps of the catalytic cycle suggest successful nucleotidyl transfer reactions. The crystal structure of the ternary hPol η·DNA·tTTP complex provides insight into the inability of this pol to incorporate a TNA residue into the primer strand. Additionally, structural studies on hPol η ternary complexes with *O*^4^-methyl analogs of tT (*O*^4^-Me tT) in the template, trapped either at the insertion stage or extension stage with incoming dAMPnPP and dCMPnPP nucleotides, offer a better understanding of the more limited ability of this pol to synthesize past *O*^4^-alkylated TNA residues relative to tT. Overall, our study affords an expanded knowledge of TNA-templated DNA synthesis by an error-prone natural polymerase.

## Materials and Methods

### Preparation and Characterization of Oligo-2′-deoxynucleotides with Incorporated Native and Base-Adducted TNA Residues

The synthesis of the phosphoramidite derivatives of *O*^4^-Me tT (**5a**) and *O*^4^-Et tT (**5b**) is shown in Scheme S1 (please see Supporting Information for details of the methods). Starting from 3′-*O*-[4,4′-dimethoxytrityl-α-l-threofuranosyl] thymine (**1**),^6^ the 2′-hydroxyl group was then protected as a *tert*-butyldimethyl silyl (TBS) ether to give compound **2**. Subsequently, in a one-pot, two step reaction, the convertible *C*^4^-triazolyl derivate was prepared followed by the addition of methoxide or ethoxide to introduce the methyl (**3a**) or ethyl (**3b**) adduct at the *O*^4^-atom of thymine.^41,46^ The TBS group was then removed with fluoride treatment (**4a** and **4b**) followed by phosphitylation to produce the *O*^4^-Me tT (**5a**) and *O*^4^-Et tT (**5b**) phosphoramidites.

DNA oligonucleotides containing the *O*^4^-Me tT and *O*^4^-Et tT modifications were prepared by solid-phase synthesis using an Applied Biosystems 3400 DNA Synthesizer. Given the labile nature of the *O*^4^-thymine adducts, fast-deprotecting 3′-*O*-2′-deoxynucleoside phosphoramidites were used with phenoxyacetic anhydride as the capping reagent. DNA and TNA phosphoramidites were dissolved in anhydrous acetonitrile to a concentration of 0.15 M with an extended coupling time for the TNA phosphoramidites. Standard fast-deprotecting conditions with 0.05 M K_2_CO_3_/MeOH at room temperature or 10% v/v DBU in EtOH at 55 °C were employed for the oligonucleotides containing *O*^4^-Me tT and *O*^4^-Et tT, respectively. Purification by ion-exchange high-performance liquid chromatography (HPLC) was successful in acquiring the desired oligonucleotides as confirmed by MS. Control sequences were also synthesized with unmodified TNA and alkylated dT (see Supporting Information for synthesis and characterization details).

### hPol η Expression and Purification

hPol η was expressed and purified using a published protocol.^47,48^ hPol η, protein residues 1–432, in a pET28a plasmid construct (a gift originally from Dr. Wei Yang, NIDDK, National Institutes of Health, Bethesda, MD) was expressed using *Escherichia coli* BL21 gold (DE3) cells (Agilent Technologies, Santa Clara, CA). The bacterial culture was grown at 37 °C in Luria broth medium (Research Product International, Mt Prospect, IL) containing 100 μg/mL ampicillin (RPI) until the OD_600_ reached ∼0.6 and then shifted to 18 °C following addition of 0.5 mM final concentration of isopropyl β-d-1-thiogalactopyranoside (IPTG) to induce protein expression. After 18 h of incubation, cells were harvested and resuspended in a buffer containing 1 M NaCl, 20 mM imidazole, 5 mM β-mercaptoethanol, and protease inhibitor cocktail (Roche, South San Francisco, CA), and 20 mM Tris-HCl, pH 7.5, along with lysozyme at 1 mg/mL and DNase I at 50 μg/mL final concentration. Later, cells were lysed by a sonicator followed by high-speed centrifugation at 18,000 rpm for 45 min at 4 °C to separate cell debris. Filtered cell lysate was loaded onto a pre-equilibrated Ni-NTA His Trap HP 5 mL column (Cytiva Life Sciences, Marlborough, MA) using an AKTA pure 25 M (Cytiva Life Sciences, Marlborough, MA) for the binding of the N-terminal 6 × His-tagged protein at 4 °C. After washing the column with buffer containing 1 M NaCl, 50 mM imidazole, and 5 mM β-mercaptoethanol in 20 mM Tris-HCl (pH 7.5), the protein was eluted with 1 M NaCl, 300 mM imidazole, and 5 mM β-mercaptoethanol in 20 mM Tris-HCl (pH 7.5). Ni-NTA purified hPol η was concentrated using Amicon centrifugal filters with a membrane cutoff of 30 kDa (Millipore Sigma, Burlington, MA) and then buffer exchanged with 500 mM KCl, 3 mM dithiothreitol (DTT), 1 mM ethylenediaminetetraacetic acid (EDTA), 10% glycerol (v/v), and 20 mM Tris-HCl (pH 7.5) buffer. To cleave the N-terminal 6 × His-tag, PreScission protease (APExBIO, Houston, TX) was added as 1:100 units/μg of protein and incubated overnight at 4 °C. Later, protein was passed through Amicon centrifugal filters with molecular weight cutoff of 10 kDa (Millipore Sigma, Burlington, MA) to exchange the solution with 250 mM KCl, 10% glycerol (v/v), 3 mM DTT, and 1 mM EDTA in 20 mM 2-(*N*-morpholino)ethanesulfonic acid (MES) buffer, pH 6.0. Next, the protein was loaded on a HiTrap SP HP strong cation-exchange column (5 mL) (Cytiva Life Sciences, Marlborough, MA) and eluted using a 0–1 M KCl gradient. Finally, to remove remaining impurities and higher order oligomers, hPol η protein was passed through a size exclusion column, Superdex 200 (10/300 GL) (Cytiva Life Sciences, Marlborough, MA), pre-equilibrated with 500 mM KCl, 10% glycerol (v/v), and 3 mM DTT in 20 mM Tris-HCl buffer (pH 7.5). The protein purity was checked using 4–12% sodium dodecyl-sulfate polyacrylamide gel electrophoresis (SDS-PAGE). The monomeric protein mass was confirmed by liquid chromatography–mass spectrometry (LC–MS) analysis. The theoretical and observed masses for the cleaved protein were 48,556.77 and 48,580.56 Da, respectively. The protein concentration was determined by UV absorbance at 280 nm (ε_280_, 1 mg/mL ∼ 1.03). For crystallization, hPol η was concentrated to 2–3 mg/mL. For long-term storage, the protein was flash frozen in liquid nitrogen and stored at −80 °C.

### DNA Replication Assays

Site-specifically, α-l-(3′-2′)-threofuranosyl thymidine (tT), *O*^4^-Me tT- or *O*^4^-Et tT-modified 18-mer oligodeoxynucleotides (Schemes 1 and 2, template strand), and corresponding 5′-FAM-labeled 13-mer complementary oligodeoxynucleotide (primer strand) were first purified through a reversed phase HPLC C_18_ column, Gemini C_18_ 250 mm × 10 mm (Phenomenex, Torrance, CA), in a buffer containing 0.1 M ammonium formate using an acetonitrile gradient and subsequently lyophilized. Next, both template and primer strands were annealed at room temperature in a 1:1 molar ratio in buffer containing 50 mM NaCl, 50 μM EDTA (sodium salt), and 20 mM Tris (pH 7.5) for 10 min and then stored at −20 °C. The corresponding template strands containing dT, *O*^4^-Me dT, or *O*^4^-Et dT were also annealed with primer as a control. To assay incorporation of dNMP opposite template tT, *O*^4^-Me tT, *O*^4^-Et tT, dT, *O*^4^-Me dT or *O*^4^-Et dT, a 10 nM concentration of purified hPol η was incubated with 150 nM of TNA-containing template-primer DNA in 50 mM NaCl, 5 mM DTT, 5% glycerol (v/v), 5 mM MgCl_2_, 100 mg/mL bovine serum albumin (BSA), and 50 mM Tris-HCl (pH 7.5), in a 50 μL volume and pre-equilibrated at 37 °C for 10 min before the addition of 50 μM dATP, dCTP, dGTP, or dTTP in a separate reactions. Reaction solutions were mixed and incubated at 37 °C for 40 min. At time points 0, 2, 5, 10, 20, and 40 min, 3.5 μL aliquots of each reaction were mixed with 6.5 μL of quench solution containing 95% formamide and 20 mM EDTA. Thereafter, 10 μL of 2 × TBE-urea-bromophenol blue and xylene cyanol containing loading buffer (Invitrogen, Waltham, MA) was added to each sample, followed by heating at 95 °C for 4 min to denature the sample. Next, samples were centrifuged, and 6 μL aliquots of each reaction mix were loaded into wells in a 15% TBE-urea gel (7 M urea) (Invitrogen, Waltham, MA) to separate products at 0, +1, or +2 primer sites by gel electrophoresis. Gels were visualized using a Bio-Rad ChemiDoc imaging system (Bio-Rad Laboratories, Hercules, CA).

For full-length primer extension assays, a previously published protocol was used.^41^ In short, TNA and non-TNA-containing template-primer DNA as described in the previous section (Schemes 1 and 2) were incubated with hPol η at 37 °C for 10 min in a buffer containing 50 mM NaCl, 5 mM DTT, 5% glycerol (v/v), 5 mM MgCl_2_, 100 mg/mL bovine serum albumin (BSA), and 50 mM Tris-HCl (pH 7.5). Subsequently, dATP, dCTP, dGTP, and dTTP nucleotides were added to each sample tube up to a 1 mM final concentration and the reaction was monitored for 1–2 h at 37 °C for dNTPs incorporation.

We also performed replication assays using TNA-thymine nucleoside triphosphate (tTTP) as the incoming nucleotide opposite the native DNA nucleotide in the template strand. Briefly, hPol η was incubated with unmodified template-primer DNA (Scheme 2) at 37 °C for 10 min, followed by addition of tTTP or dTTP nucleotides at different concentrations as indicated in the Results section, and the reaction was monitored for 2 h before quenching and running reaction products on a TBE-Urea gel.

### Crystallization of Ternary hPol η Complexes with TNA-Containing DNA Template-Primer Duplexes and Nonhydrolyzable dNMPnPPs

TNA-modified template oligodeoxynucleotides 5′-d(CATXATGACGCT)-3′ (X = tT, *O*^4^-Me tT or *O*^4^-Et tT) (for insertion stage analyses), 5′-d(CATGXTGACGCT)-3′ (X = tT or *O*^4^-Me tT or *O*^4^-Et tT) (for extension stage analyses), and corresponding complementary primer oligodeoxynucleotides, 5′-d(AGCGTCAT)-3′ and 5′-d(AGCGTCAA)-3′ (Schemes 1 and 2), were first purified using a reversed phase HPLC C_18_ column, Gemini C_18_ 250 mm × 10 mm (Phenomenex, Torrance, CA), in a buffer containing 0.1 M ammonium formate with an acetonitrile gradient and subsequently concentrated by lyophilization. To anneal insertion and extension stage template-primer pairs, the corresponding template and primer strands were mixed in an equimolar ratio in a buffer containing 20 mM HEPES, pH 7.5, and 100 mM NaCl and subsequently heated for 5 min at 85 °C, followed by slow cooling and storage at 4 °C. Next, to make the binary complex of hPol η and TNA-containing DNA substrate, protein in 500 mM KCl, 10% glycerol (v/v), 3 mM DTT, and 20 mM Tris-HCl (pH 7.5) and DNA were mixed in a 1:1.1 molar ratio, followed by incubation at room temperature for 10–15 min. To reduce the salt concentration to 125 mM KCl and the glycerol content below 3% (v/v), the protein–DNA mix was diluted 3-fold with buffer containing final concentrations of 5 mM MgCl_2_, 3 mM DTT, and 20 mM Tris-HCl (pH 7.5). All samples were concentrated using 10 kDa cutoff Amicon centrifugal filters (Millipore Sigma, Burlington, MA) to achieve approximately 2 mg/mL final protein concentration of the binary complex. To assemble insertion or extension stage ternary complexes, dNTP nonhydrolyzable analogs 2′-deoxyadenosine-5′-[(α,β)-imido]triphosphate (sodium salt; dAMPnPP) or 2′-deoxycytidine-5′-[(α,β)-imido]triphosphate (sodium salt; dCMPnPP), (Jena BioScience, Jena, Germany), respectively, were added separately up to a 10 mM final concentration to the hPol η -DNA binary complex. Complexes were kept on ice for 30 min before setting up 24-well crystallization plates using the hanging drop vapor diffusion method. The reservoir buffer contained poly(ethylene glycol) monomethyl ether 2000 (PEG MME 2000) (Hampton Research, Aliso Viejo, CA) (14–24%, v/v), 5 mM MgCl_2_, and 0.1 M MES hydrate (Millipore Sigma, Burlington, MA) at pH 5.6, 6.0, or 6.5. For each drop, 0.8 μL of ternary complex was mixed with 0.8 μL of a reservoir solution. Plates were incubated either at 18 °C or at room temperature. Crystals were observed for tT and *O*^4^-Me tT-containing DNA templates at the insertion and extension stages at different pH values and PEG concentrations after 2 to 4 days. Diffraction-quality crystals were obtained after 1 to 2 weeks.

### Crystallization of a Ternary hPol η Complex with a DNA Template-Primer Duplex and Incoming tTTP

To produce the hPol η ternary complex with incoming tTTP, the DNA template 5′-d(CATAATGACGCT)-3′ was annealed with complementary primer 5′-d(AGCGTCAT)-3′ (Scheme 2) in 1:1 molar ratio in a buffer containing 20 mM HEPES, pH 7.5 and 100 mM NaCl by heating for 5 min at 85 °C, followed by slow cooling and storage at 4 °C. Next, following the protocol as described for TNA-containing DNA substrate, the binary complex of hPol η with annealed template-primer duplex was prepared. To make the ternary complex, tTTP was added to the hPol η-DNA binary complex to a final concentration of 10 mM. Following half an hour of incubation on ice, 1 μL of ternary complex was mixed with 1 μL of reservoir buffer which contained varying concentrations (14–24%, v/v) of poly(ethylene glycol) monomethyl ether 2000 (PEG MME 2000; Hampton Research, Aliso Viejo, CA), 5 mM CaCl_2_, and 0.1 M MES hydrate (Millipore Sigma, Burlington, MA) at pH 5.6 and subsequently set up for crystallization in a 24-well plate using the hanging drop vapor diffusion method at 18 °C. Diffraction-quality crystals were obtained in 1 week.

### X-ray Diffraction Data Collection, Structure Determination, and Refinement

Diffraction data for crystals of TNA-containing hPol η·DNA·dNTP complexes and a ternary complex with incoming tTTP were collected on beamline 21-ID-F at LS-CAT, Advanced Photon Source (APS), Argonne National Laboratory (Argonne, IL). For crystals of *O*^4^-Me tT-containing complexes, diffraction data were collected on beamline ID 23-2 at the European Synchrotron Radiation Facility (ESRF, Grenoble, France) (Table 1). For data sets collected at the APS, data processing, including integration and scaling, was done with HKL2000 (HKL Research, Charlottesville, VA).^49^ Data sets collected at ESRF were initially processed in CCP4 suite of programs including AIMLESS scaling^50^ and Xia-2 for data reduction.^51^ Later, the SCALEPACK2 and AIMLESS programs (CCP4 suite) were used to get average intensities.^50,52^ The ternary complex structures corresponding to each TNA-containing DNA template-primer construct were determined by molecular replacement with Phaser^53^ using the previously published complex structures with PDB ID 8UJT or 8UJV as a search model.^47^ Initial rounds of rigid body refinement followed by restrained refinement were performed using Phenix.^54^ Further refinement and model building were done using Phenix^54^ and Coot.^55^ Figure illustrations were generated with PyMOL (The PyMOL Molecular Graphics System, Version 2.5.2, Schrödinger LLC).

**Table 1:** Selected Crystal Data, X-ray Data Collection, and Refinement Statistics

complex	tT (insertion)	tT (extension)	*O*^4^-Me tT (insertion)	*O*^4^-Me tT (extension)	dA:tTTP (insertion)
PDB entry	9CHW	9CI9	9CJ9	9CIH	9CIQ
SB grid entry	1124	1125	1126	1127	1128
Data Collection					
X-ray source	APS LS-CAT	APS LS-CAT	ESRF	ESRF	APS LS-CAT
	21-ID-F	21-ID-F	ID23-2	ID23-2	21-ID-F
wavelength [Å]	0.97872	0.97872	0.96770	0.87313	0.97872
space group	*P*6_1_	*P*6_1_	*P*6_1_	*P*6_1_	*P*6_1_
Unit Cell					
*a* [Å]	98.69	98.90	98.08	98.47	99.48
*b* [Å]	98.69	98.90	98.08	98.47	99.48
*c* [Å]	82.06	81.54	78.66	81.9	81.20
α/β/γ [deg]	90, 90, 120	90, 90, 120	90, 90, 120	90, 90, 120	90, 90, 120
resolution [Å]	50–2.16^a^ (2.20–2.16)	50–2.10^a^ (2.14–2.10)	50–2.98^a^ (3.06–2.98)	50–2.15^a^ (2.21–2.15)	50–2.80^a^ (2.90–2.80)
reflections	23,956 (1222)	26,703 (947)	8,479 (653)	24,623 (1809)	11,361 (1122)
*R*_sym_	0.042 (0.085)	0.100 (0.494)	0.133 (1.860)	0.104 (0.737)	0.123(0.892)
*R*_pim_	0.032 (0.068)	0.049 (0.287)	0.059 (0.818)	0.045 (0.319)	0.056 (0.409)
*I*/σ (I)	29.20 (12.95)	19.30 (2.34)	10.9 (0.8)	11.4 (2.3)	15.0 (1.75)
completeness [%]	97.8 (100.0)	98.5 (70.8)	95.7 (100.0)	99.9 (100.0)	99.7 (100.0)
redundancy	2.5 (2.5)	5.1 (3.8)	6.1 (6.1)	6.3(6.2)	5.7 (5.7)
CC_1/2_	0.993 (0.985)	0.998 (0.720)	0.997 (0.393)	0.998 (0.768)	0.990(0.726)
Refinement					
no. of complexes per asymmetric unit	1	1	1	1	1
*R*_cryst_ [%]	14.3	15.8	22.0	17.0	17.8
*R*_free_ [%]	21.0	20.6	34.8	23.2	26.8
RMS deviation					
bond length [Å]	0.007	0.007	0.009	0.008	0.009
bond angles [deg]	0.9	0.9	1.1	0.9	1.4
Ramachandran [%] (PROCHECK)					
favored	90.8	91.8	80.8	92.3	87.1
allowed	8.4	7.4	17.8	7.2	11.9
generous	0.8	0.8	0.8	0.5	0.8
outliers	0.0	0.0	0.5	0.0	0.3
B-factor [Å^2^]	17.4	22.6	29.9	21.5	53.9
no. of atoms	4,202	4,083	3,814	4,096	3,836
No. of Residues					
protein	435 (chain A)	431 (chain A)	431 (chain A)	431 (chain A)	432 (chain A)
DNA	19 (chains T, P)	19 (chains T, P)	19 (chains T, P)	19 (chains T, P)	19 (chains T, P)
water	340	309	34	263	37

a Statistics for the highest resolution shell are shown in parentheses.

## Results

### Experiments Probing DNA Synthesis across Template tT and Insertion of tTTP by hPol η

**Figure 1:**
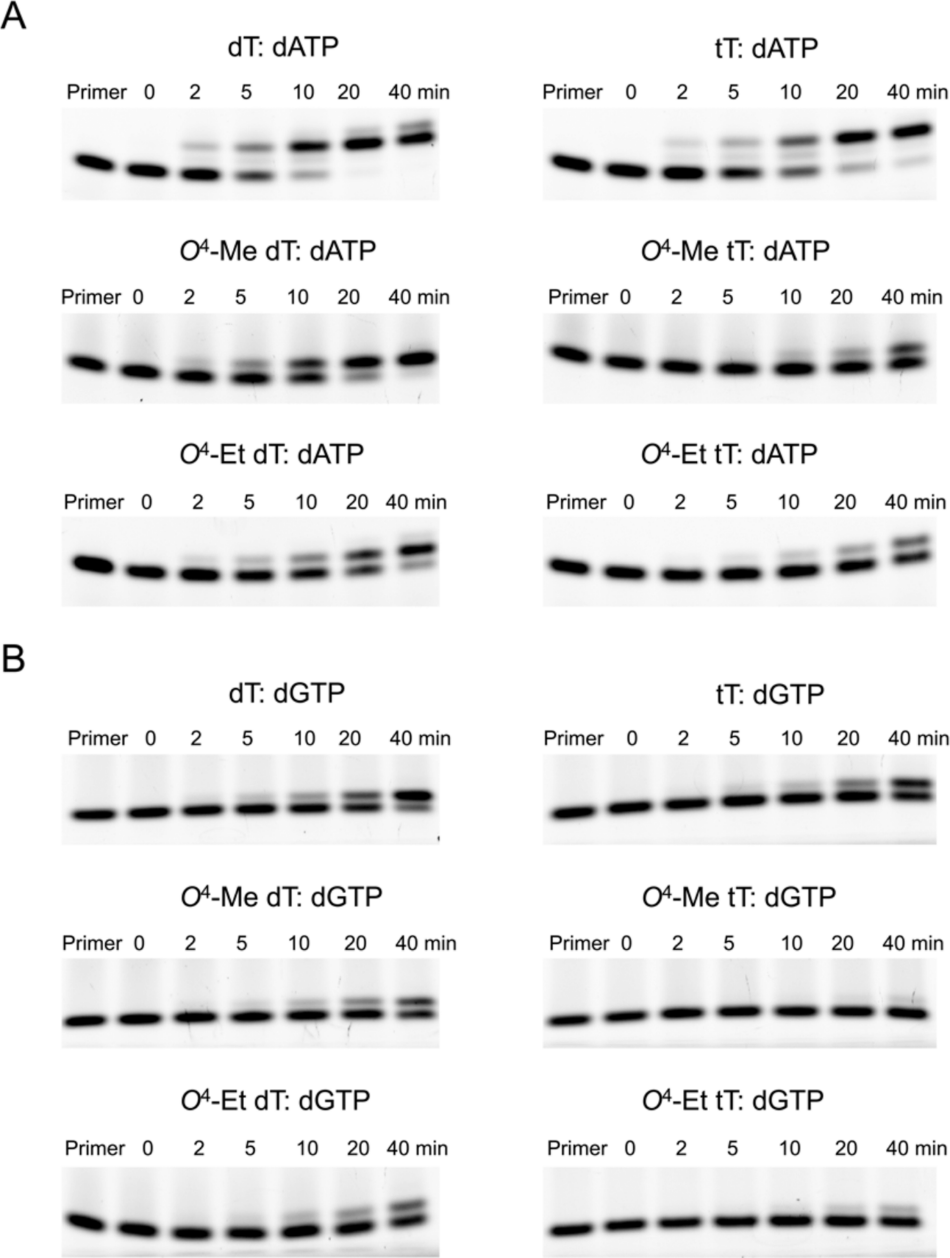
Incorporation of purine nucleotides opposite tT and base-adducted tT in a DNA template by hPol η and comparison with the corresponding 2′-deoxynucleotides as analyzed by 15% TBE-urea PAGE. The gel images depict incorporation of (A) dATP and (B) dGTP opposite tT, top, *O*^4^-Me tT, center, and *O*^4^-Et tT, bottom (right panels) as well as opposite the corresponding dTs (left panels). In each assay, 10 nM hPol η was incubated with 150 nM DNA template:primer at 37 °C, followed by addition of 50 μM dATP or dGTP. At the indicated time points, aliquots were removed and mixed with quencher. In each panel, the first lane is the 5′-FAM-labeled primer band before annealing with the template. Each replication experiment is done in triplicate as independent sets of experiments.

To investigate the ability of hPol η to synthesize past a TNA thymidine (tT) in a DNA template, individual nucleotide incorporation assays were performed on an annealed tT-modified 18-mer DNA template and 13-mer 5′-FAM fluorophore-labeled primer strands (Scheme 2). hPol η inserted the correct dAMP nucleotide opposite tT but with a slightly lower efficiency compared to template dT (Figure 1A). After 20 min of incubation with dATP, nearly all of the primer was consumed in the case of control dT to form the +1 and +2 primer site additions. By comparison, ∼10% of the primer remained unutilized at that time point with tT. Furthermore, hPol η also bypassed adducted tT template residues such as *O*^4^-Me tT or *O*^4^-Et tT, albeit with lower efficiencies than the corresponding adducted DNA residues and tT. With *O*^4^-Me dT-containing template DNA, ∼90% primer was utilized to make the +1 addition after a 20 min incubation; with *O*^4^-Me tT-containing template DNA, only ∼10% of primer was extended after 20 min. Similarly, with the *O*^4^-Et dT-containing template, ∼50% of primer was extended after a 20 min incubation, whereas only ∼10% of primer was extended with *O*^4^-Et tT-containing DNA (Figure 1A).

**Figure 2:**
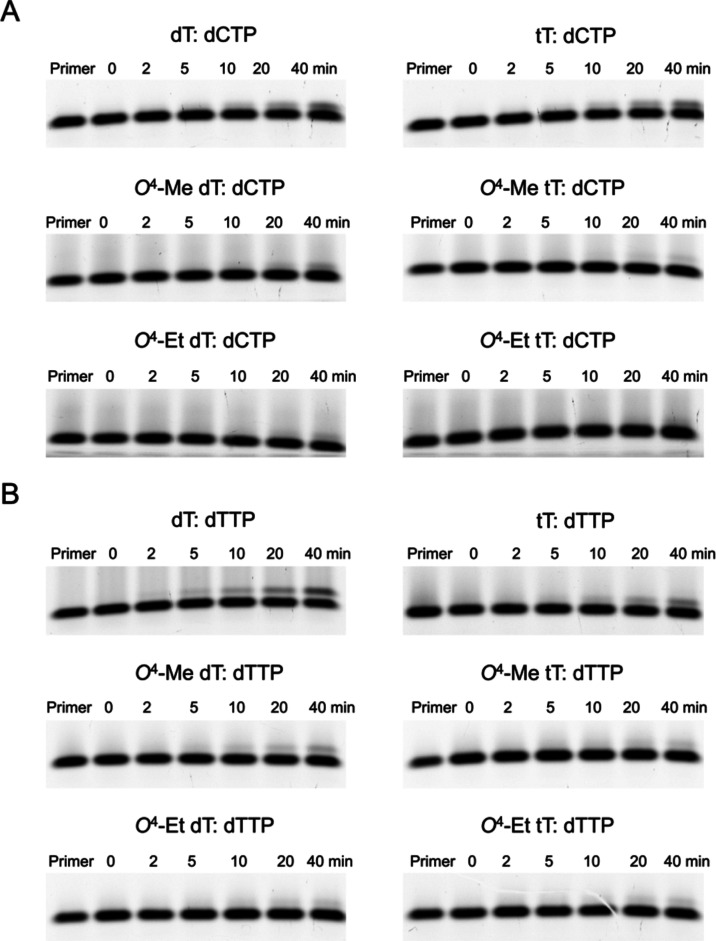
Incorporation of pyrimidine nucleotides opposite tT and base-adducted tT in a DNA template by hPol η and comparison with the corresponding 2′-deoxynucleotides as analyzed by 15% TBE-urea PAGE. The gel images depict incorporation of (A) dCTP and (B) dTTP opposite tT, top, *O*^4^-Me tT, center, and *O*^4^-Et tT, bottom (right panels), as well as opposite the corresponding dTs (left panels). In each assay, 10 nM hPol η was incubated with 150 nM DNA template:primer at 37 °C, followed by addition of 50 μM dCTP or dTTP. At the indicated time points, aliquots were removed and mixed with quencher. In each panel, the first lane is the 5′-FAM-labeled primer band before annealing with the template. Each replication experiment is done in triplicates as independent sets of experiments.

We noticed that hPol η also misincorporates dGMP, dCMP, and dTMP opposite tT, *O*^4^-Me tT or *O*^4^-Et tT in a DNA template (Figures 1B and 2A,B). Thus, dGMP insertion opposite tT-containing DNA occurs for ∼30% of the primer in 20 min. However, only ∼5% of primer undergoes insertion opposite *O*^4^-Me tT or *O*^4^-Et tT-containing DNA after a 20 min incubation (Figure 1B). Again, the incorporation efficiencies were slightly lower than those opposite the corresponding all-DNA template where ∼40% primer was extended by one nucleotide opposite dT and ∼20% opposite *O*^4^-Me or *O*^4^-Et dT. After a similar incubation period, incorporation of dCMP or dTMP opposite tT was observed for ∼5% of primer whereas their incorporation opposite *O*^4^-Me tT or *O*^4^-Et tT remained negligible (Figure 2A,B). The dCMP and dTMP insertion efficiencies were similar to those of dT or the adducted dTs in all-DNA templates.

**Figure 3:**
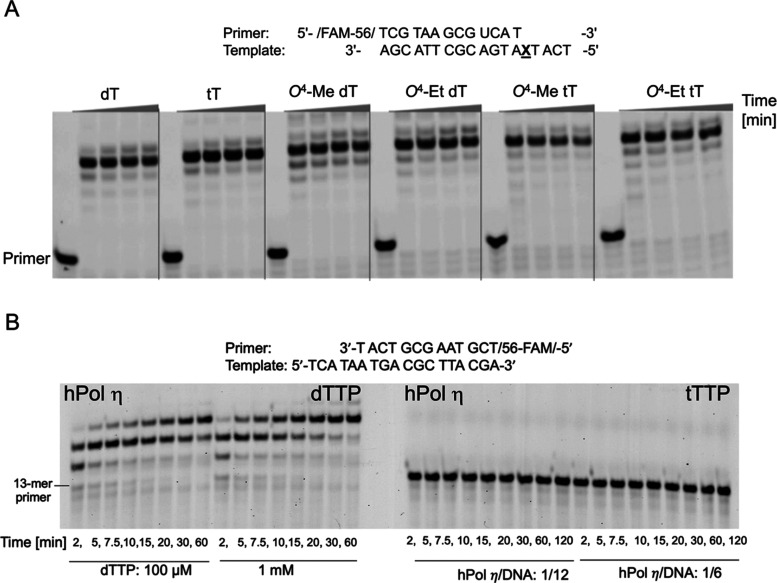
hPol η mediated DNA replication assays in the presence of all four nucleotides and inability of the pol to use TNA triphosphate as a substrate. (A) Full-length primer extension assays showing dNTP incorporation opposite and past tT, *O*^4^-Me tT, *O*^4^-Et tT and the corresponding 2′-deoxynucleotides. (B) hPol η incorporates dTTP (left) but not tTTP (right) opposite template dA. The reaction was monitored for 120 min, and at the indicated time points, aliquots were removed and mixed with quencher.

Following incubation with a mixture of dNTPs, hPol η also mediated full-length primer extension past tT, *O*^4^-Me tT, and *O*^4^-Et tT residues in a DNA template (Figure 3A). Conversely, hPol η was unable to incorporate a TNA residue into a DNA primer (tTTP opposite dA; Figure 3B, right), contrary to the standard incorporation of dTMP into the primer opposite template dA (Figure 3B, left).

### Crystal Structures of Ternary hPol η Complexes with Template tT or Incoming tTTP

**Figure 4:**
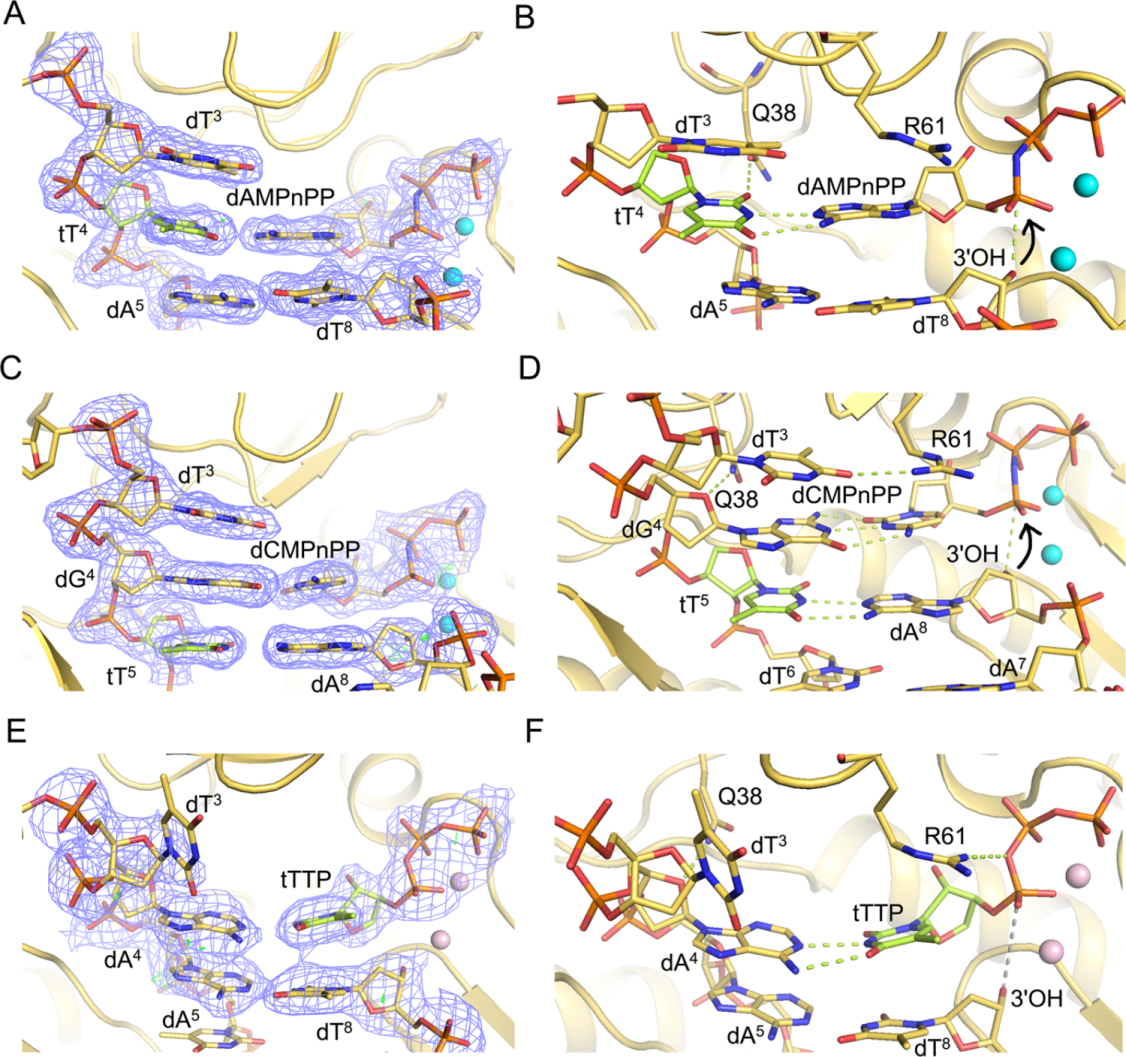
Views of the active-site region of hPol η ternary complexes with either a 3′ → 2′ linked tT residue in the DNA template strand at the insertion and extension stages and incoming nonhydrolyzable dNMPnPPs, or with incoming TNA thymidine triphosphate (tTTP) opposite an all-DNA template strand. (A) Quality of the final 2Fo-Fc Fourier sum electron density (blue mesh, contoured at 1σ) for the (B) insertion stage complex with template tT^4^ opposite incoming dAMPnPP. (C) Quality of the final 2Fo-Fc Fourier sum electron density for the (D) extension stage complex with template dG^4^ opposite the incoming dCMPnPP and stacked onto tT^5^:dA^8^. (E) Quality of the final 2Fo-Fc Fourier sum electron density for the (F) ternary complex with incoming tTTP opposite the template dA^4^. DNA and TNA carbon atoms are colored in yellow and green, respectively; Mg^2+^ and Ca^2+^ ions are cyan and pink spheres, respectively, and H-bonds are green dotted lines.

We determined three X-ray crystal structures of ternary hPol η complexes with a tT residue either in the DNA template strand or as the incoming nucleoside triphosphate (tTTP) (Scheme 2). Illustrations of the active-site regions in these complexes are depicted in Figure 4 and selected crystal data, data collection, and refinement parameters are summarized in Table 1.

In both the insertion (Figure 4A,B) and extension state (Figure 4C,D) complexes, tT template residues and complementary incoming and primer nucleotides are well resolved in the electron density maps. The four-carbon tetrose sugar with 3′ → 2′ phosphodiester linkage results in a locally tighter spacing between adjacent phosphates (P–P) of 5.81 Å in the insertion complex compared to the preceding (6.63 Å) and following steps (6.85 Å). In the extension complex, the corresponding distances are 6.08, 6.77, and 6.51 Å, respectively. As expected, the tetrose sugar adopts a C4′-*exo* pucker in both complexes.^2,22,23,40^

In the insertion complex with an incoming dAMPnPP nucleotide base paired opposite the template tT^4^ residue, the 3′-OH of the terminal dT^8^ primer residue is positioned at 3.30 Å from Pα of the incoming nucleotide (Figure 4B, curved arrow), consistent with facile nucleotide insertion by hPol η opposite a TNA residue in the template strand. Similarly, in the extension complex with base-pairing between template tT^5^ and primer dA^8^ as well as between dG^4^ and incoming dCMPnPP, the 3′-OH of the terminal dA^8^ primer residue is positioned at 3.80 Å from the Pα of the incoming dCMPnPP (Figure 4D, curved arrow). These structural observations are in line with the *in vitro* assays of the incorporation of individual nucleotides and primer extension data that demonstrate that hPol η can readily and correctly bypass a TNA residue. In the two complexes, template tT forms two H-bonds with either incoming dAMPnPP (insertion complex) or primer dA (extension complex). At the extension stage, template dG^4^ residue establishes three H-bonds with incoming dCMPnPP. In both complex structures, two Mg^2+^ ions coordinate with incoming phosphates, primer terminal 3′-OH, and protein residues. The side chain of hPol η Glu38 forms a H-bond either with template tT^4^*O*^2^ (insertion complex) or dG^4^ O4′ and a water-mediated H-bond with tT^5^ (extension complex). The guanidino moiety of Arg61 stays within H-bonding distance from Pα of the incoming nucleotide (3.31 Å, insertion complex) or from *O*^4^ of unpaired template dT^3^ (2.85 Å, extension complex).

We also determined the crystal structure of a ternary complex of hPol η with a DNA template-primer duplex and tTTP opposite dA in the active site (Figure 4E). Unlike the complexes with tT in the template strand, this structure reveals that the 3′-OH group of the terminal dA^8^ primer residue is positioned quite far from the Pα atom of tTTP (4.6 Å, Figure 4F). This explains the inability of hPol η to incorporate TNA into a DNA primer opposite a DNA template strand, as seen in the *in vitro* replication assay (Figure 3B). Although incoming tTTP is paired with template dA^4^ in the presence of two Ca^2+^ ions, the 3′ → 2′ connectivity of TNA precludes a close enough approach of the terminal 3′-OH of the primer vis-à-vis the α-phosphate of tTTP. The pucker of the tTTP tetrose is C4′-*exo*, like in the template strand tT residues seen in the insertion and extension state complexes.

### Crystal Structures of Ternary hPol η Complexes with Base-Adducted Template tT

**Figure 5:**
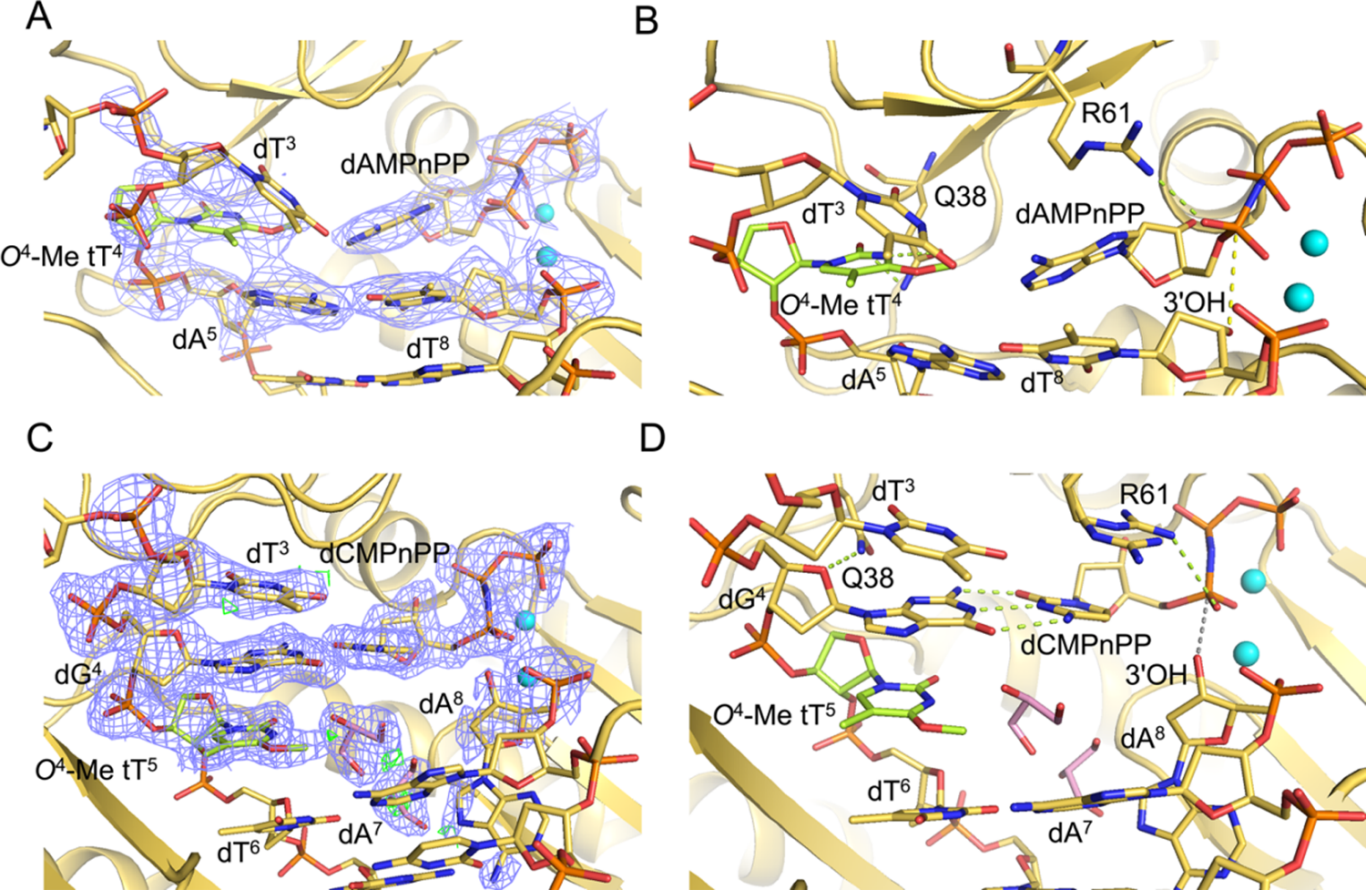
Views of the active-site region of hPol η ternary complexes with a 3′ → 2′ linked *O*^4^-Me tT in the DNA template strand at the insertion and extension stages and incoming nonhydrolyzable dNMPnPPs. (A) Quality of the final 2Fo-Fc Fourier sum electron density (blue mesh, contoured at 1σ) for the (B) insertion stage complex with template *O*^4^-Me tT^4^ opposite incoming dAMPnPP. (C) Quality of the final 2Fo-Fc Fourier sum electron density for the (D) extension stage complex with template dG^4^ opposite the incoming dCMPnPP and stacked onto *O*^4^-Me tT^5^:dA^8^. DNA and TNA carbon atoms are colored in yellow and green, respectively; Mg^2+^ ions are cyan spheres, and H-bonds are green dotted lines. Two glycerol molecules were modeled opposite the modified nucleotide and at an adjacent location in the extenstion stage complex and are shown with carbon atoms colored in pink.

We also determined crystal structures of hPol η ternary complexes with an *O*^4–^Me tT in the template strand, trapped either at the insertion stage and opposite incoming nonhydrolyzable dAMPnPP, or at the extension stage and opposite primer dA and stacked onto template dG paired to incoming dCMPnPP. Illustrations of the active-site regions in the two complexes are depicted in Figure 5, and selected crystal data, data collection, and refinement parameters are summarized in Table 1. In both complexes, the *O*^4^-Me group of the adducted tT adopts the *syn* orientation, and the sugar pucker of TNA residues in the template strand is C4′-*exo*, matching the conformation of tetrose residues with the native thymine base in the structures of insertion and extension complexes.

In the insertion complex, the *O*^4^-Me tT template base and the incoming adenosine do not engage in H-bonding. The two base moieties exhibit considerable buckling, and there is only minimal stacking between the replicating *O*^4^-Me tT^4^ and dAMPnPP nucleotides and the adjacent dA^5^:dT^8^ base pair (Figure 5A,B). The thymine moiety of the 5′-adjacent dT^3^ template residue is shifted into the major groove and its *O*^2^ keto oxygen is H-bonded to the *N*^6^ amino group of the incoming dAMPnPP (2.9 Å). The distance between the 3′-OH group of the terminal primer residue dT^8^ and Pα (3.5 Å, Figure 5B) of incoming nucleotide appears not to hamper bypass synthesis as the *in vitro* replication assays with hPol η showed insertion opposite *O*^4^-Me tT and extension of the primer to full length.

With regard to the latter observation, it is noteworthy that the crystal structure of the extension complex revealed an orphaned, intrahelical *O*^4^-Me tT^5^ at the −1-position that stacked with adjacent template bases dG^4^ and dT^6^. However, primer dA^8^ is rotated into the minor groove and removed from the template-primer duplex base stack (Figure 5C,D). The resulting gap is filled with a glycerol molecule and water; the distance between 3′-OH of the terminal primer residue dA^8^ and Pα of the incoming dCMPnPP is increased to 4.0 Å, thereby precluding a nucleophilic attack (Figure 5D).

## Discussion

Considerable efforts have been made to evaluate ligand binding and catalytic function of TNA molecules using *in vitro* selection methods.^13,56^ Thus, following enzyme screening and engineering, several DNA-dependent TNA pols such as Therminator and Kod-RSGA,^31,35^ and TNA-dependent DNA pols (TNA reverse transcriptase) such as SuperScript II and Bst^32,36^ were investigated for efficient and faithful transfer of genetic information back and forth between TNA and DNA. Among these, an engineered B-family Bst pol functions as a TNA reverse transcriptase with superior activity that efficiently and faithfully copies TNA into DNA.^32^ A structural study of the Bst TNA reverse transcriptase captured the binary complex between Bst and a TNA template-DNA primer duplex (PDB ID 6MU5).^40^ Tetroses in the template strand adopted the C4′-*exo* pucker with an average distance between adjacent phosphates of ca. 5.7 Å. 2′-Deoxyriboses in the primer strand adopted the C2′-*endo* pucker with an average distance between phosphates of ca. 6.6 Å.

The Y-family hPol η is a specialized TLS DNA pol with a relatively large, flexible active site and a tolerance for a wide variety of DNA lesions while maintaining base selectivity in terms of the incoming nucleotide.^41,42,57^ hPol η can insert both dNTPs and rNTPs opposite DNA and RNA templates and constitutes a major reverse transcriptase in cellular environments.^43,57,58^ We were therefore interested in evaluating the tolerance of hPol η toward a backbone different from that of DNA and RNA combined with both standard and adducted nucleobases.

We first assessed hPol η’s ability to synthesize DNA across and beyond standard and modified tT nucleosides in a DNA template. hPol η efficiently bypassed TNA-containing DNA whereby the correct nucleotide dAMP was inserted opposite tT or *O*^4^-alkylated tTs with greater efficiency than the incorrect dGMP or other dNTPs. Overall, incorporation efficiencies for TNA-modified templates were lower than those for the corresponding all-DNA templates. Furthermore, for adducted tTs, dAMP insertion was lower than that of unadducted tT, although the DNA primer was extended to full length in all cases. Similar observations for incorporation efficiency were made previously for *O*^4^-ethylated dT where steady-state experiments indicated dAMP incorporation with greatest efficiency followed by dGMP incorporation, whereas dCMP/dTMP were incorporated with the lowest efficiency.^41^ However, for *O*^4^-methylated dT, dGMP and dAMP were incorporated with similar efficiencies by hPol η^41^ (0.19 ± 0.01 vs 0.18± 0.03 μM^–1^ s^–1^*K*_cat_/*K*_m_ value, respectively), and dGMP was incorporated with 75-fold higher efficiency by yeast DNA pol η^59^ (7.6 × 10^–3^ vs 0.57 μM^–1^ min^–1^*K*_cat_/*K*_m_ value, respectively). By comparison, human polymerase κ predominantly inserted dAMP opposite *O*^4^-methylated dT, followed by dCMP, dTMP, and dGMP.^59^ In a previous study, Washington and co-workers suggested human vs yeast DNA polymerase η differences in terms of nucleotide incorporation and binding affinity.^60^ Furthermore, incorporation differences may be influenced by sequence in some cases^61,62^ or damage-specific.^63^ Overall, the preferential incorporations of dAMP opposite tT or adducted tT showcase hPol η’s inherent ability of faithful nucleotide incorporation opposite a host of modified and lesioned template residues,^42,47,64^ and in the present case, a xeno nucleic acid with an unnatural sugar–phosphate backbone.

**Figure 6:**
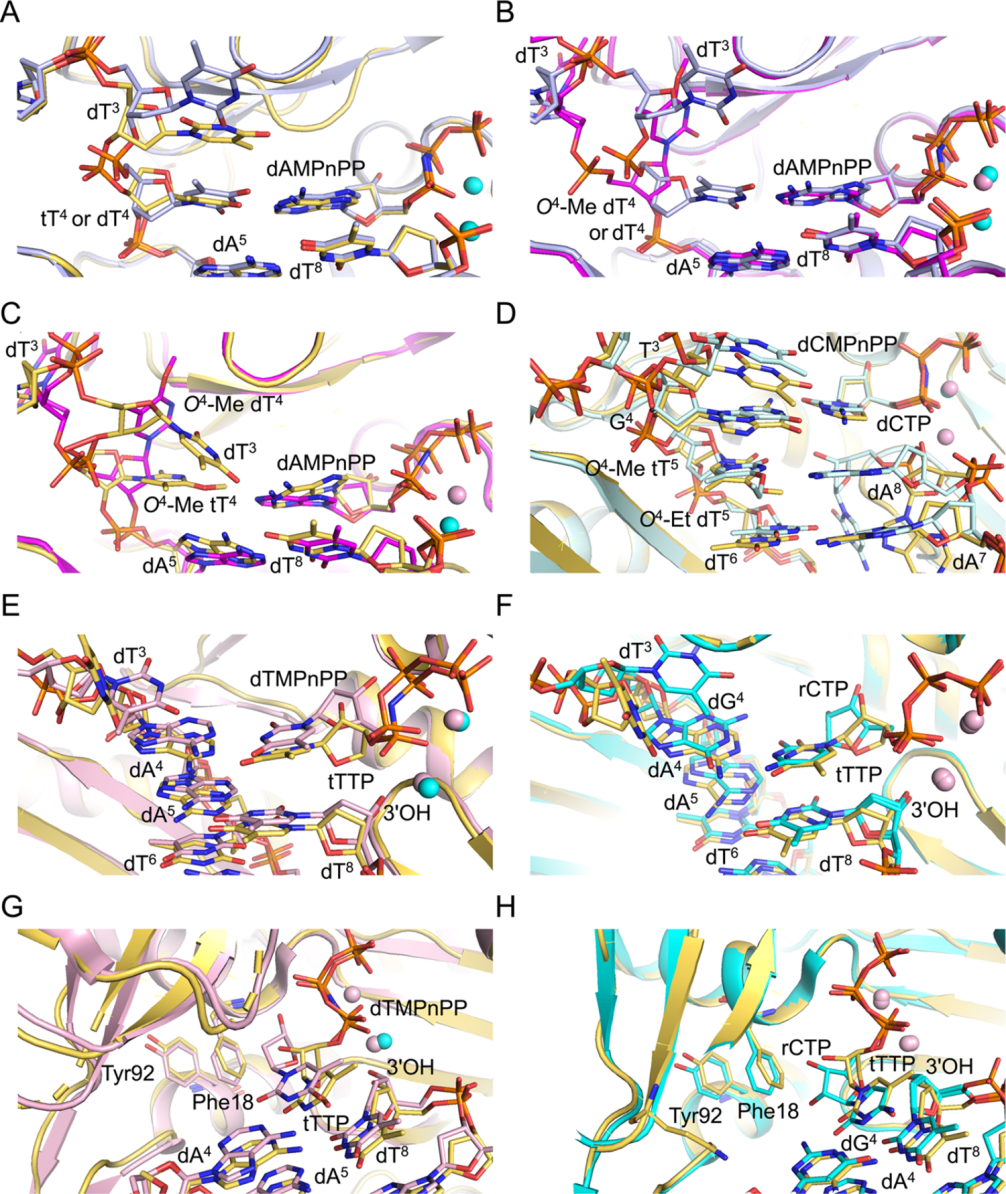
Overlays of crystal structures of ternary hPol η complexes with all-DNA or TNA-modified template strands with thymine or *O*^4^-Me or -Et adducted thymine nucleobases. (A) Comparison between insertion complexes featuring either template tT^4^ (yellow carbons, this work) or dT^4^ (gray carbons, PDB ID 3MR2(42)) opposite incoming dAMPnPP. (B) Comparison between insertion complexes featuring either template dT^4^ (gray carbons, PDB ID 3MR2) or *O*^4^-Me dT^4^ (magenta carbons, PDB ID 5DLF(41)) opposite incoming dAMPnPP. (C) Comparison between insertion complexes featuring *O*^4^-Me tT^4^ (yellow carbons, in this work) or *O*^4^-Me dT^4^ (magenta carbons, PDB ID 5DLF) opposite incoming dAMPnPP. (D) Comparison between extension complexes featuring *O*^4^-Me tT^5^ (yellow carbons, this work) or *O*^4^-Et dT^5^ (light blue, PDB ID 5DQI(41)) paired opposite primer dA^8^ at the −1 postreplicative position and wedged between the replicating dG^4^:dCMPnPP (dCTP) and −2 position dT^6^:dA^7^ base pairs. (E) Comparison between ternary complexes of hPol η with incoming dTTP (light pink carbons, PDB ID 6PL7) and tTTP (yellow carbons, this work) opposite dA^4^ in the DNA template-primer duplex. (F) Comparison between ternary complexes of hPol η with incoming rCTP opposite dG^4^ (cyan carbons, PDB ID 5EWE(57)) or tTTP opposite dA^4^ (yellow carbons, this work). Coordinated Mg^2+^ and Ca^2+^ ions are depicted as cyan and pink spheres, respectively, in all of the figure panels. (G) Active-site view illustrating differences in the orientations of 2′-deoxyribose and threose sugar rings with respect to the Phe18 (steric gate) and Tyr92 (second line of defense) hPol η residues in the structures of complexes with dA:dTMPnPP (light pink carbons, PDB ID 6PL7) or dATP:tTTP (yellow carbons, this work), respectively. (H) Active-site view illustrating differences in the orientations of ribose and threose sugar rings with respect to the Phe18 and Tyr92 in the structures of complexes with dG:rCTP/dA:rCTP (cyan carbons, PDB ID 5EWE/6PL7^57^) or dA:tTTP (yellow carbons, this work), respectively.

Comparison between the active sites in the crystal structures of ternary hPol η complexes with either template dT^4^ (PDB ID 3MR2(42)) or tT^4^ (current study) opposite incoming dAMPnPP at the replicative position shows similar geometries and stacking interactions with the adjacent dA^5^:dT^8^ pair (Figure 6A). The shorter TNA backbone compared to DNA and the pseudotransdiaxial orientation of the 3′- and 2′-oxygen atoms of the threose sugar in the 3′ → 2′ connected TNA residue compared to standard 5′ → 3′ connected DNA results in local differences in the relative tetrose and pentose sugar orientations, respectively, as well as spacings of adjacent phosphate groups and the directions of glycosidic bonds. Another difference concerns the preceding dT^3^ template nucleotide that stacks on tT^4^ in the complex with the TNA-modified template but adopts an unstacked orientation relative to the replicating base pair in the complex with an all-DNA template-primer duplex (Figure 6A).

In the previously determined crystal structure of the ternary hPol η insertion stage complex with template *O*^4^-Me dT^4^ opposite incoming dAMPnPP (PDB ID 5DLF(41)), the base of the adducted nucleotide is lodged in a pocket to the side of the active site and thus not pairing with adenine (Figure 6B). This situation differs drastically from that in the insertion complex with an intact dT^4^:dAMPnPP pair at the replicative position (PDB ID 3MR2(42)) where that base pair also fully stacks on the adjacent dA^5^:dT^8^ pair. As discussed above, unpaired dT^3^ in that complex is unstacked from the replicating base pair. However, in the structure of the complex with template residue *O*^4^-Me dT^4^, dT^3^ lies completely outside the active site because of the adducted base being inserted into a pocket adjacent to the active site (Figure 6B).

The overlay of the active sites in the ternary hPol η insertion stage complexes with the adducted *O*^4^-Me thymine either attached to a template TNA or DNA sugar–phosphate backbone reveals completely different orientations of the *O*^4^-Me tT^4^ (current work) and *O*^4^-Me dT^4^ residues (PDB ID 5DLF(41)) (Figure 6C). Neither pairs with the incoming dAMPnPP nor do they stack on the adjacent dA^5^:dT^8^ pair, but only the adducted *O*^4^-Me tT^4^ assumes a position inside the helix at the active site. By comparison, the adducted *O*^4^-Me dT^4^ base moiety points toward the ceiling of the active site and is sequestered inside a side pocket (Figure 6C). These differences could arise from the inability of the TNA residue to mimic the orientation of the DNA backbone presumably because of the uniform C4′-*exo* pucker of the tetrose sugar and different relative orientations of the glycosidic bonds in the TNA and DNA frameworks. Also, with the tT-modified template, the preceding orphaned dT^3^ nucleotide stacks on tT^4^ of the tT:dAMPnPP pair (Figure 6A). Thus, with the TNA-modified template, both the adducted *O*^4^-Me tT^4^ and dT^3^ assume intrahelical orientations as opposed to the DNA template in the corresponding structure where both *O*^4^-Me dT^4^ and dT^3^ are unstacked (Figure 6B,C).

In the extension state ternary hPol η complex, TNA *O*^4^-Me tT^5^ does not base pair with the terminal primer residue dA^8^ (Figure 5D). The *syn* orientation of the *O*^4^-methyl group would likely cause a steric clash with adenine if the base of the terminal primer residue adopted a regular stacked arrangement. Instead, dA^8^ avoids a clash and recedes deep inside the bottom of the active site, whereby the poorly defined electron density is indicative of an inherently flexible behavior. Consequently, the extension complex with *O*^4^-Me tT does not display a productive conformation for successful nucleotidyl transfer for the addition of incoming dCMPnPP opposite template dG^4^. Full-length primer extension with *O*^4^-Me tT-containing DNA, as observed in the biochemical assays (Figure 3A), may result from dNTP misincorporation. The void opposite *O*^4^-Me tT^5^ is filled with a glycerol molecule, and the template bases dT^3^, dG^4^, *O*^4^-Me tT^5^, and dT^6^ form a continuous stack (Figures 5D and 6D). Conversely, the *O*^4^-Et moiety of *O*^4^-Et dT^5^ in the extension complex with the adducted template pointed into the major groove, thereby allowing H-bond formation between the *N*^6^ amino group of primer dA^8^ and *N*^3^ and *O*^4^ of the adducted base (Figure 6D) (PDB ID 5DQI(41)). Apart from modified base pairs, in both complexes with *O*^4–^Me tT and *O*^4^-Et dT-containing DNA, the adjacent residues of the template and primer strands remain fully stacked. The unadducted tT or dT base pair and stack opposite incoming nucleotide dAMPnPP are consistent with greater dAMP incorporation relative to *O*^4^-Me dT/tT and O^4^-Et tT/dT (Figure 6A).

Although both DNA and TNA *O*^4^-alkylated template residues can be bypassed by hPol η, crystal structures of ternary complexes with the adducted residues in the replicative and postreplicative positions reveal some interesting differences between the DNA and TNA backbones of adducted nucleotides at the pol active site. Among them are the deviating orientations of the *O*^4^-Me tT^4^ and *O*^4^-Me dT^4^ residues in hPol η insertion stage complexes (Figure 6B,C), where the latter residue is pulled out of the active site and accommodated in an adjacent pocket, and different orientations of the methyl and ethyl substituents of *O*^4^-Me tT^5^ and *O*^4^-Et dT^5^, respectively. Moreover, in hPol η extension stage complexes, the former residue forces the terminal primer residue out of the active site (Figure 6D), whereas in the latter, the terminal primer base pairs with the adducted residue. Currently, we lack experimental structure-based evidence for *O*^4^-Et tT. The nucleotide incorporation efficiency was further reduced compared with the adduct in combination with a DNA backbone. This may be attributed to a possible steric clash between O^4^-Et tT and the incoming nucleotide that leads to unstacking of the adducted base.

hPol η can replicate across tT or adducted tT-containing DNA to synthesize DNA; but surprisingly, it does not incorporate a TNA nucleotide (tTTP) opposite native DNA in the template strand (Figure 3B). Because of a 3′ → 2′ phosphodiester linkage in tTTP versus a 5′ → 3′ linkage in dTTP, the altered tetrose orientation combined with a C4′-*exo* pucker, the Pα of the incoming tTTP is too far removed from the primer’s terminal 3′-OH for a nucleophilic attack to occur (Figure 4E,F). This is unlike the situation with dTTP (PDB ID 6PL7) (Figure 6E) or dNMPnPPs bound in a productive manner opposite tT inside DNA (Figure 4A–D) where Pα lies in close proximity to the terminal 3′-OH for successful nucleotidyl transfer. Perhaps engineered hPol η or modified TNA nucleotide substrates may result in successful nucleotidyl transfer. In previous work, DNA polymerases from archaeal sources such as Kod RI or Therminator DNA polymerase were engineered to utilize TNA trinucleotide triphosphates as substrate for TNA synthesis.^34,38^ Kod RI (A485R and E664I) exhibited 5-fold faster primer extension efficiency and ∼20-fold higher fidelity than engineered Therminator DNA polymerase (A485L).^34,38^ X-ray crystal structures revealed a cavity at the active site of laboratory-evolved polymerase Kod-RSGA that accommodates the substituent of C5-modified tUTP substrate for facilitating TNA synthesis.^35^

Besides 2′-deoxyribonucleotide triphosphates (dNTPs), hPol η can insert ribonucleotide triphosphates (rNTPs) opposite a standard or adducted DNA template base, reverse-transcribe RNA to DNA, and also act as an RNA pol.^57,58,65^ However, as we demonstrate here, hPol η is unable to incorporate a TNA nucleotide into a DNA primer opposite a DNA template. Su et al. determined the structure of the ternary complex of hPol η with incoming rCTP opposite dG (PDB ID 5EWE(57)). An overlay of that complex and the ternary complex with tTTP opposite dA (current work) clearly shows the proximity of the primer’s terminal 3′-OH to Pα of the incoming nucleotide in the former and differences in the sugar puckers of rCTP and tTTP (Figure 6F).

Additionally, there are differences in the orientations of the ribose (dG:rCTP; PDB ID 5EWE(57)), 2′-deoxyribose (dA:dTTP; PDB ID 6PLV) and threose (dA:tTTP; current study) sugar ring orientations with respect to protein residue Phe18 (Figure 6G,H). This phenylalanine acts as a steric gate adjacent to the active site to discriminate between incoming rCTP/dTTP^42,57,58^ and tTTP. Tyr92 acts as a second line of defense to stabilize Phe18 through π–π interactions. The 2′-OH of the incoming rCTP avoids a direct clash with the phenyl ring of the steric gate residue and shifts relative to the position of dTTP in the dA:dTTP complex structure (Figure 6G,H). Consequently, a significant propeller twist occurs between cytosine of the incoming nucleotide (rCTP) and guanine of the template, and the 1 Å distance increase between Pα and the primer terminal 3′-OH ultimately leads to reduced incorporation compared to incoming dTTP. To avoid a steric clash between the 2′-OH group of tTTP and the Phe18 ring, the nucleoside moiety is shifted further relative to rCTP, ultimately precluding a productive reaction for tTMP insertion into the DNA primer (Figure 6H).

In conclusion, the biochemical and structural data presented in this study suggest that hPol η functions as a TNA-directed DNA pol, thus contributing to ongoing efforts to screen TNA-compatible enzymes. It will be interesting to investigate further how accurate and efficient DNA polymerization mediated by this specialized Y-family DNA pol is compared to other known TNA-compatible DNA pols and opposite other XNAs in a DNA template.

## Data Availability

Atomic coordinates and structure factors for the X-ray crystal structures presented in this study have been deposited with the Protein Data Bank under PDB IDs 9CHW, tT (insertion); 9CI9, tT (extension); 9CJ9, *O*^4^-Me tT (insertion); 9CIH, *O*^4^-Me tT (extension); 9CIQ, dA:tTTP. Corresponding raw diffraction data sets have been deposited with SB Grid under accession codes 1124, 1125, 1126, 1127, and 1128, respectively.

## Supporting Information Available

Reagents and general methods for chemical synthesis of TNA T phosphoramidites; synthesis and purification of oligonucleotides; reaction scheme S1 showing synthesis of TNA T phosphoramidites; ^1^H, ^13^C, and ^31^P NMR spectra of compounds shown in reaction scheme S1; SAX-HPLC chromatograms of purified oligonucleotides; electrospray ionization mass spectrometry (ESI-MS) spectra of purified unmodified oligonucleotides; SAX-HPLC chromatograms of purified oligonucleotides; ESI-MS spectra of purified tT, *O*^4^-Me tT, and *O*^4^-Et tT (PDF)

## Abbreviations

TNA: α-l-(3′-2′)-threofuranosyl nucleic acid
RNA: ribonucleic acid
RP-HPLC: reverse-phase high performance liquid chromatography
