# Supplementary material for: DNA Replication across α-l-(3′-2′)-Threofuranosyl Nucleotides Mediated by Human DNA Polymerase η
Source: Biochemistry. 2024 Sep 11;63(19):2425–39. doi: 10.1021/acs.biochem.4c00387 (PMC11447838; doi:10.1021/acs.biochem.4c00387)
Supplement: Supplementary file 1 — bi4c00387_si_001.pdf [file bi4c00387_si_001.pdf]

Supporting Information

DNA Replication Across α-L-(3′-2′)-Threofuranosyl Nucleotides Mediated by Human DNA Polymerase *η*

Rachana Tomar[1], Pratibha P. Ghodke[2], Amritraj Patra[2], Elizabeth Smyth[3], Alexander Pontarelli[3], William Copp[3], F. Peter Guengerich[2], John C. Chaput[4], Christopher J. Wilds[3], Michael P. Stone[1,*] and Martin Egli[2,*]

[1]Department of Chemistry, Vanderbilt Ingram Cancer Center, and Vanderbilt Center for Structural Biology, Vanderbilt University, Nashville, TN 37235, USA

[2]Department of Biochemistry, School of Medicine, Vanderbilt Ingram Cancer Center, and Vanderbilt Center for Structural Biology, Vanderbilt University, Nashville, TN 37232, USA

[3]Department of Chemistry and Biochemistry, Concordia University, Montréal, Québec H4B 1R6, Canada

[4]Department of Pharmaceutical Sciences, University of California, Irvine, CA 92697, USA

*Correspondence: martin.egli@vanderbilt.edu; michael.p.stone@vanderbilt.edu

Contents **Page**

Experimental Procedures

Reagents and general methods S-7

Chemical synthesis of nucleosides S-8

Synthesis and purification of oligonucleotides S-15

Supporting Schemes

Supporting Scheme 1: Synthesis of TNA T phosphoramidites **5a** and **5b** S-17

Supporting Figures

Supporting Figure 1: 500 MHz ^{1}H NMR spectrum of compound **2** in $CDCl_3$ S-19

Supporting Figure 2: 125.7 MHz ^{13}C NMR spectrum of compound **2** in $CDCl_3$ S-20

Supporting Figure 3: 500 MHz ^{1}H NMR spectrum of compound **3a** in $CDCl_3$ S-21

Supporting Figure 4: 125.7 MHz ^{13}C NMR spectrum of compound **3a** in $CDCl_3$ S-22

Supporting Figure 5: 500 MHz ^{1}H NMR spectrum of compound **3b** in $CDCl_3$ S-23

Supporting Figure 6: 125.7 MHz ^{13}C NMR spectrum of compound **3b** in $CDCl_3$ S-24

Supporting Figure 7: 500 MHz ^{1}H NMR spectrum of compound **4a** in $CDCl_3$ S-25

Supporting Figure 8: 125.7 MHz ^{13}C NMR spectrum of compound **4a** in $CDCl_3$ S-25

Supporting Figure 9: 500 MHz ^{1}H NMR spectrum of compound **4b** in $CDCl_3$ S-27

Supporting Figure 10: 125.7 MHz ^{13}C NMR spectrum of compound **4b** in $CDCl_3$ S-28

Supporting Figure 11: 500 MHz ^{1}H NMR spectrum of compound **5a** in d_6-acetone S-29

Supporting Figure 12: 125.7 MHz ^{13}C NMR spectrum of compound **5a** in d_6-acetone S-30

Supporting Figure 13: 202.3 MHz ^{31}P NMR spectrum of compound **5a** in d_6-acetone S-31

Supporting Figure 14: 500 MHz ^{1}H NMR spectrum of compound **5b** in d_6-acetone S-32

Supporting Figure 15: 125.7 MHz ^{13}C NMR spectrum of compound **5b** in d_6-acetone S-33

Supporting Figure 16: 202.3 MHz ^{31}P NMR spectrum of compound **5b** in d_6-acetone S-34

Supporting Figure 17: SAX-HPLC chromatograph of purified 5'-AGC GTC AT-3' S-35

Supporting Figure 18: ESI MS spectrum of purified 5'-AGC GTC AT-3' S-35

Supporting Figure 19: SAX-HPLC chromatograph of purified 5'-AGC GTC AA-3' S-36

Supporting Figure 20: ESI MS spectrum of purified 5'-AGC GTC AA-3' S-36

**Supporting Figure 21:**	SAX-HPLC chromatograph of purified 5'-TCA TTA TGA CGC TTA CGA-3'	S-37
**Supporting Figure 22:**	ESI MS spectrum of purified 5'-TCA TTA TGA CGC TTA CGA-3'	S-37
**Supporting Figure 23:**	SAX-HPLC chromatograph of purified 5'-TCA TXA TGA CGC TTA CGA-3' (X = tT)	S-38
**Supporting Figure 24:**	ESI MS spectrum of purified 5'-TCA TXA TGA CGC TTA CGA- 3' (X = tT)	S-38
**Supporting Figure 25:**	SAX-HPLC chromatograph of purified 5'-CAT XAT GAC GCT-3' (X = tT)	S-39
**Supporting Figure 26:**	ESI MS spectrum of purified 5'-CAT XAT GAC GCT-3' (X = tT)	S-39
**Supporting Figure 27:**	SAX-HPLC chromatograph of purified 5'-CAT GXT GAC GCT-3' (X = tT)	S-40
**Supporting Figure 28:**	ESI MS spectrum of purified 5'-CAT GXT GAC GCT-3' (X = tT)	S-40
**Supporting Figure 29:**	SAX-HPLC chromatograph of purified 5'-CAT XAT GAC GCT-3' (X = O^4-Me tT)	S-41
**Supporting Figure 30:**	ESI MS spectrum of purified 5'-CAT XAT GAC GCT-3' (X = O^4-Me tT)	S-41

Supporting Figure 31: SAX-HPLC chromatograph of purified 5'-CAT XAT GAC GCT-3' (X = O^4-Et tT) S-42

Supporting Figure 32: ESI MS spectrum of purified 5'-CAT XAT GAC GCT-3' (X = O^4-Et tT) S-42

Supporting Figure 33: SAX-HPLC chromatograph of purified 5'-CAT GXT GAC GCT-3' (X = O^4-Me tT) S-43

Supporting Figure 34: ESI MS spectrum of purified 5'-CAT GXT GAC GCT-3' (X = O^4-Me tT) S-43

Supporting Figure 35: SAX-HPLC chromatograph of purified 5'-CAT GXT GAC GCT-3' (X = O^4-Et tT) S-44

Supporting Figure 36: ESI MS spectrum of purified 5'-CAT GXT GAC GCT-3' (X = O^4-Et tT) S-44

Supporting Figure 37: SAX-HPLC chromatograph of purified 5'-TCA TXA TGA CGC TTA CGA-3' (X = O^4-Me dT) S-45

Supporting Figure 38: ESI MS spectrum of purified 5'-TCA TXA TGA CGC TTA CGA-3' (X = O^4-Me dT) S-45

Supporting Figure 39: SAX-HPLC chromatograph of purified 5'-TCA TXA TGA CGC TTA CGA-3' (X = O^4-Et dT) S-46

Supporting Figure 40: ESI MS spectrum of purified 5'-TCA TXA TGA CGC TTA CGA-3' (X = O^4-Et dT) S-46

Supporting Figure 41: SAX -HPLC chromatograph of purified 5'-TCA TXA TGA CGC TTA CGA-3' (X = O^4-Me tT) S-47

Supporting Figure 42: ESI MS spectrum of purified 5'-TCA TXA TGA CGC TTA CGA-3' (X = O^4-Me tT) S-47

Supporting Figure 43: SAX-HPLC chromatograph of purified 5'-TCA TXA TGA CGC TTA CGA-3' (X = O^4-Et tT) S-48

Supporting Figure 44: ESI MS spectrum of purified 5'-TCA TXA TGA CGC TTA CGA-3' (X = O^4-Et tT) S-48

References S-49

Experimental Procedures

Reagents and general methods

N,N-Diisopropylamino cyanoethyl phosphonamidic chloride was purchased from ChemGenes Corporation (Wilmington, MA). All other chemicals and solvents were purchased from MilliporeSigma (St. Louis, MO) or Thermo Fisher Scientific (Waltham, MA). Acetonitrile (ACN), dichloromethane (DCM), ethyl acetate (EtOAc), hexanes, methanol (MeOH), and tetrahydrofuran (THF) were used as purchased. Deuterated chloroform ($CDCl_3$) and deuterated acetone (d_6-acetone) were purchased from ACP Chemicals Inc. (Montréal, QC). Flash column chromatography was performed using silica gel 60 (230-400 mesh) purchased from Canadian Life Science (Pointe-Claire, QC). Thin layer chromatography (TLC) was carried out with pre-coated TLC plates (Merck, Kieselgel 60 F_{254}, 0.25 mm) purchased from EMD Chemicals Inc. (Gibbstown, NJ). NMR spectra were acquired on a Varian 500 MHz NMR spectrometer at room temperature. ^{1}H NMR spectra were recorded at a frequency of 500.0 MHz and chemical shifts reported in parts per million (ppm) downfield from tetramethylsilane. ^{13}C NMR spectra (^{1}H decoupled) were recorded at a frequency of 125.7 MHz and chemical shifts were reported in ppm with tetramethylsilane as a reference. ^{31}P NMR spectra (^{1}H decoupled) were recorded at a frequency of 202.3 MHz and chemical shifts were reported in ppm with phosphoric acid used as an external standard. Mass spectrometry analyses of nucleoside derivatives was performed at the Concordia University Centre for Biological Applications of Mass Spectrometry (CBAMS) on a Thermo LTQ Orbitrap Velos mass spectrometer equipped with a heated electrospray ion source in positive mode.

Chemical synthesis of nucleosides

1-[2'-*O*-*tert*-Butyldimethylsilyl-3'-*O*-4,4'-dimethoxytrityl-α-L-threofuranosyl] thymine (**2**)

1-[3'-*O*-4,4'-Dimethoxytrityl-α-L-threofuranosyl] thymine (**1**) was previously synthesized following a literature protocol [1]. To a solution of **1** (2.20 g, 4.15 mmol) dissolved in anhydrous DCM (45.3 mL), was added 4-dimethylaminopyridine (40.2 mg, 0.33 mmol) and imidazole (2.04 g, 29.9 mmol). *tert*-Butyldimethylsilyl chloride (3.47 g, 23.0 mmol) was added last, and the reaction mixture was stirred for 40 h at room temperature. The following crude suspension mixture was concentrated, dissolved in DCM (100 mL), sequentially washed with H_2O (100 mL) and 3% sodium bicarbonate solution (100 mL), and dried over anhydrous sodium sulfate. The solvent was removed in vacuo, and the crude product was purified by silica gel flash column chromatography using hexanes/EtOAc (8:2) as eluent to give **2** as a colorless foam (2.08 g, 3.23 mmol, 78 %). R_f (SiO_2 TLC): 0.17 (hexanes/EtOAc (8:2)). λ_{max} (ACN) = 271, 237 nm. ^1H NMR (500 MHz, $CDCl_3$, ppm): δ 8.69 (s, 1H, NH), 7.39 (s, 1H, H6), 7.19-7.33 (m, 9H, Ar), 6.81-6.83 (m, 4H, Ar), 5.58 (bs, 1H, H1'), 4.20 (s, 1H, H2'), 4.03 (d, 1H, H4'), 3.81-3.82 (d, 1H, H4'), 3.79 (s, 6H, 2 × OCH_3), 3.20-3.22 (d, 1H, H3'), 1.79 (s, 3H, C5-CH_3), 0.82 (s, 9H, Si-$C(CH_3)_3$), 0.17 (s, 3H, Si-CH_3), 0.04 (s, 3H, Si-CH_3). ^{13}C NMR (125.7 MHz, $CDCl_3$, ppm): δ 164.01, 158.78, 158.76, 150.21, 144.61, 137.06, 135.78, 135.58, 129.79, 129.70, 128.16, 127.66, 127.11, 113.60, 113.53, 108.76, 92.84, 88.08, 80.69, 78.37, 75.30, 60.37, 55.25, 25.59, 17.74, 14.19, 12.70, -4.59, -5.07. HRMS (ESI-MS) *m/z* calculated for $C_{36}H_{44}N_2O_7SiNa^+$: 667.2816; found 667.2828 $[M + Na]^+$.

O^4-Methyl-1-[2'-*O*-*tert*-butyldimethylsilyl-3'-*O*-4,4'-dimethoxytrityl-α-L-threofuranosyl] thymine (**3a**)

Compound **2** was co-evaporated with 10 mL of anhydrous ACN. To a solution of **2** (0.25 g, 0.39 mmol), 1,2,4-triazole (0.60 g, 8.7 mmol), triethylamine (1.3 mL, 8.9 mmol) in ACN:DCM (3.8:3.8 mL) was added phosphorous oxychloride (89 µL, 0.97 mmol) dropwise while stirring on ice. After 3.5 h, additional 1,2,4-triazole (0.12 g, 1.7 mmol), triethylamine (250 µL, 1.8 mmol) and phosphorous oxychloride (9.1 µL, 0.098 mmol) were added dropwise while stirring on ice. After 5 h, the solvent was removed *in vacuo* and the volume was brought to 100 mL with DCM. The organic layer was washed with a 3% sodium bicarbonate solution (2 × 75 mL), brine (1 × 75 mL), dried over anhydrous sodium sulfate (~4 g), decanted and the solvent removed *in vacuo*. To a solution of the crude compound in MeOH (5 mL) was added sodium methoxide (0.073 g, 1.4 mmol). After 18 h, the solvent was removed *in vacuo* and the volume was brought to 100 mL with DCM. The organic layer was washed with a 3% sodium bicarbonate solution (2 x 75 mL), brine (1 × 75 mL), then dried over anhydrous sodium sulfate (~4 g), decanted and the solvent removed *in vacuo*. The crude compound was purified by silica gel flash column chromatography with a gradient of MeOH:DCM (0.5 → 1 %) to obtain **3a** (150 mg, 0.23 mmol, 60 % over two steps) as a colourless foam. R_f (SiO_2, TLC): 0.48 (5 % MeOH:DCM). λ_{max} (ACN) = 282 nm. ^1H NMR (500 MHz, $CDCl_3$) δ 7.48 (d, J = 0.7 Hz, 1H, H6), 7.32-7.06 (m, 9H, Ar), 6.86-6.69 (m, 4H, Ar), 5.62 (s, 1H, H1'), 4.46 (s, 1H, H3'), 4.04 (s, 3H, -O^4-CH_3), 3.99 (d, J = 3.4 Hz, 1H, H2'), 3.81 (dd, J = 9.9, 3.5 Hz, 1H, H4'), 3.78 (s, 6H, 2 x OCH_3), 3.06 (d, J = 9.8 Hz, 1H, H4'), 1.80 (d, J = 0.5 Hz, 3H, C5-CH_3), 0.82 (s, 9H, Si-$C(CH_3)_3$), 0.26 (s, 3H, Si-CH_3), 0.09 (s, 3H, Si-CH_3).

^{13}C NMR (125.7 MHz, $CDCl_3$) δ 170.92, 158.69, 158.66, 156.14, 144.70, 140.90, 135.96, 135.65, 129.79, 129.71, 128.00, 127.69, 126.96, 113.48, 113.38, 102.75, 93.82, 87.85, 80.63, 78.43, 75.35, 55.22, 55.21, 54.44, 25.67, 17.76, 12.39, -4.46, -5.23. HRMS (ESI-MS) *m/z* calculated for $C_{37}H_{46}N_2NaO_7Si^+$: 681.2972; found: 681.3050 $[M+Na]^+$.

O^4-Ethyl-1-[2'-*O*-*tert*-butyldimethylsilyl-3'-*O*-4,4'-dimethoxytrityl-α-L-threofuranosyl] thymine **(3b)**

To a solution of **2** (2.08 g, 3.23 mmol) dissolved in anhydrous ACN (40.0 mL), was added 1,2,4-triazole (5.03 g, 72.6 mmol) and triethylamine (10.4 mL) on ice. Phosphorous oxychloride (0.75 mL) was slowly added dropwise to the solution on ice. Following ten minutes on ice, the reaction mixture was removed from ice and stirred for 1.5 h at room temperature. The following crude suspension mixture was concentrated, dissolved in DCM (70 mL), washed twice with 3% sodium bicarbonate solution (2 × 70 mL), and dried over anhydrous sodium sulfate. The crude product was divided into two equal parts (2 x 1.61 mmol) with 20 mL (2 x 10 mL) of DCM before co-evaporating with ethanol (5 mL). To the crude product dissolved in anhydrous ethanol (12 mL), was added 1,8-diazabicyclo[5.4.0]undec-7-ene (603 µL, 4.04 mmol). The reaction mixture was stirred at room temperature for 50 min. The following crude suspension mixture was concentrated, dissolved in DCM (70 mL), washed sequentially with 3% sodium bicarbonate solution (70 mL) and brine (70 mL), and dried over anhydrous sodium sulfate. The solvent was removed *in vacuo*, and the crude product was purified by silica gel flash column chromatography using hexanes/EtOAc (8:2) as eluent to give **3b** a colorless foam (0.900 g, 1.34 mmol, 82 % over

two steps). R_f (SiO_2 TLC): 0.20 hexanes/EtOAc (8:2). λ_{max} (ACN) = 282 nm. ^1H NMR (500 MHz, $CDCl_3$, ppm): δ 7.48 (s, 1H, H6), 7.14-7.23 (m, 9H, Ar), 6.76-6.81 (m, 4H, Ar), 5.62 (bs, 1H, H1'), 4.50-4.54 (q, 2H, -O^4-CH_2), 4.46 (s, 1H, H2'), 3.98-3.99 (d, 1H, H4'), 3.81-3.82 (d, 1H, H4'), 3.79 (s, 6H, 2 x OCH_3), 3.05-3.07 (d, 1H, H3'), 1.80 (s, 3H, C5-CH_3), 1.41-1.43 (t, 3H, CH_3), 0.82 (s, 9H, Si-$C(CH_3)_3$), 0.26 (s, 3H, Si-CH_3), 0.10 (s, 3H, Si-CH_3). ^{13}C NMR (125.7 MHz, $CDCl_3$, ppm): δ170.56, 158.68, 158.64, 156.22, 144.69, 140.78, 136.00, 135.68, 129.80, 129.70, 128.00, 127.71, 126.96, 113.47, 113.38, 102.89, 93.81, 87.83, 80.62, 78.44, 75.34, 63.01, 55.23, 25.68, 17.76, 14.41, 12.43, -4.46, -5.23. HRMS (ESI-MS) *m/z* calculated for $C_{38}H_{48}N_2O_7SiNa^+$ 695.3129: found 695.3160 $[M + Na]^+$.

O[4]-Methyl-1-[3'-*O*-4,4'-dimethoxytrityl-α-L-threofuranosyl] thymine (**4a**)

To a solution of **3a** (0.15 g, 0.22 mmol) in THF (3 mL) was added tetra-n-butylammonium fluoride (1 M in THF) (260 µL, 0.26 mmol) dropwise while stirring. After 5 min, the solvent was removed *in vacuo* and the volume was brought to 100 mL with DCM. The organic layer was washed with 3% sodium bicarbonate solution (2 x 75 mL), brine (1 x 75 mL), dried over anhydrous sodium sulfate (~4 g), decanted and the solvent removed *in vacuo*. The crude compound was purified by silica gel flash column chromatography with (5 % MeOH:DCM) to obtain **4a** as a colourless foam (100 mg, 0.18 mmol, 84 %). R_f (SiO_2, TLC): 0.30 (5 % MeOH:DCM). λ_{max} (ACN) = 282 nm. ^1H NMR (500 MHz, $CDCl_3$) δ 7.55 (s, 1H, H6), 7.44-7.05 (m, 9H, Ar), 6.91-6.66 (m, 4H, Ar), 5.61 (d, J = 1.1 Hz, 1H, H1'), 4.25-4.19 (m, 2H, H2' + H3'), 4.02 (s, 3H, O^4-CH_3), 3.78 (s, 6H, 2 × OCH_3), 3.54 (dd, J = 9.8, 4.4 Hz, 1H, H4'), 3.25 (dd, J =

9.8, 2.6 Hz, 1H, H4'), 1.89 (s, 3H, C5-CH_3). ^{13}C NMR (125.7 MHz, $CDCl_3$) δ 171.10, 158.74, 158.72, 156.68, 144.80, 139.80, 136.06, 135.78, 129.92, 129.88, 127.99, 127.87, 127.00, 113.44, 113.38, 103.80, 93.84, 87.63, 81.44, 77.79, 74.28, 55.23, 54.67, 46.04, 12.36, -0.02. HRMS (ESI-MS) *m/z* calculated for $C_{31}H_{32}N_2NaO_7^+$: 567.2107; found: 567.2189 $[M+Na]^+$.

O[4]-Ethyl-1-[3'-*O*-4,4'-dimethoxytrityl-α-L-threofuranosyl] thymine (**4b**)

To a solution of **3b** (839 mg, 1.25 mmol) dissolved in THF (17.3 mL) was added tetra-n-butylammonium fluoride (1 M in THF) (3.12 mL, 3.12 mmol). The reaction mixture was stirred for 20 min at room temperature. The following crude suspension mixture was concentrated, dissolved in DCM (60 mL), washed sequentially with deionized water (60 mL) and 3% sodium bicarbonate solution (60 mL), and dried over anhydrous sodium sulfate. The solvent was removed *in vacuo*, and the crude product was purified by silica gel flash column chromatography using hexanes/EtOAc (1:1) to give **4b** as a colorless foam (0.67 g, 1.20 mmol, 96 %). R_f (SiO_2 TLC): 0.13 (hexanes/EtOAc (1:1)). λ_{max} (ACN) = 282 nm. ^{1}H NMR (500 MHz, $CDCl_3$, ppm): δ 7.55 (s, 1H, H6), 7.19-7.37 (m, 9H, Ar), 6.80-6.83 (m, 4H, Ar), 5.57 (bs, 1H, H1'), 4.44-4.45 (m, 2H, O^4-CH_2), 4.21-4.23 (s, 1H, H3'), 4.17 (bs, 1H, H2'), 3.98 (d, 1H, OH), 3.79 (s, 6H, 2 x OCH_3), 3.49-3.52 (dd, 1H, H4'), 3.26-3.28 (dd, 1H, H4'), 1.90 (s, 3H, C5-CH_3), 1.39-1.42 (t, 3H, CH_3). ^{13}C NMR (125.7 MHz, $CDCl_3$, ppm): δ170.76, 158.72, 156.79, 144.81, 139.55, 136.07, 135.79, 129.93, 129.90, 128.00, 127.89, 127.00, 113.43, 113.37, 103.99, 93.81, 87.59, 81.59, 77.77, 74.15, 63.36, 55.23, 14.29, 12.39, -0.02. HRMS (ESI-MS) *m/z* calculated for $C_{32}H_{34}N_2O_7Na^+$: 581.2264; found: 581.2298 $[M + Na]^+$.

O^4-Methyl-1-[2'-O-2-cyanoethoxy(diisopropylamino)-phosphino-3'-O-4,4'-dimethoxytrityl-α-L-threofuranosyl] thymine (**5a**)

To a solution of **4a** (95 mg, 0.17 mmol), *N*,*N*-diisopropylethylamine (48 μL, 0.28 mmol) in DCM (2 mL) was added N,N-diisopropylamino cyanoethyl phosphonamidic chloride (52 μL, 0.23 mmol) while stirring. After 30 min, the solvent was removed *in vacuo* and the crude compound was dissolved in 50 mL of EtOAc. The compound was washed with 3% sodium bicarbonate solution (2 x 35 mL), brine (35 mL) and dried over anhydrous sodium sulfate (~2 g). The crude compound was purified by silica gel flash column chromatography (conditioned with 0.5 % triethylamine/EtOAc) with EtOAc:hexanes (8:2) to obtain **5a** as a colourless foam (107 mg, 0.144 mmol, 84 %). R_f (SiO_2, TLC): 0.62, 0.71 (100 % EtOAc). λ_{max} (ACN) = 282 nm. ^1H NMR (500 MHz, d_6-acetone) δ 7.63-7.71 (m, 1H, H6), 6.84-7.39 (m, 13H, Ar), 5.74-5.80 (m, 1H, H1'), 4.17-4.32 (m, 1H, H3'), 3.94-3.98 (m, 3H, O^4-CH_3), 3.29-4.08 (m, 14H, NCH, $ArOCH_3$, CH_2OP, H4', CH_2CN), 1.84 (s, 3H, C5-CH_3), 1.00-1.25 (m, 12H, 4 x CH$\underline{CH_3}$). ^{13}C NMR (125.7 MHz, d_6-acetone) δ = 170.69, 158.94, 158.93, 155.02, 154.91, 145.13, 145.10, 141.23, 141.10, 135.84, 135.70, 135.66, 129.98, 129.91, 129.86, 129.83, 127.98, 127.94, 127.79, 127.69, 126.84, 126.81, 118.28, 117.93, 113.53, 113.46, 113.43, 113.35, 102.06, 102.03, 92.51, 92.45, 92.42, 92.39, 88.03, 87.96, 81.87, 81.70, 81.35, 81.22, 77.71, 77.67, 77.40, 75.06, 74.91, 59.10, 58.94, 58.72, 58.56, 54.69, 53.55, 53.53, 43.49, 43.38, 43.20, 43.10, 24.04, 23.99, 23.93, 23.86, 23.81, 23.75, 19.74, 19.68, 19.55, 19.49, 11.65. ^{31}P NMR (202 MHz, d_6-acetone, ppm): δ

151.69, 149.97. λ_{max} (ACN) = 282 nm. HRMS (ESI-MS) *m/z* calculated for $C_{40}H_{49}N_4O_8PNa^+$: 767.3186: found 767.3199 $[M + Na]^+$.

O^4-Ethyl-1-[2'-*O*-(2-cyanoethoxy(diisopropylamino)-phosphino)-3'-*O*-4,4'-dimethoxytrityl-α-L-threofuranosyl] thymine (**5b**)

To a solution of **4b** (201 mg, 0.36 mmol) dissolved in THF (3.46 mL), was added N,N-diisopropylethylamine (0.16 mL, 0.93 mmol) followed by the dropwise addition of N,N-diisopropylamino cyanoethyl phosphonamidic chloride (158.5 µL, 0.72 mmol). The reaction mixture was stirred for 1 hour and 25 min at room temperature. The following crude suspension mixture was concentrated, dissolved in 2% triethylamine in EtOAc (60 mL), washed twice 3% sodium bicarbonate solution (2 x 40 mL), and dried over anhydrous sodium sulfate. The solvent was removed in vacuo, and the crude product was purified by silica gel flash column chromatography using hexanes/EtOAc (1:1) to give a colorless foam (320 mg, 0.438 mmol, 78 %). R_f (SiO_2 TLC): 0.32, 0.27 hexanes/EtOAc (4:6). λ_{max}(ACN) = 282 nm. ^1H NMR (500 MHz, d_6-acetone, ppm): δ 7.62-7.70 (m, 1H, H6), 6.83-7.40 (m, 13H, Ar), 5.73-5.80 (m, 1H, H1'), 4.36-4.60 (m, 3H, H3', O^4-CH_2), 4.16-4.32 (m, 1H, H2'), 3.28-4.08 (m, 14H, NCH, $ArOCH_3$, CH_2OP, H4', CH_2CN), 1.84 (s, 3H, C5-CH_3), 1.27-1.33 (t, 3H, CH_3), 1.00-1.25 (m, 12H, 4 x CH$\underline{CH_3}$). ^{13}C NMR (125.7 MHz, d_6-acetone) δ = 170.30, 158.95, 158.93, 155.05, 154.94, 145.14, 145.10, 141.17, 141.05, 135.88, 135.73, 135.70, 130.00, 129.91, 129.86, 129.85, 127.99, 127.94, 127.83, 127.72, 126.84, 126.82, 117.92, 113.53, 113.45, 113.43, 113.35, 102.16, 102.12, 92.50, 92.44, 88.03, 87.96, 81.90, 81.73, 81.38, 81.25, 77.72, 77.68, 77.41, 75.06, 74.90, 62.37,

62.35, 59.11, 58.95, 58.72, 58.56, 54.69, 43.50, 43.39, 43.21, 43.11, 24.04, 23.98, 23.92, 23.86, 23.80, 23.75, 21.89, 20.40, 19.74, 19.68, 19.55, 19.49, 13.74, 11.66. ^{31}P NMR (202 MHz, d_6-acetone, ppm): δ 151.71, 149.98. HRMS (ESI-MS) *m/z* calculated for $C_{41}H_{51}N_4O_8PNa^+$: 781.3342; found 781.3387 $[M + Na]^+$.

Synthesis and purification of oligonucleotides

Solid-phase synthesis of the oligonucleotides was performed with an Applied Biosystems Model 3400 DNA Synthesizer on a 2 μmol scale employing a β-cyanoethyl phosphoramidite cycle supplied by the manufacturer with modification to the coupling and detritylation times and the capping step as described below. Protected 5'-O-4,4'-dimethoxytrityl-2'-deoxyribonucleoside 3'-*O*-succinyl long-chain alkylamine (LCAA) controlled pore glass (CPG) supports (500 Å) and "fast deprotecting" 5'-*O*-4,4'-dimethoxytrityl-2'-deoxyribonucleoside-3'-*O*-(β-cyanoethyl-*N*,*N*'-diisopropyl) phosphoramidites were purchased from ChemGenes Inc. (Wilmington, MA). Ancillary reagents used for oligonucleotide synthesis were purchased from Glen Research (Sterling, VA). Oligonucleotide assembly began with detritylation (3% trichloroacetic acid in DCM, v/v) for 85 sec, followed by phosphoramidite coupling for both the "fast deprotecting" 2'-deoxyribonucleoside and TNA phosphoramidites (including **5a** and **5b**) dissolved to a concentration of 0.15 M in anhydrous ACN with 0.25M 5-ethylthio-1H-tetrazole in anhydrous ACN for 600 sec. Capping with phenoxyacetic anhydride-pyridine-THF (1:1:8; solution A) and 1-methyl-1H-imidazole-THF (16:84 w/v; solution B) then oxidation (0.02 M iodine in THF-water-pyridine, 2.5:2:1, v/v/v) followed every coupling. The terminal 5'-O-4,4'-dimethoxytrityl

group on the support bound oligonucleotide was removed on the synthesizer. The oligonucleotide bound CPG supports were transferred to 1.5 mL microfuge tubes fitted with teflon lined screw caps. Cleavage of the oligonucleotides from the solid support and removal of the protecting groups was performed as follows: Oligonucleotides without modified nucleobases (DNA and TNA) were treated with a solution of ammonium hydroxide (28 % in H_2O) in ethanol (3:1) at 55 ºC. Oligonucleotides (DNA and TNA) with an O^4-methyl or an O^4-ethyl adduct were treated with a 0.05 M solution of potassium carbonate in MeOH at room temperature or a 10% (v/v) solution of 1,8-diazabicyclo [5.4.0] undec-7-ene in ethanol at 55 ºC respectively. Regardless of deprotection conditions, all sequences were treated for 16 hours. The crude oligonucleotides sequences were purified by strong anion-exchange (SAX) HPLC using a Dionex DNAPac™ PA-100 column (0.4 cm × 25 cm) purchased from Dionex (Sunnyvale, CA) with a linear gradient of 0 - 52.5 % Buffer B, v/v, over 24 min (Buffer A: 100 mM Tris HCl, pH 7.5, 10% ACN and Buffer B: 100 mM Tris HCl, pH 7.5, 10% ACN, 1 M NaCl) at room temperature. The columns were monitored at 260 nm for analytical and 280 nm for preparative runs. The purified oligonucleotides were desalted using C-18 SEP PAK cartridges from Waters Corporation (Milford, MA). MS analysis of the oligonucleotides was performed at the Concordia University Centre for Biological Applications of Mass Spectrometry (CBAMS) using an Agilent 1100 LC system coupled to a Thermo LTQ Orbitrap Velos mass spectrometer equipped with a heated electrospray ion source in negative mode. MS spectra (*m/z* 300-2000) were acquired in the Orbitrap at a resolution of 60,000.

Supporting Schemes

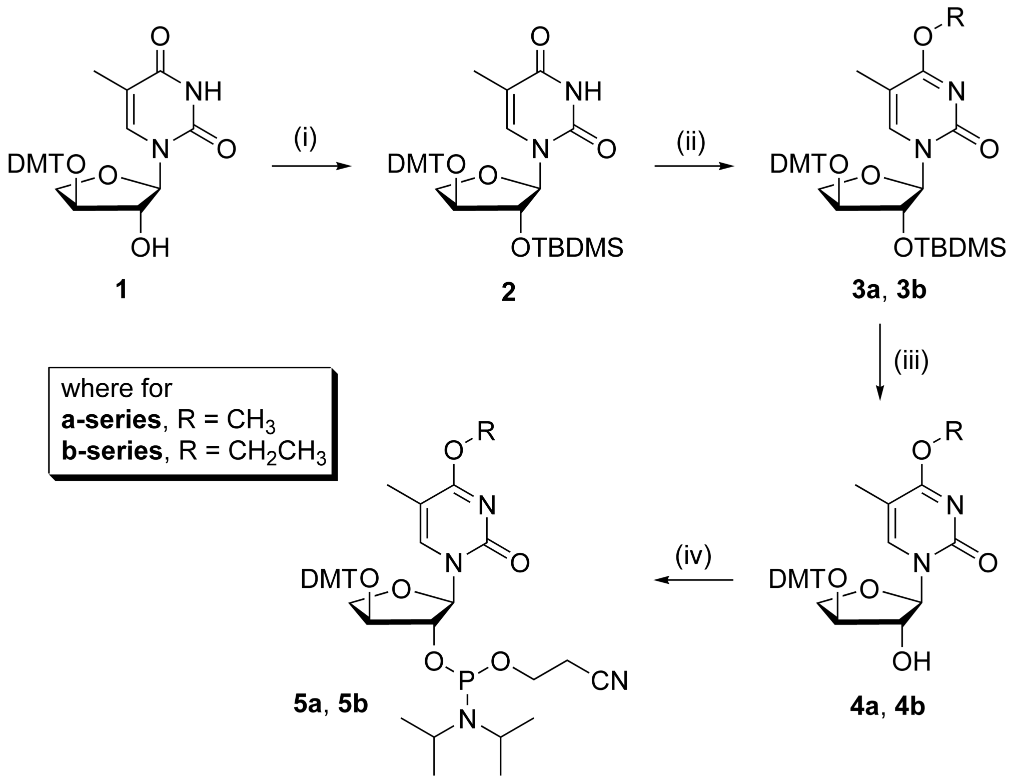

Scheme S1: Synthesis of TNA T phosphoramidites **5a** and **5b**.

Reagents and conditions: (i) *tert*-butyldimethylsilyl chloride, 4-dimethylaminopyridine, imidazole, DCM, 40 h, room temperature (78 %). **a-series** compounds: (ii) 1. 1,2,4-triazole, triethylamine, phosphorous oxychloride, ACN/DCM, 5 h, room temperature. 2. sodium methoxide, methanol, 18 h, room temperature (60 % over two steps). (iii) tetrabutylammonium fluoride (1 M in THF), THF, 25 min, room temperature (84 %). (iv) *N*,*N*-diisopropylamino cyanoethyl phosphonamidic chloride, *N*,*N*-diisopropylethylamine, DCM, 30 min, room temperature (84 %). **b-series** compounds: (ii) 1. 1,2,4-triazole, triethylamine, phosphorous

oxychloride, ACN, 1.5 h, 0ºC to room temperature. 2. 1,8-diazabicyclo[5.4.0]undec-7-ene, ethanol, 50 min, room temperature (82 % over two steps). (iii) tetrabutylammonium fluoride (1 M in THF), THF, 20 min, room temperature (96 %). (iv) *N*,*N*-diisopropylamino cyanoethyl phosphonamidic chloride, *N*,*N*-diisopropylethylamine, THF, 1.5 h, room temperature (78%).

Supporting Figure 1: 500 MHz ^{1}H NMR spectrum of compound **2** in $CDCl_3$

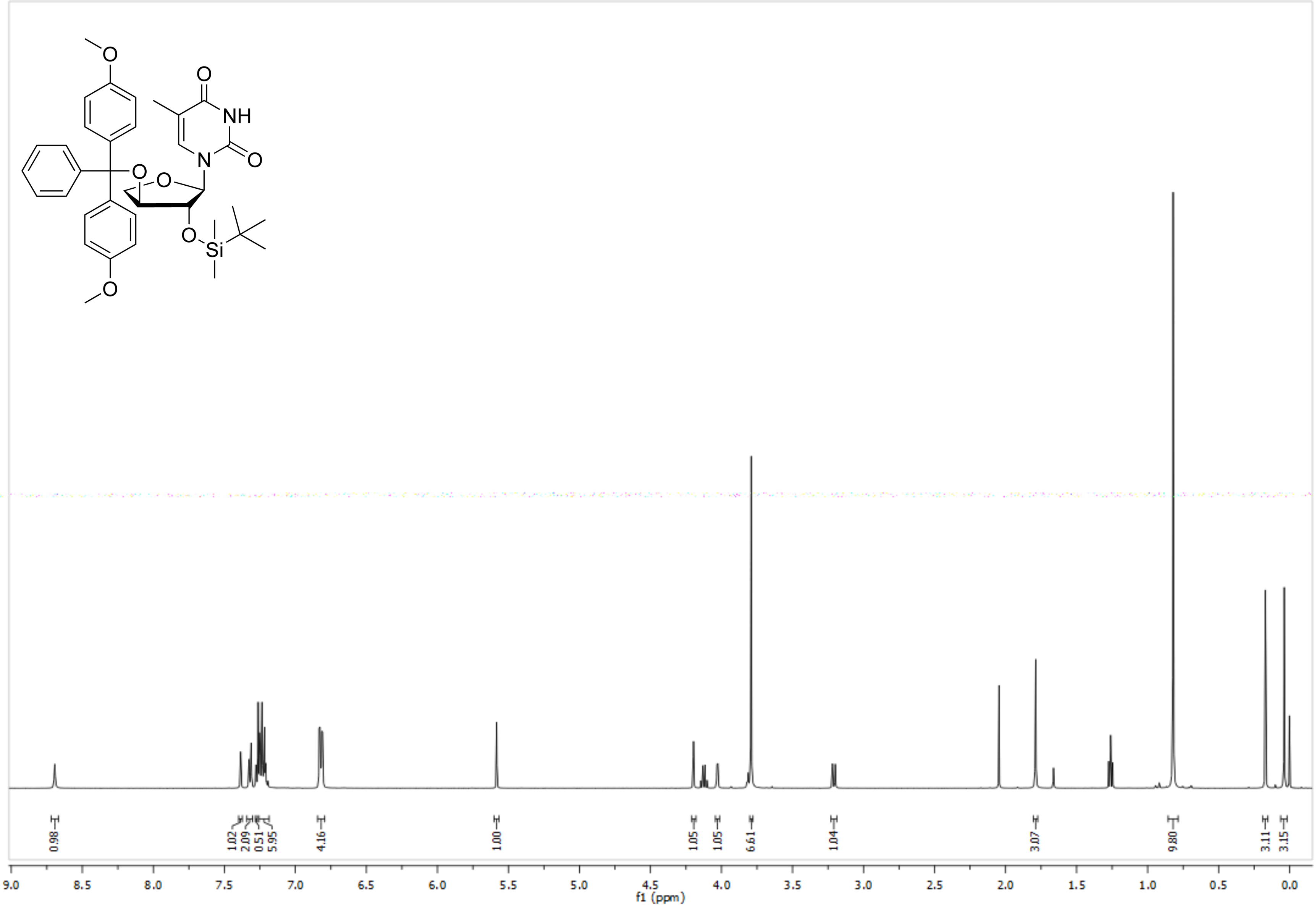

Supporting Figure 2: 125.7 MHz ^{13}C NMR spectrum of compound **2** in $CDCl_3$

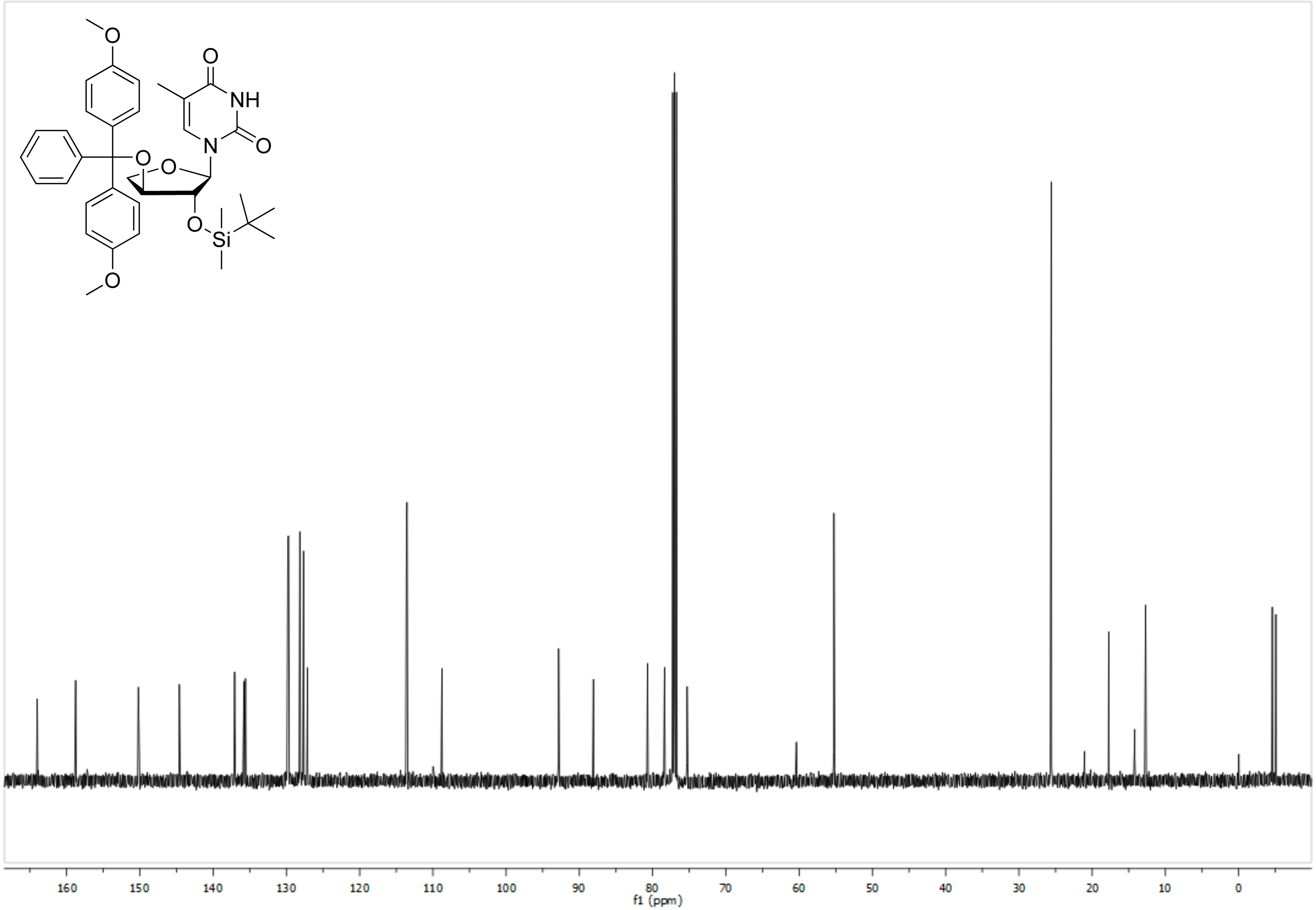

Supporting Figure 3: 500 MHz ^{1}H NMR spectrum of compound **3a** in $CDCl_3$

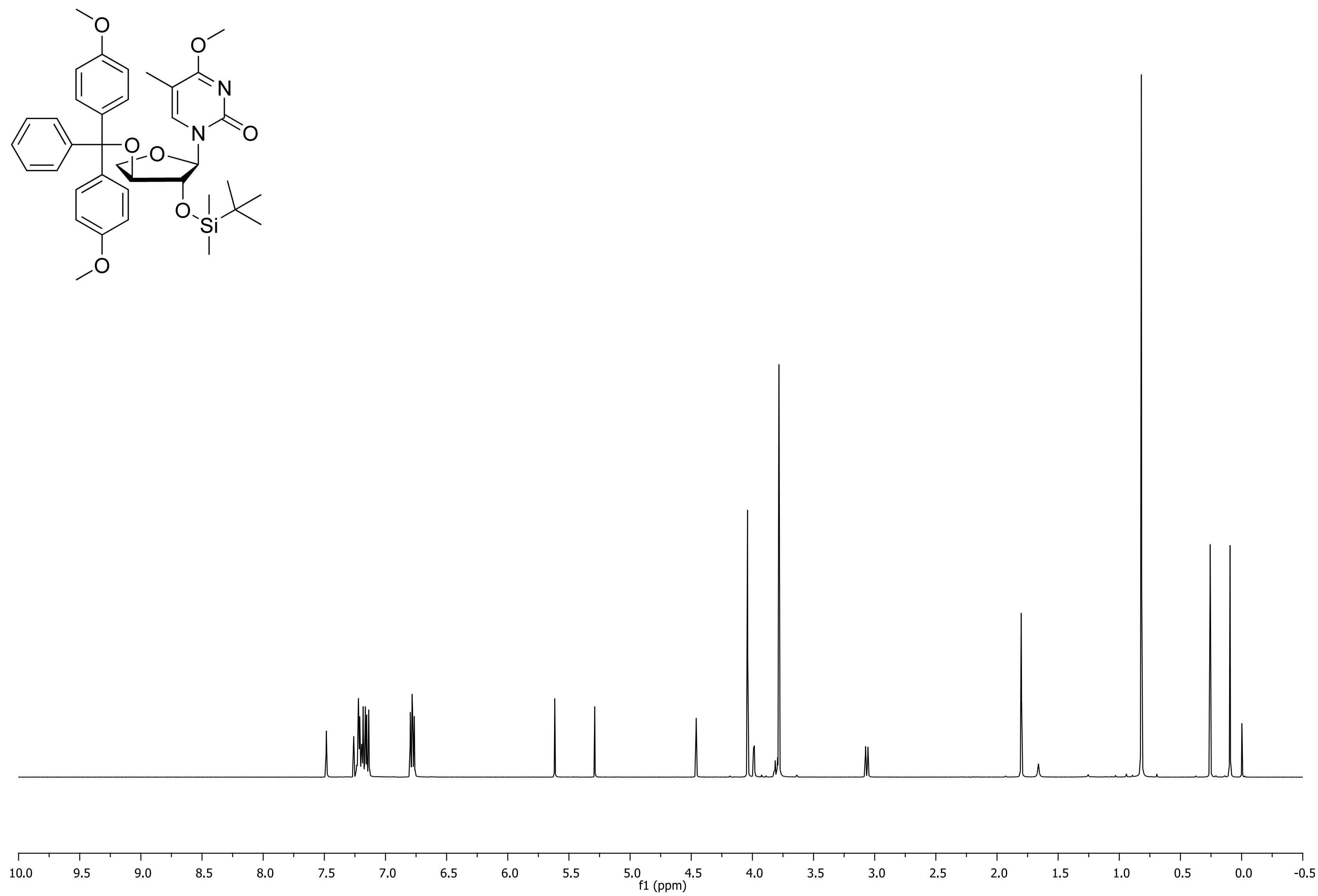

Supporting Figure 4: 125.7 MHz ^{13}C NMR spectrum of compound **3a** in $CDCl_3$

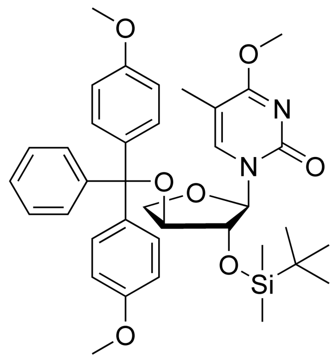

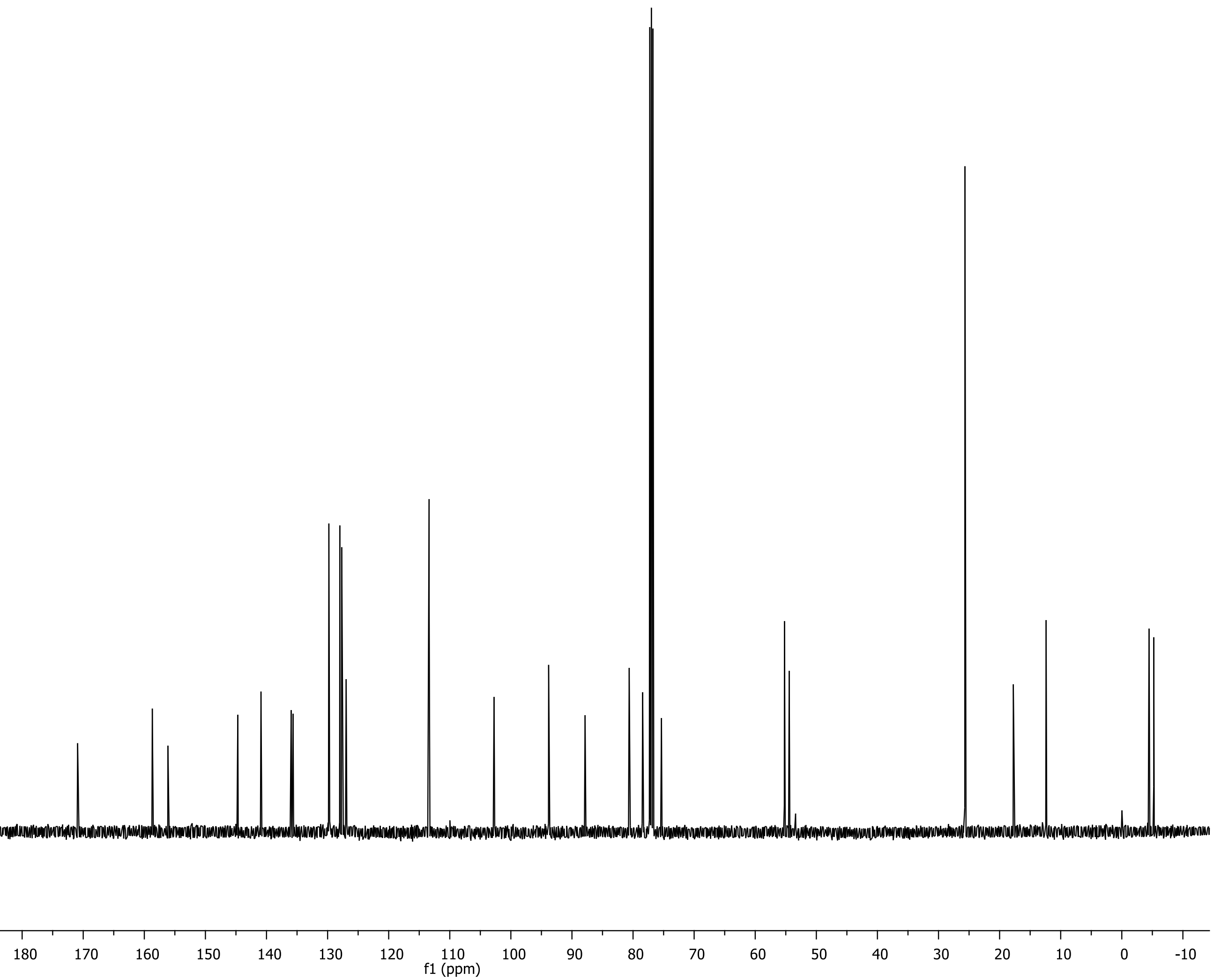

Supporting Figure 5: 500 MHz ^{1}H NMR spectrum of compound **3b** in $CDCl_3$

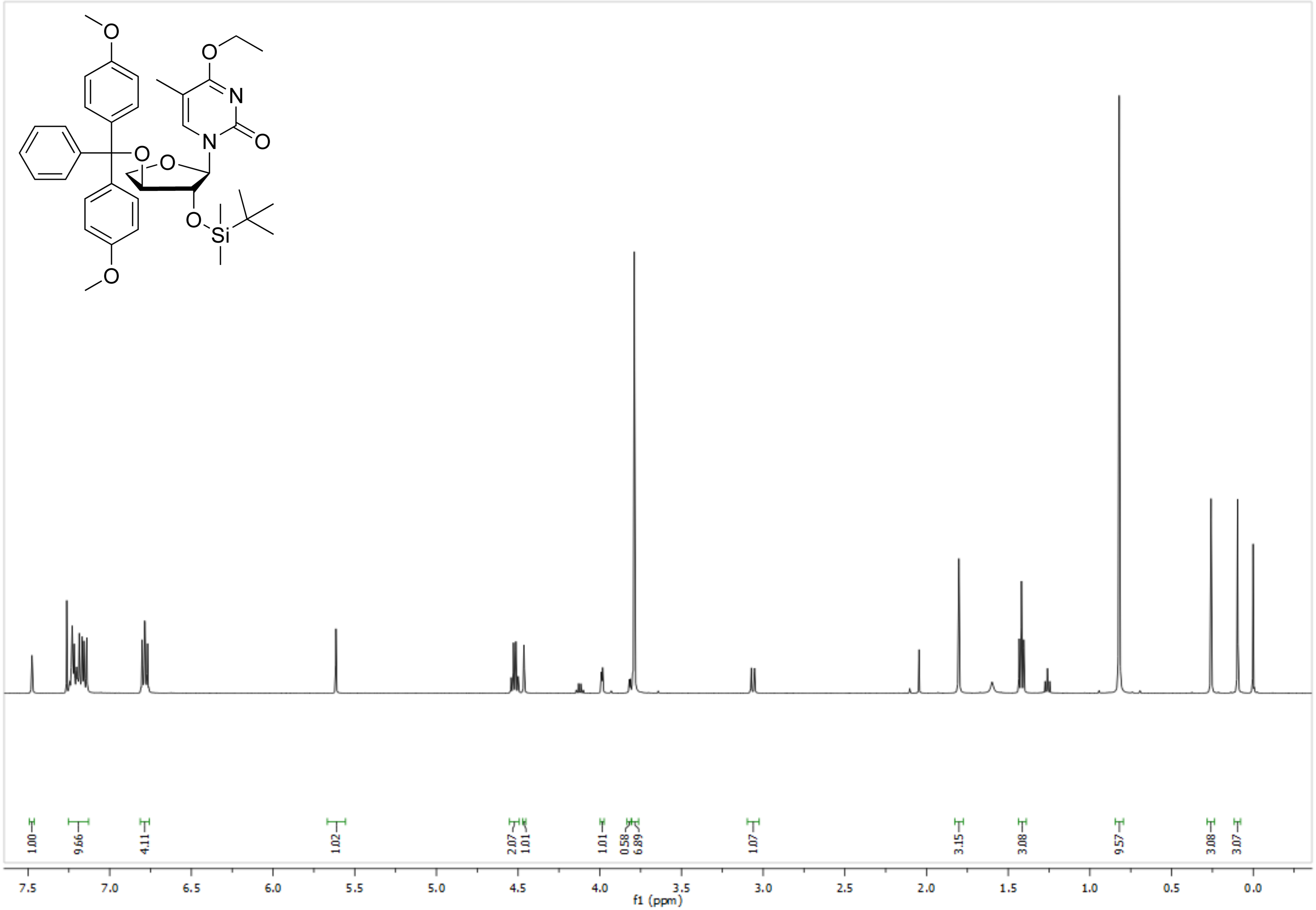

Supporting Figure 6: 125.7 MHz ^{13}C NMR spectrum of compound **3b** in $CDCl_3$

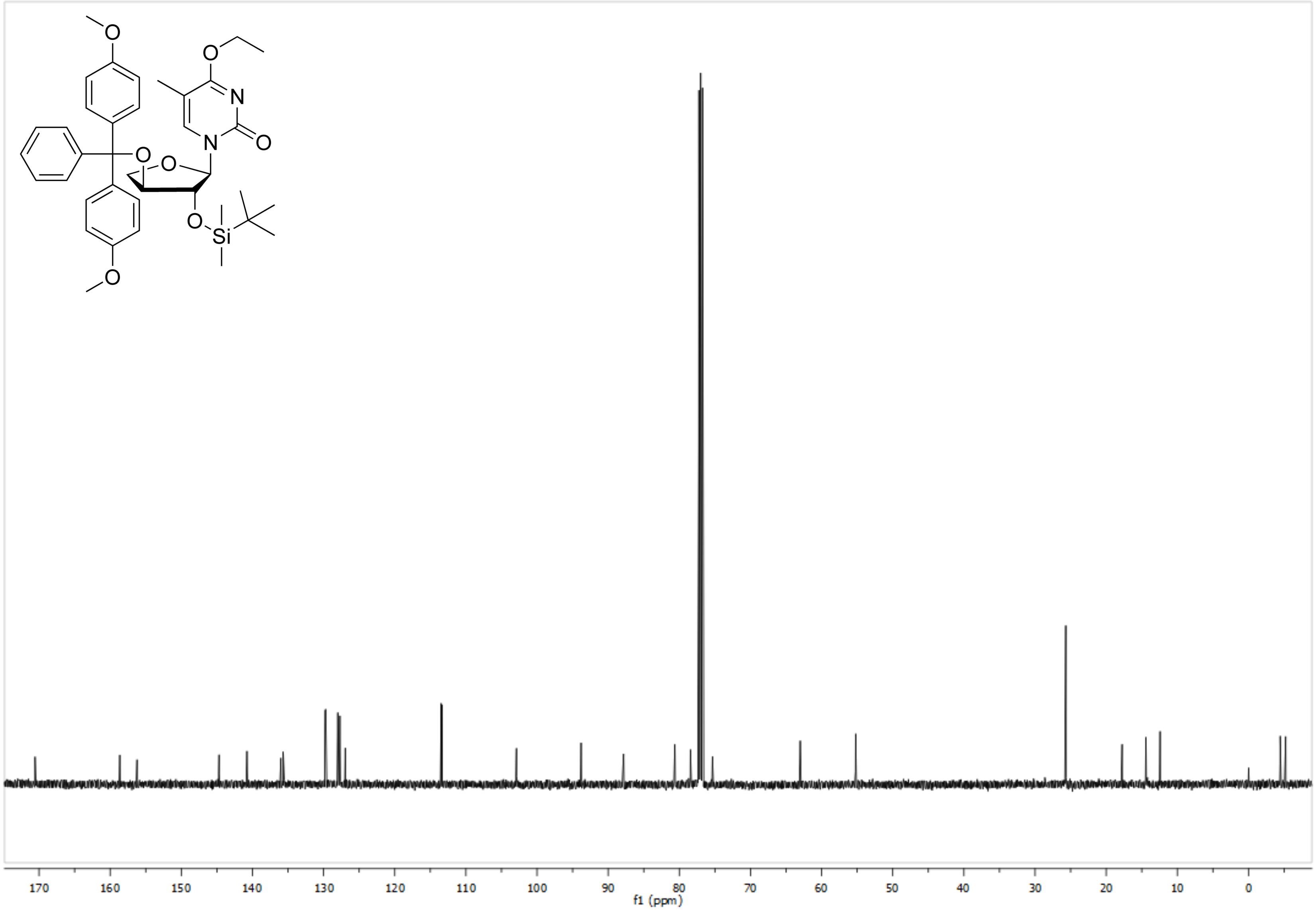

Supporting Figure 7: 500 MHz ^{1}H NMR spectrum of compound **4a** in $CDCl_3$

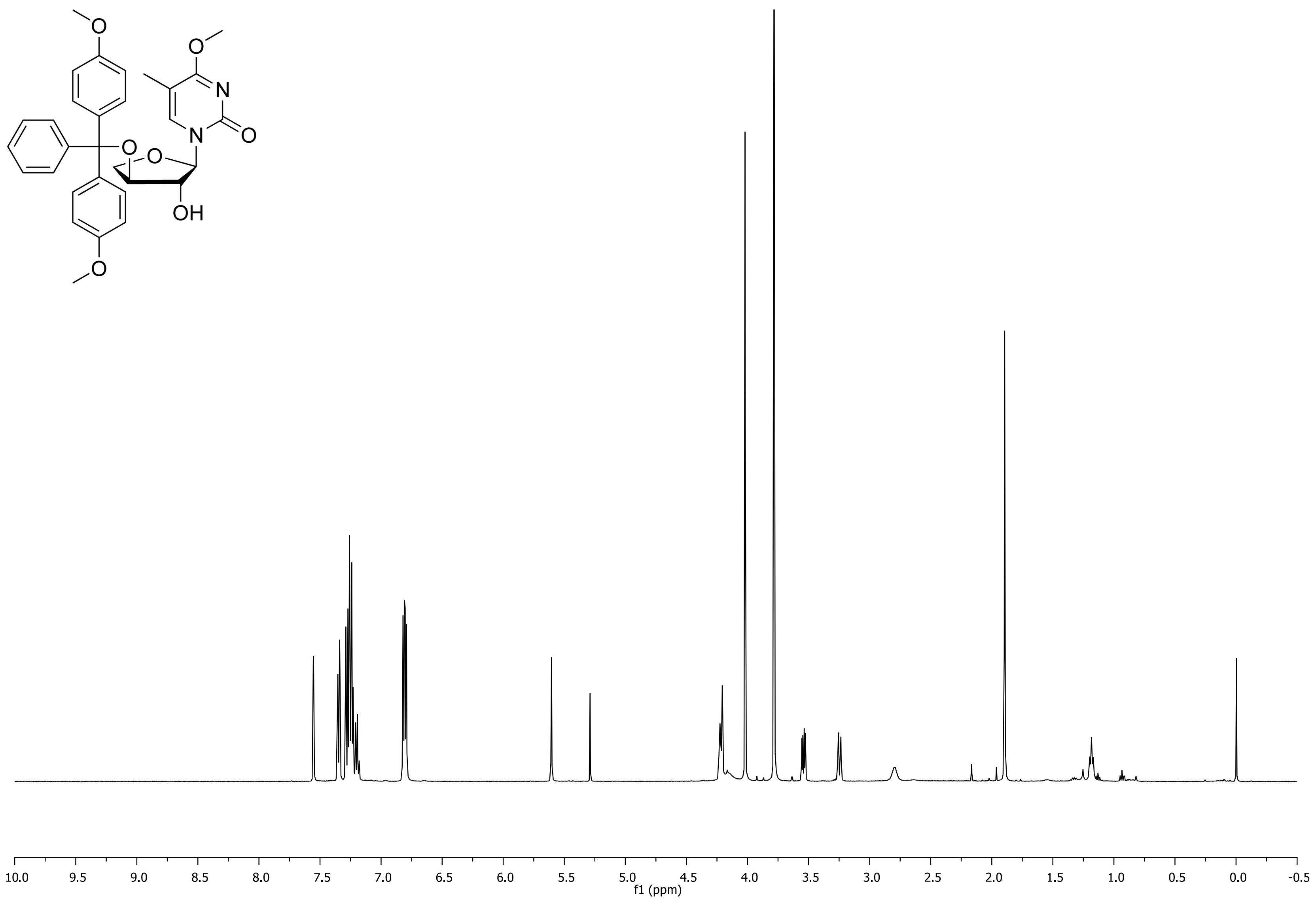

Supporting Figure 8: 125.7 MHz ^{13}C NMR spectrum of compound **4a** in $CDCl_3$

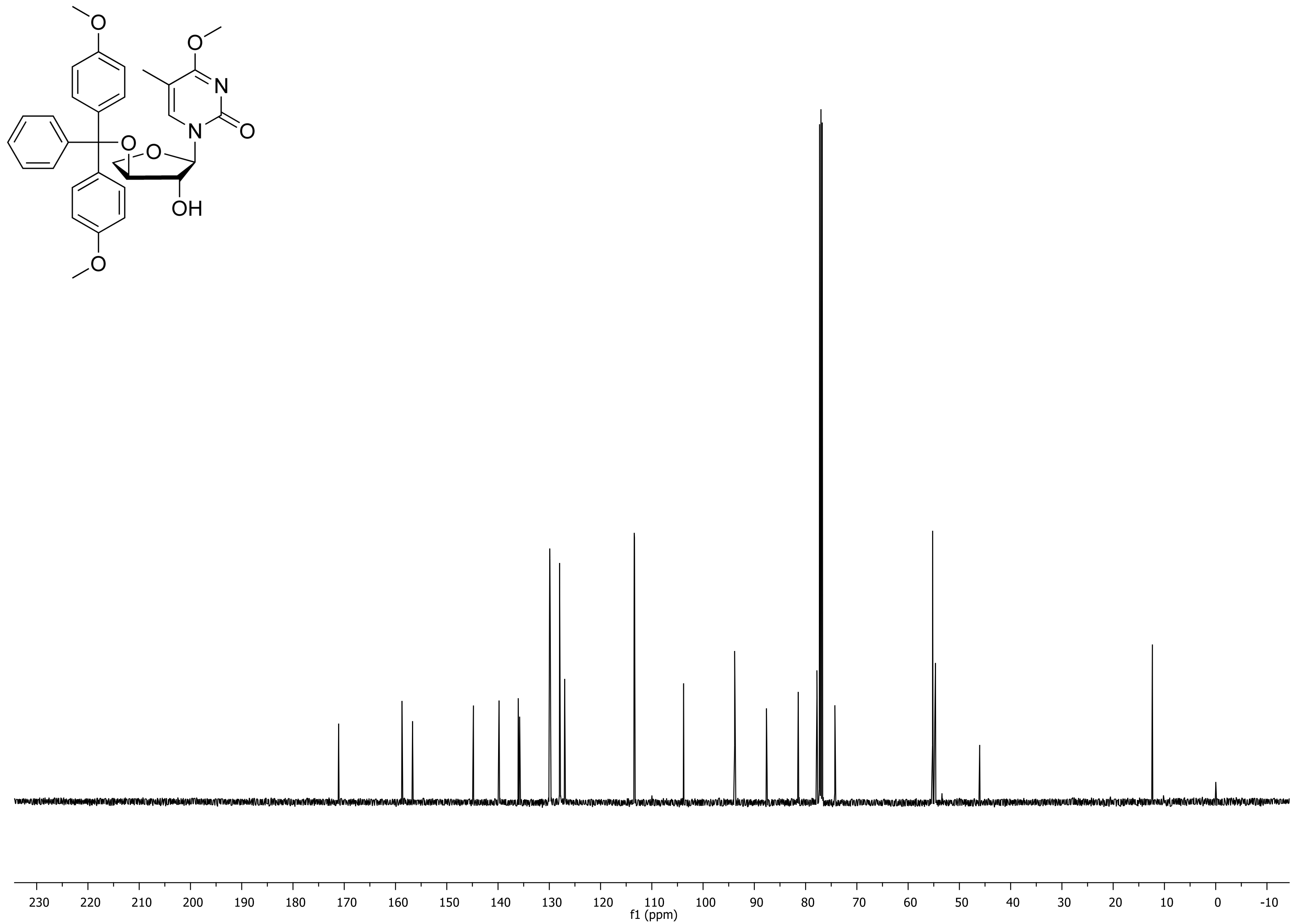

Supporting Figure 9: 500 MHz ^{1}H NMR spectrum of compound **4b** in $CDCl_3$

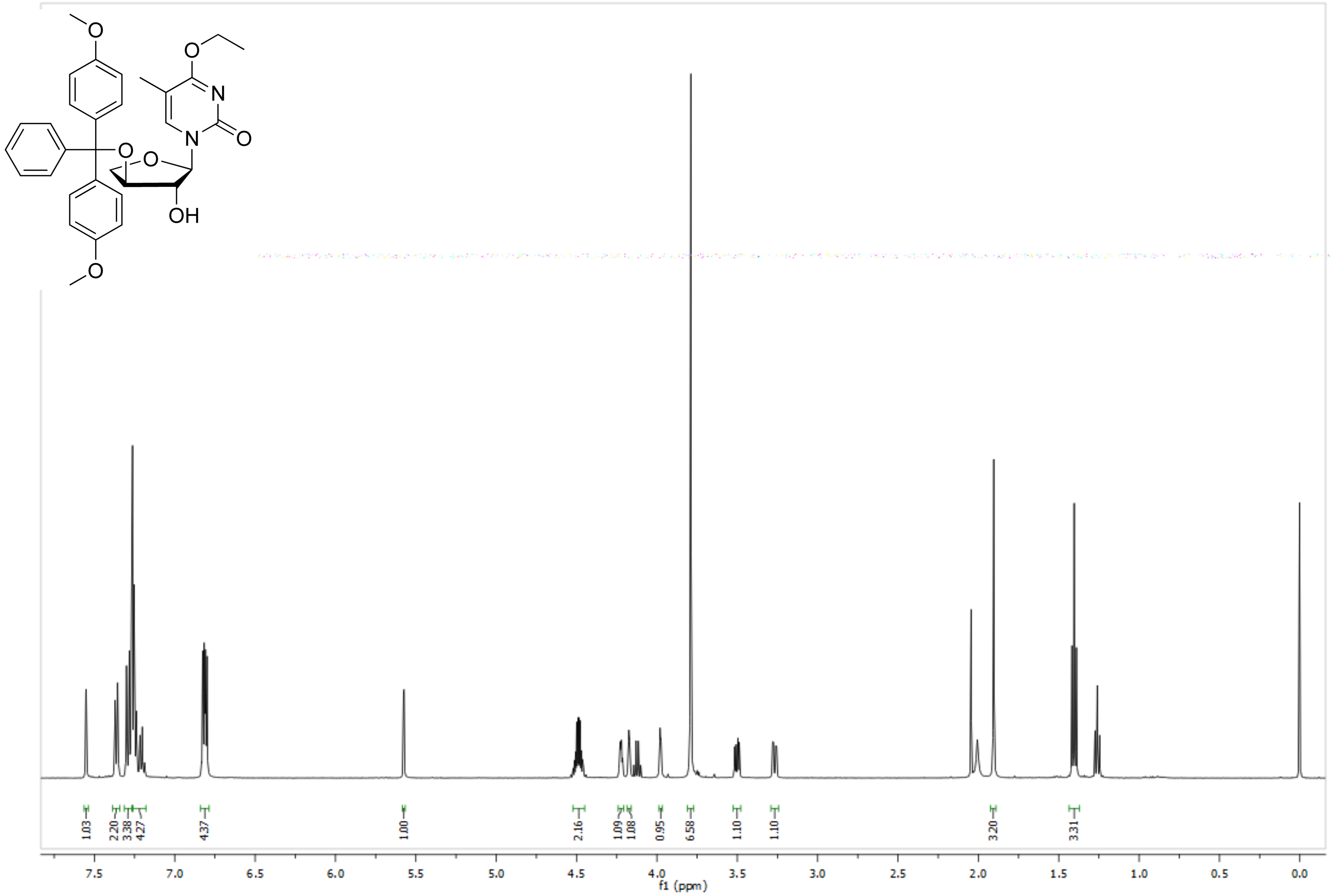

Supporting Figure 10: 125.7 MHz ^{13}C NMR spectrum of compound **4b** in $CDCl_3$

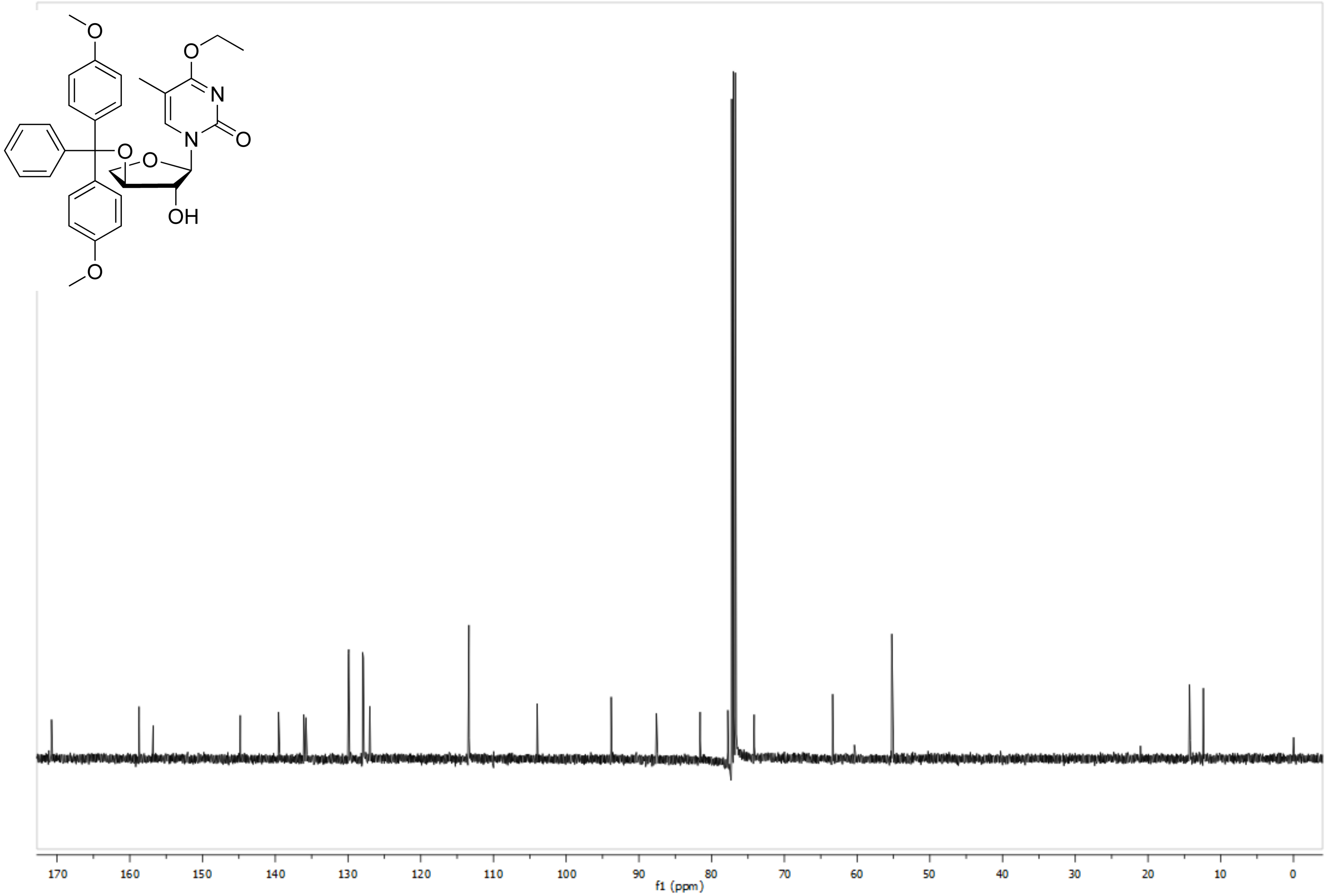

Supporting Figure 11: 500 MHz ^{1}H NMR spectrum of compound **5a** in d_6-acetone

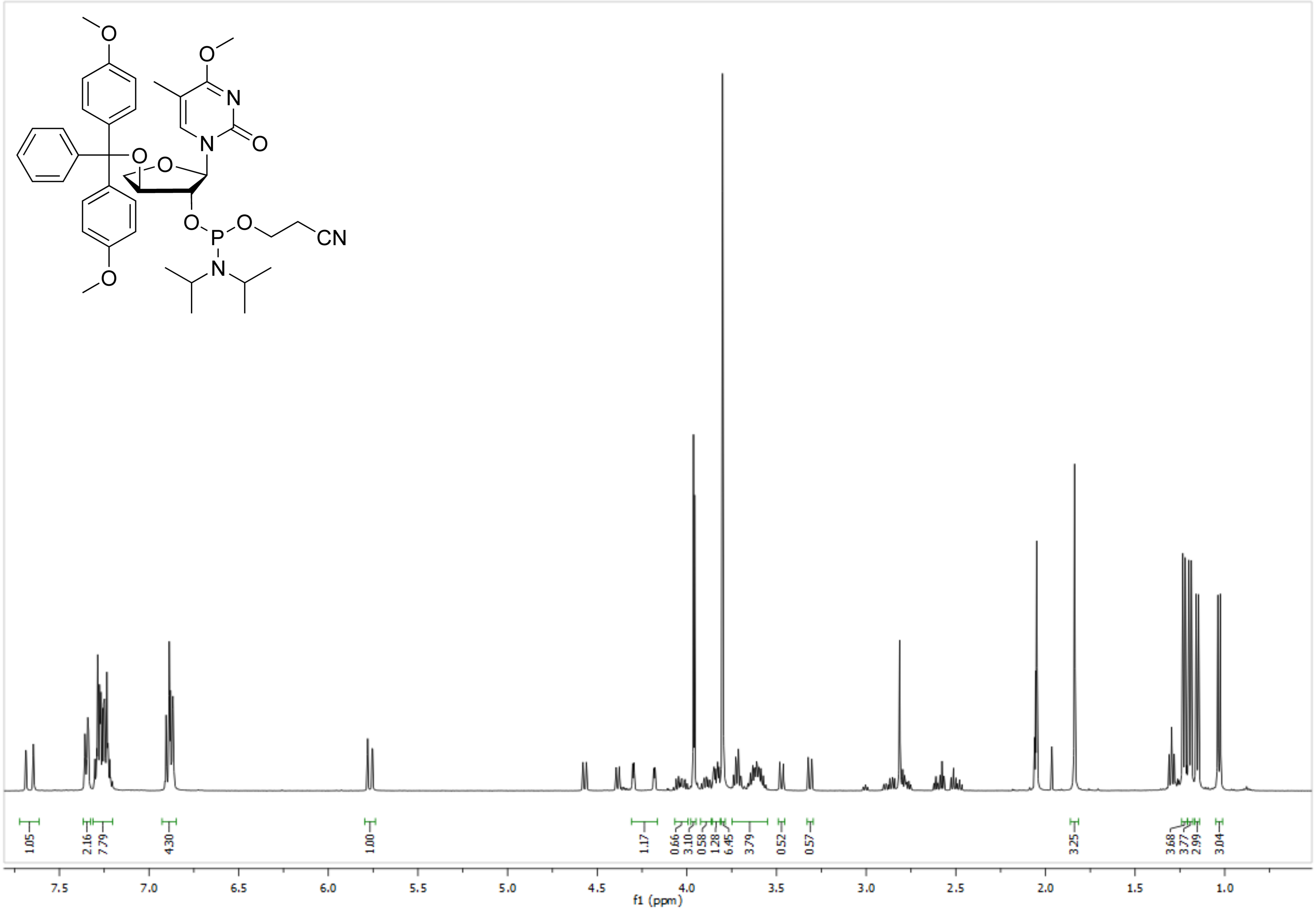

Supporting Figure 12: 125.7 MHz ^{13}C NMR spectrum of compound **5a** in d_6-acetone

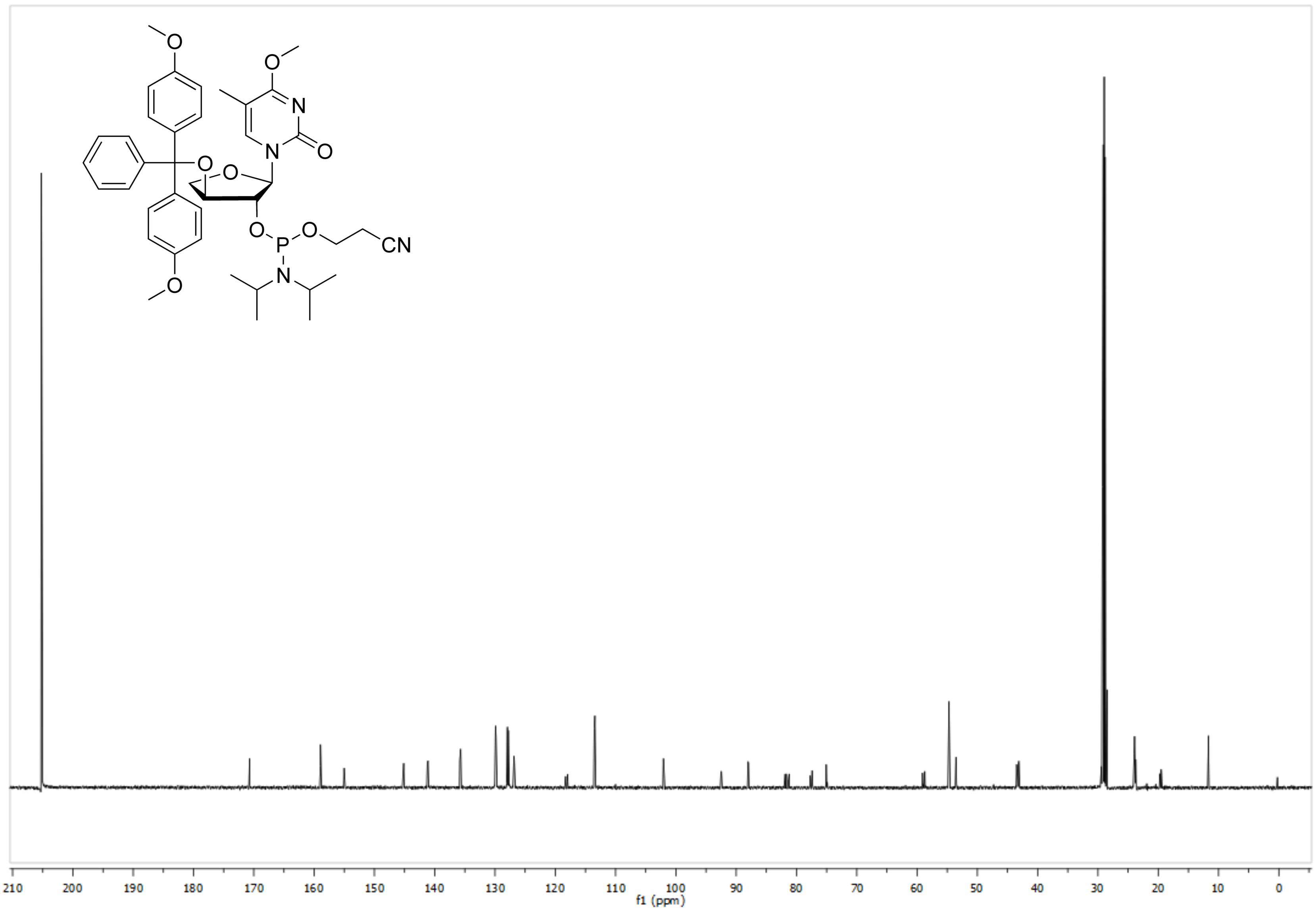

Supporting Figure 13: 202.3 MHz ^{31}P NMR spectrum of compound **5a** in d_6-acetone

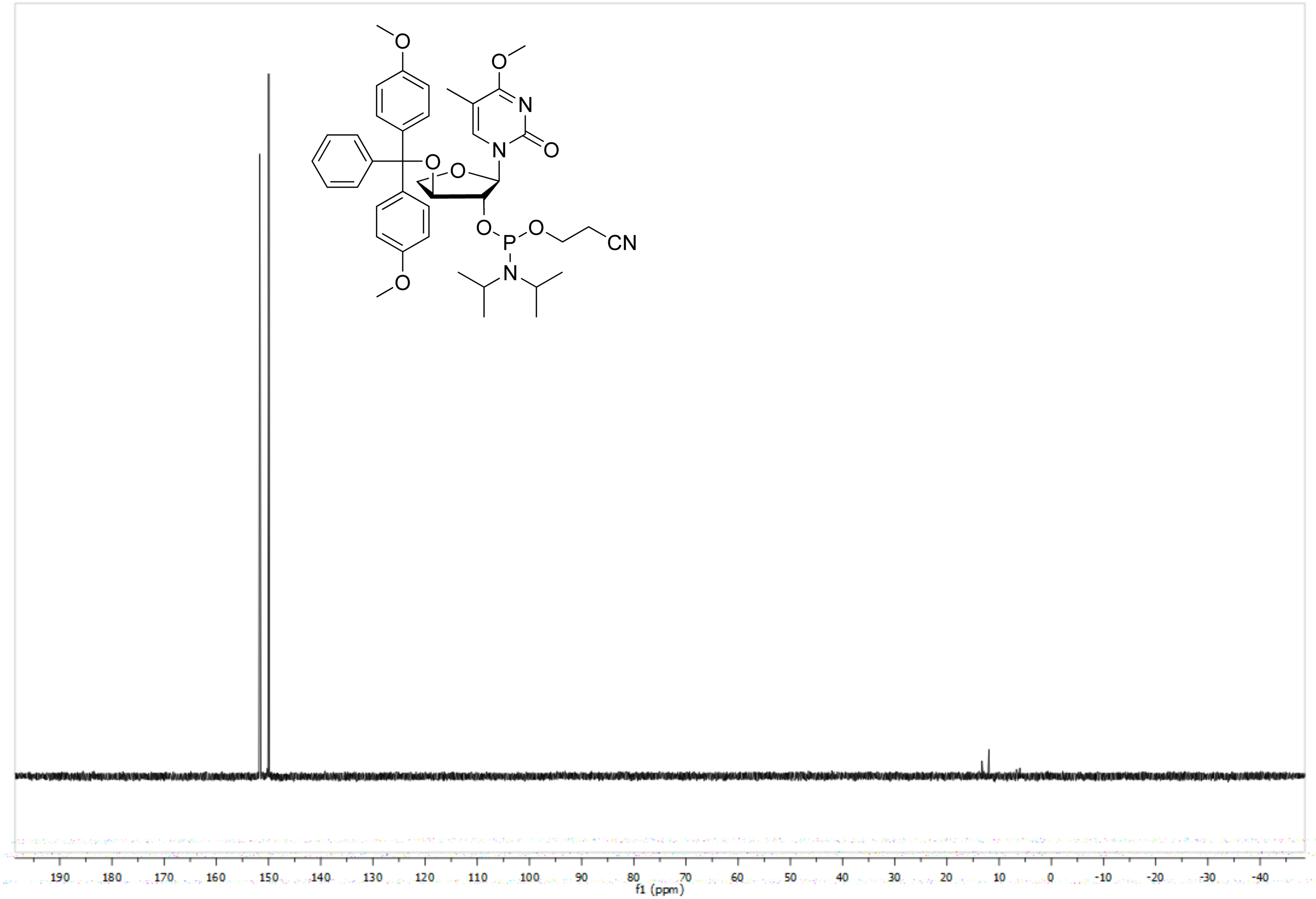

Supporting Figure 14: 500 MHz ^{1}H NMR spectrum of compound **5b** in d_6-acetone

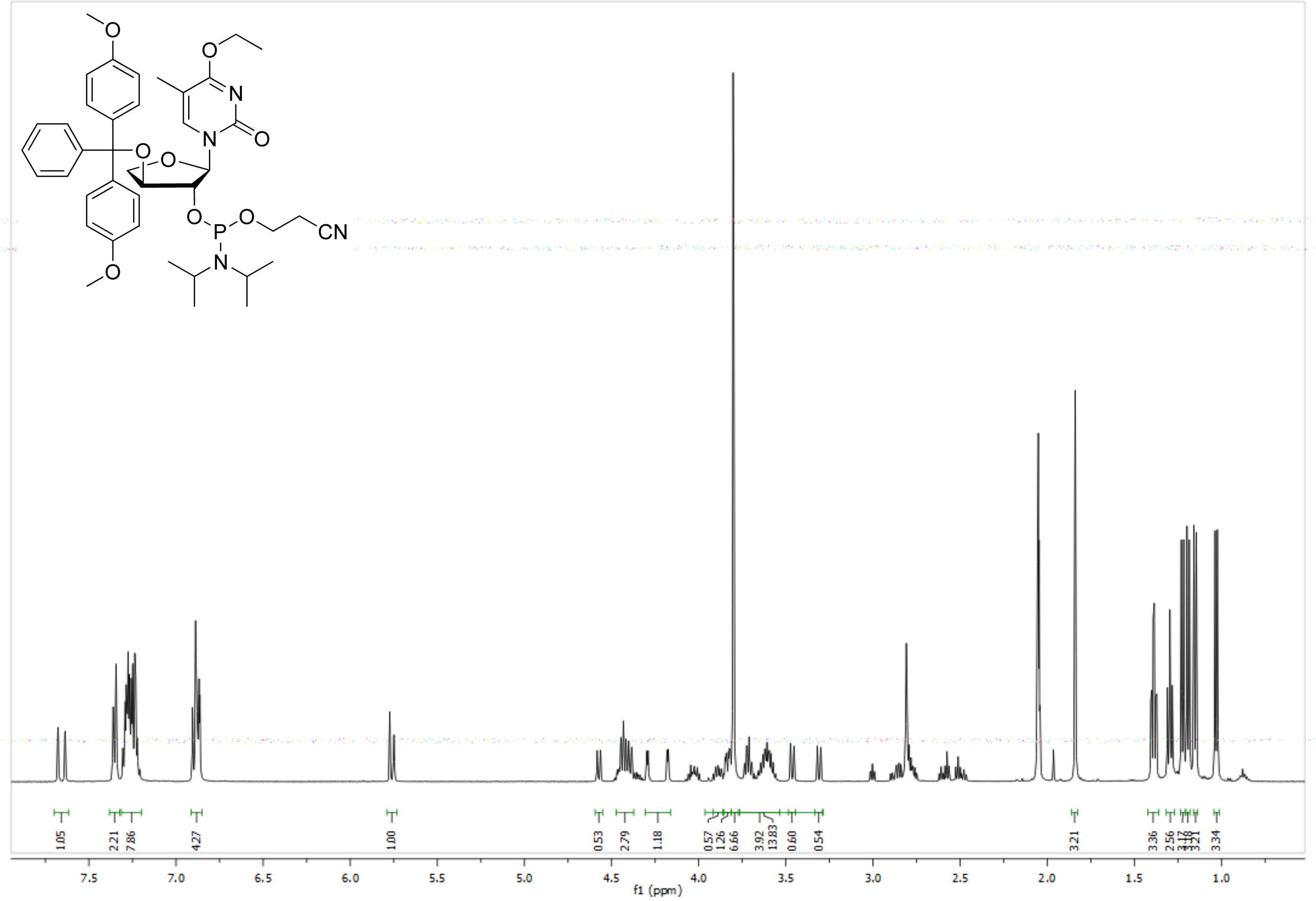

Supporting Figure 15: 125.7 MHz ^{13}C NMR spectrum of compound **5b** in d_6-acetone

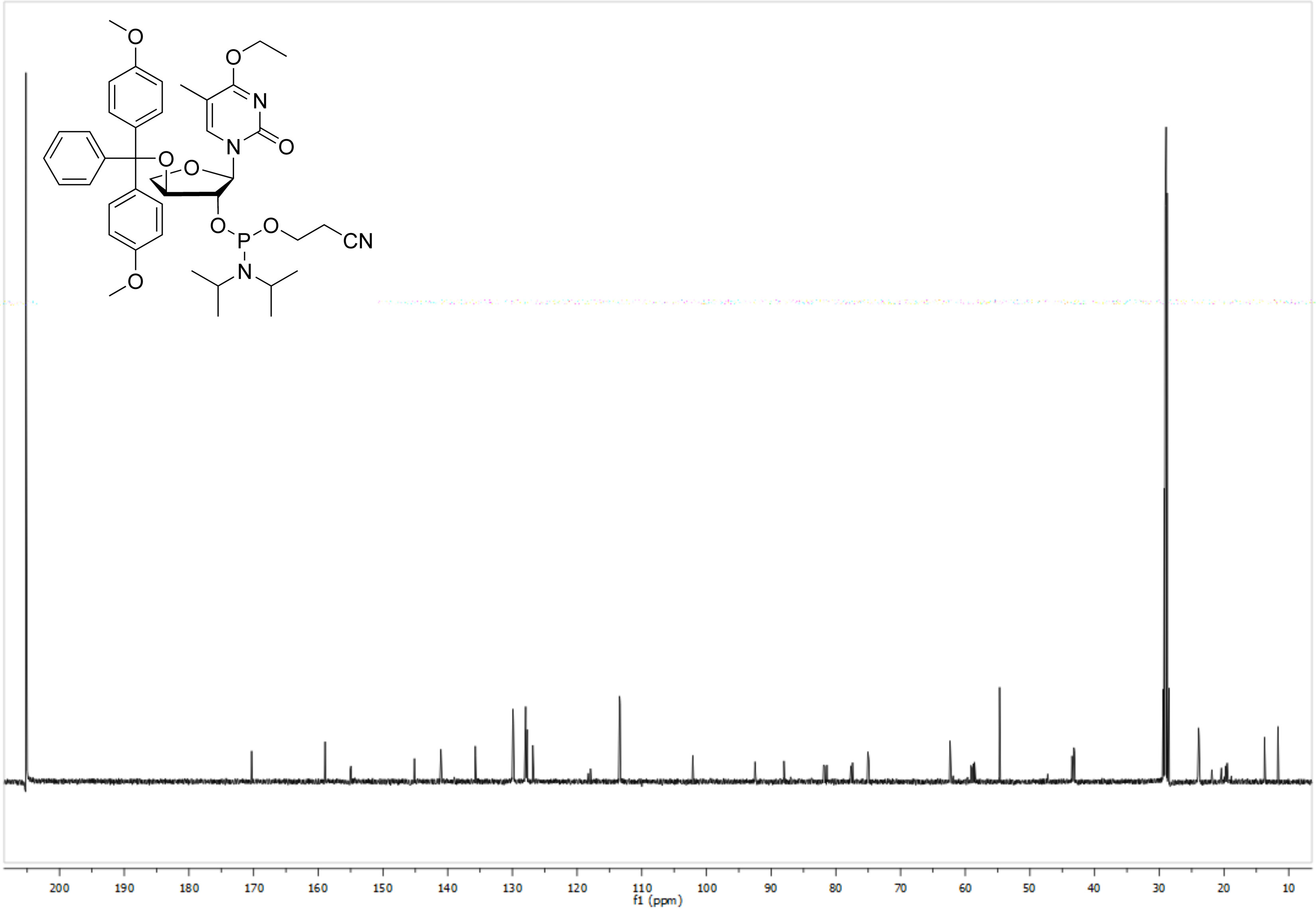

Supporting Figure 16: 202.3 MHz ^{31}P NMR spectrum of compound **5b** in d_6-acetone

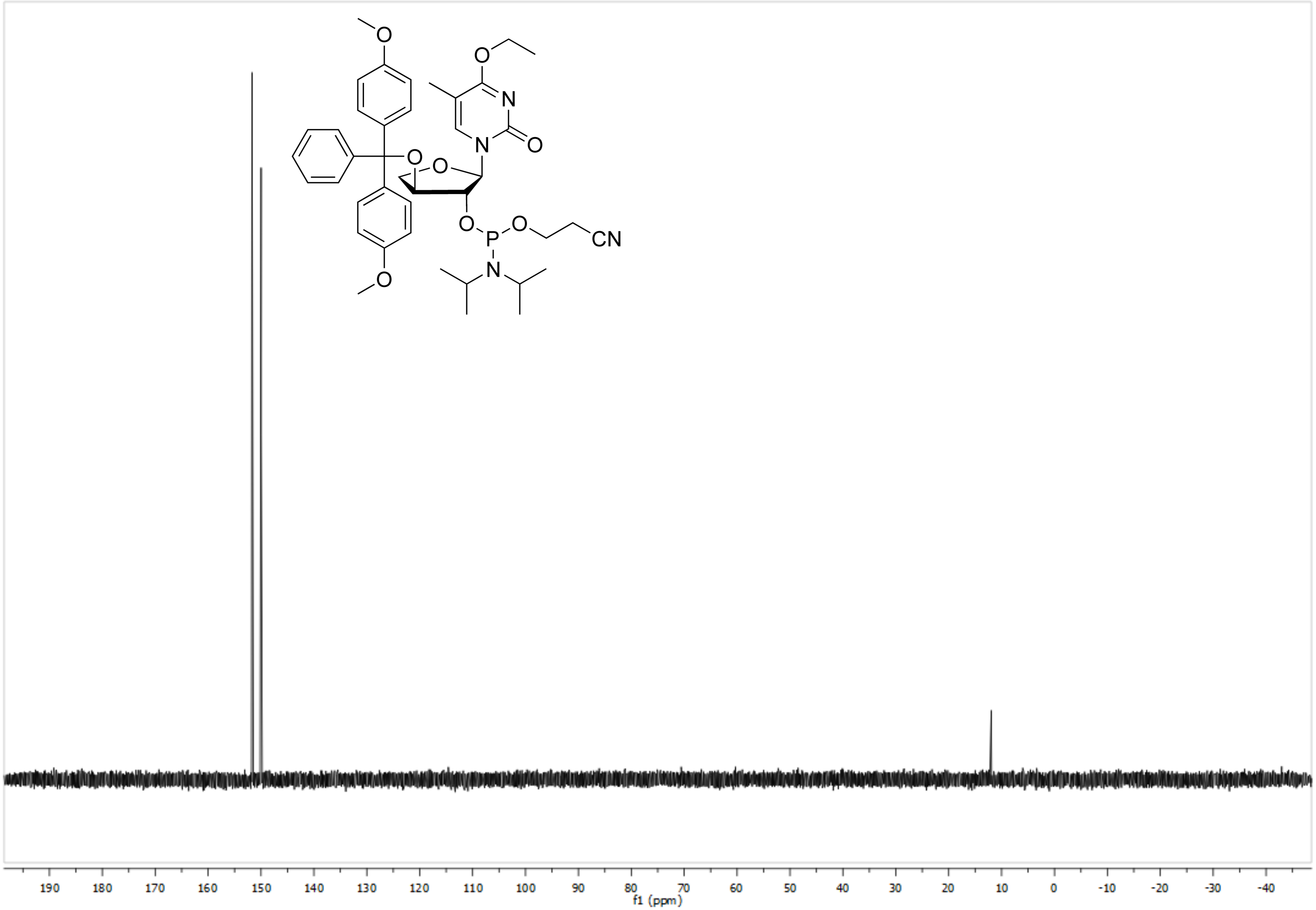

Supporting Figure 17: SAX-HPLC chromatograph of purified 5'-AGC GTC AT-3'

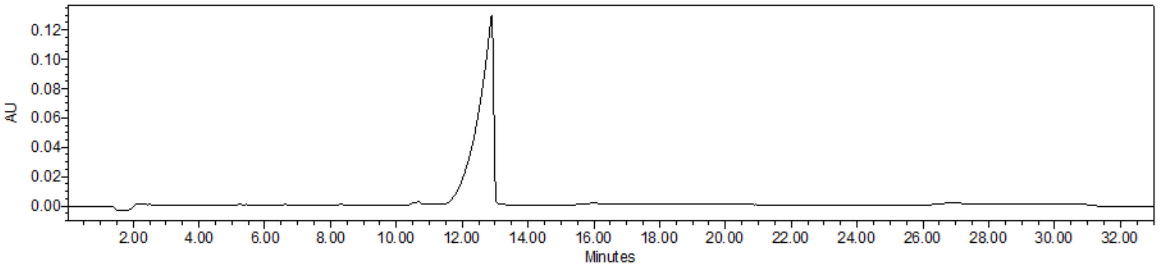

Gradient elution of 0-52.5 % B over 24 minutes. Buffer A: 100 mM Tris HCl, pH 7.5, 10% ACN and Buffer B: 100 mM Tris HCl, pH 7.5, 10% ACN, 1 M NaCl. The column was monitored at 260 nm.

Supporting Figure 18: ESI MS spectrum of purified 5'-AGC GTC AT-3'

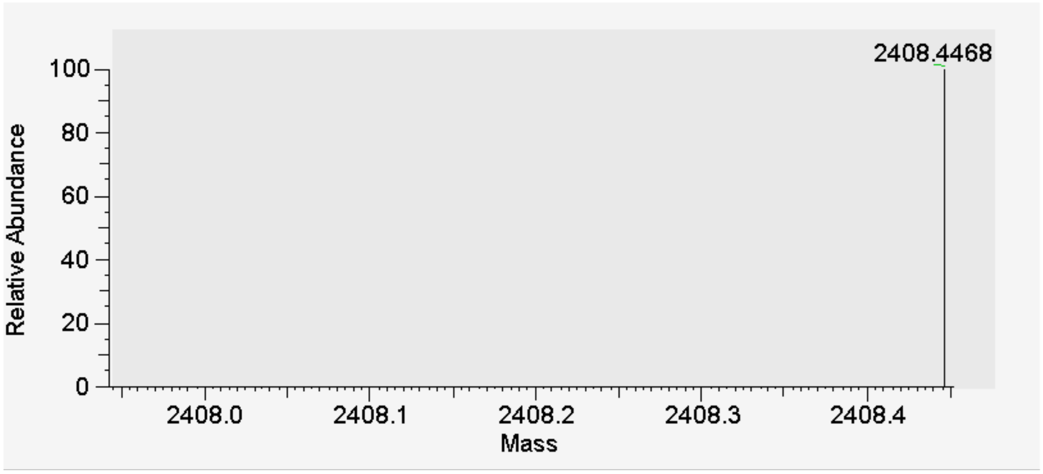

Supporting Figure 19: SAX-HPLC chromatograph of purified 5'-AGC GTC AA-3'

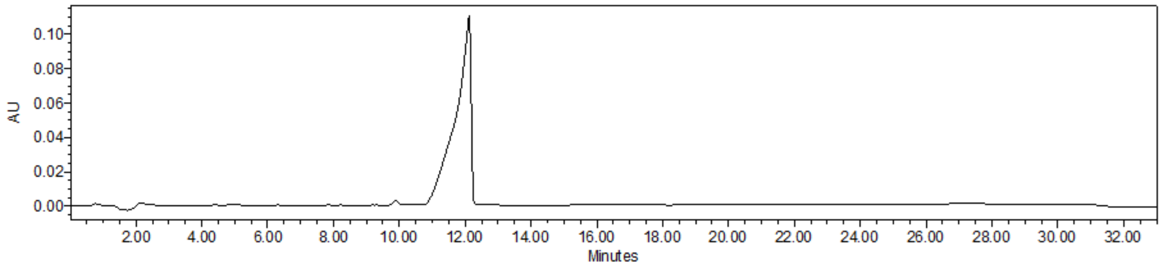

Gradient elution of 0-52.5 % B over 24 minutes. Buffer A: 100 mM Tris HCl, pH 7.5, 10% ACN and Buffer B: 100 mM Tris HCl, pH 7.5, 10% ACN, 1 M NaCl. The column was monitored at 260 nm.

Supporting Figure 20: ESI MS spectrum of purified 5'-AGC GTC AA-3'

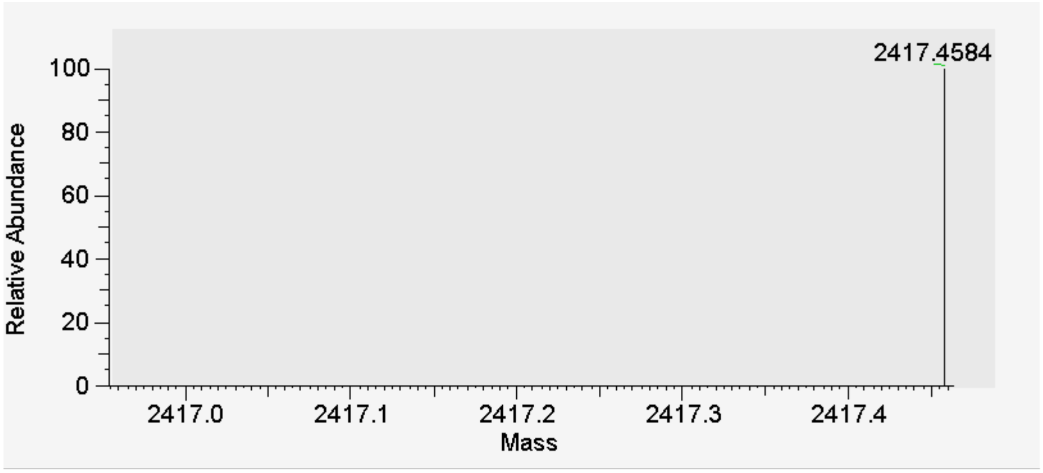

Supporting Figure 21: SAX-HPLC chromatograph of purified 5'-TCA TTA TGA CGC TTA CGA-3'

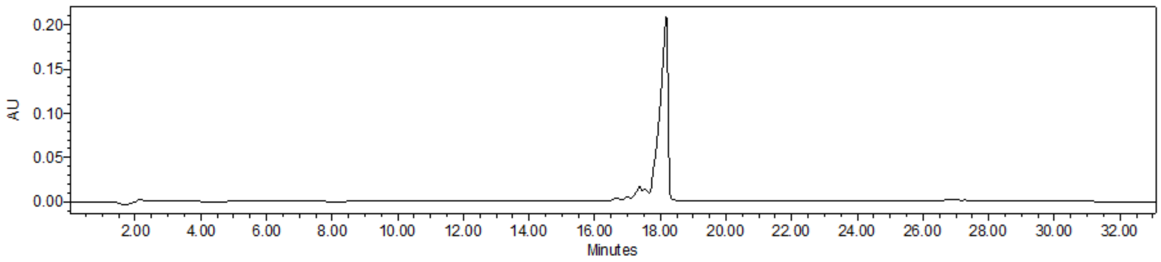

Gradient elution of 0-52.5 % B over 24 minutes. Buffer A: 100 mM Tris HCl, pH 7.5, 10% ACN and Buffer B: 100 mM Tris HCl, pH 7.5, 10% ACN, 1 M NaCl. The column was monitored at 260 nm.

Supporting Figure 22: ESI MS spectrum of purified 5'-TCA TTA TGA CGC TTA CGA-3'

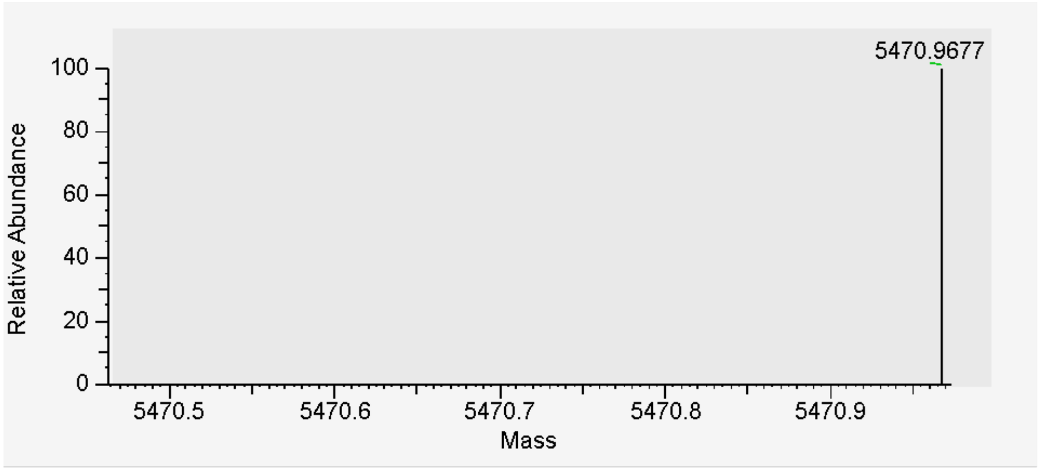

Supporting Figure 23: SAX-HPLC chromatograph of purified 5'-TCA TXA TGA CGC TTA CGA-3' (X = tT)

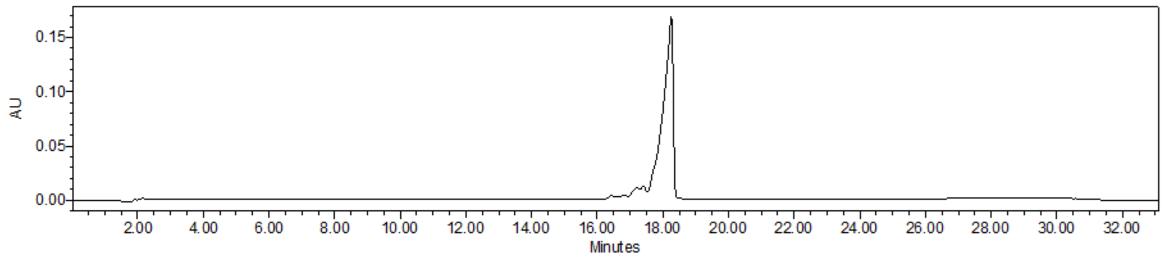

Gradient elution of 0-52.5 % B over 24 minutes. Buffer A: 100 mM Tris HCl, pH 7.5, 10% ACN and Buffer B: 100 mM Tris HCl, pH 7.5, 10% ACN, 1 M NaCl. The column was monitored at 260 nm.

Supporting Figure 24: ESI MS spectrum of purified 5'-TCA TXA TGA CGC TTA CGA-3' (X = tT)

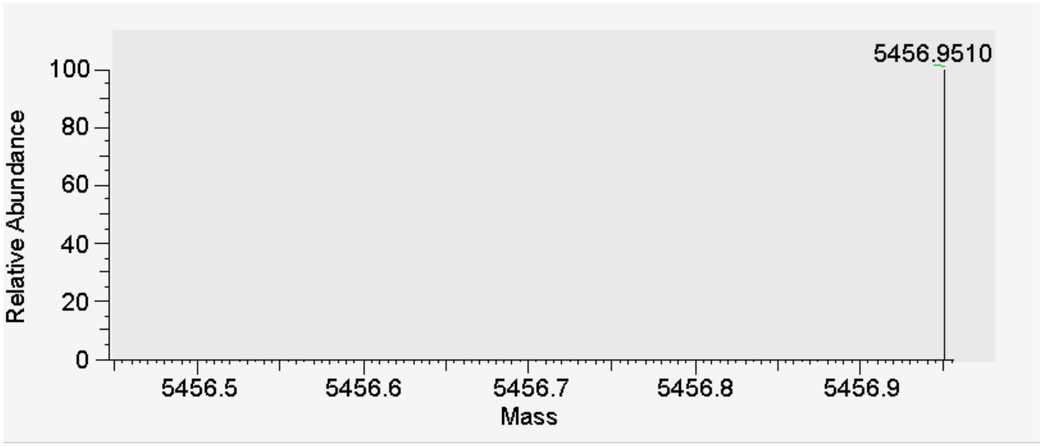

Supporting Figure 25: SAX-HPLC chromatograph of purified 5'-CAT XAT GAC GCT-3' (X = tT)

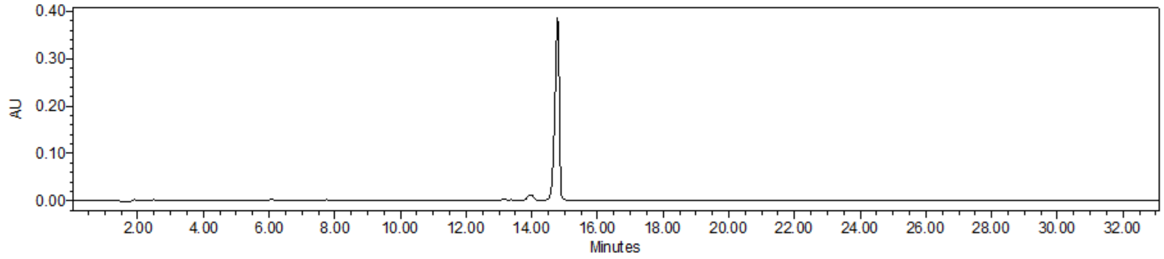

Gradient elution of 0-52.5 % B over 24 minutes. Buffer A: 100 mM Tris HCl, pH 7.5, 10% ACN and Buffer B: 100 mM Tris HCl, pH 7.5, 10% ACN, 1 M NaCl. The column was monitored at 260 nm.

Supporting Figure 26: ESI MS spectrum of purified 5'-CAT XAT GAC GCT-3' (X = tT)

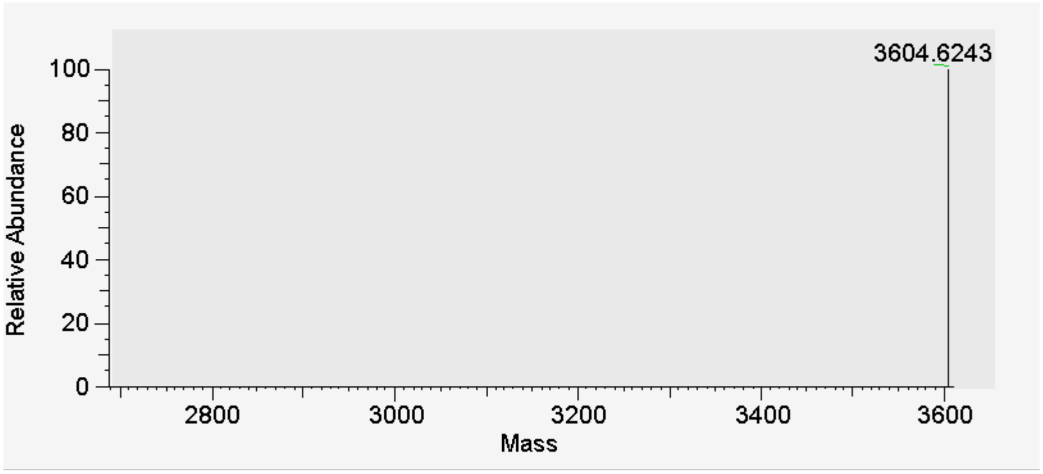

Supporting Figure 27: SAX-HPLC chromatograph of purified 5'-CAT GXT GAC GCT-3' (X = tT)

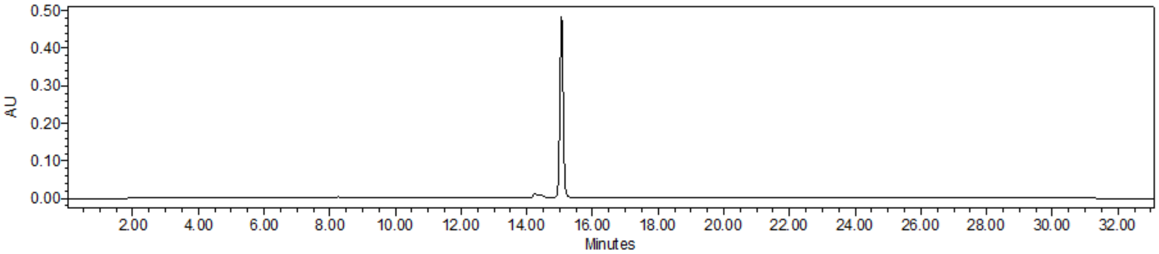

Gradient elution of 0-52.5 % B over 24 minutes. Buffer A: 100 mM Tris HCl, pH 7.5, 10% ACN and Buffer B: 100 mM Tris HCl, pH 7.5, 10% ACN, 1 M NaCl. The column was monitored at 260 nm.

Supporting Figure 28: ESI MS spectrum of purified 5'-CAT GXT GAC GCT-3' (X = tT)

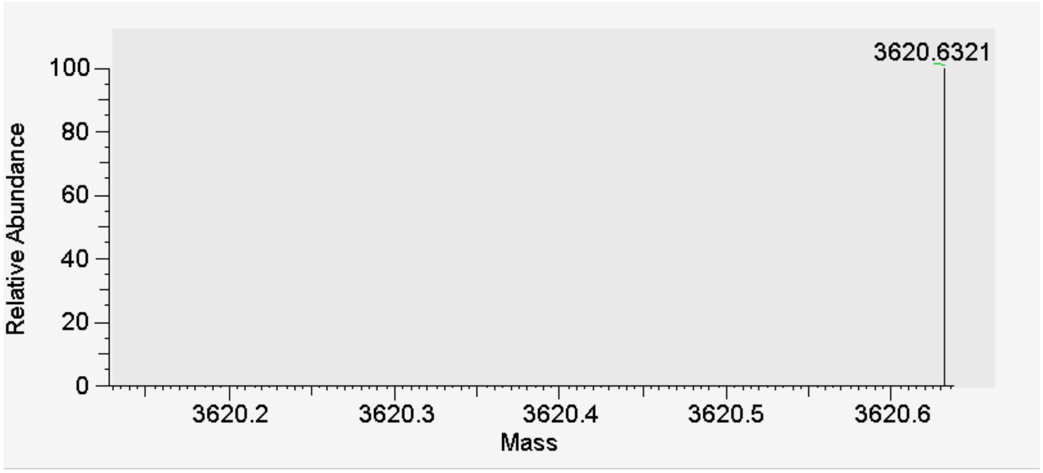

Supporting Figure 29: SAX-HPLC chromatograph of purified 5'-CAT XAT GAC GCT-3' (X = O^4-Me tT)

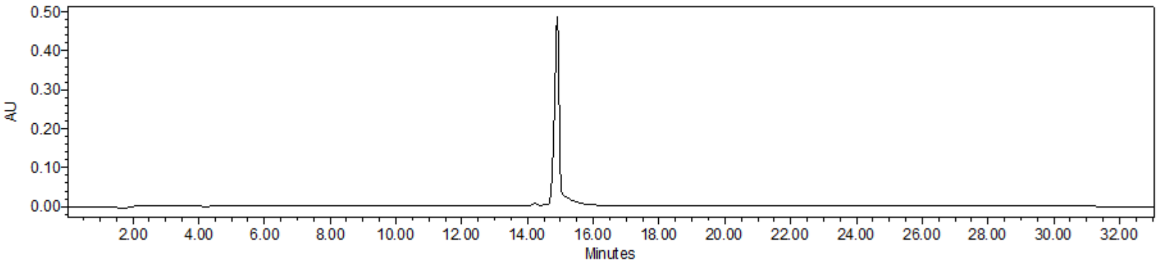

Gradient elution of 0-52.5 % B over 24 minutes. Buffer A: 100 mM Tris HCl, pH 7.5, 10% ACN and Buffer B: 100 mM Tris HCl, pH 7.5, 10% ACN, 1 M NaCl. The column was monitored at 260 nm.

Supporting Figure 30: ESI MS spectrum of purified 5'-CAT XAT GAC GCT-3' (X = O^4-Me tT)

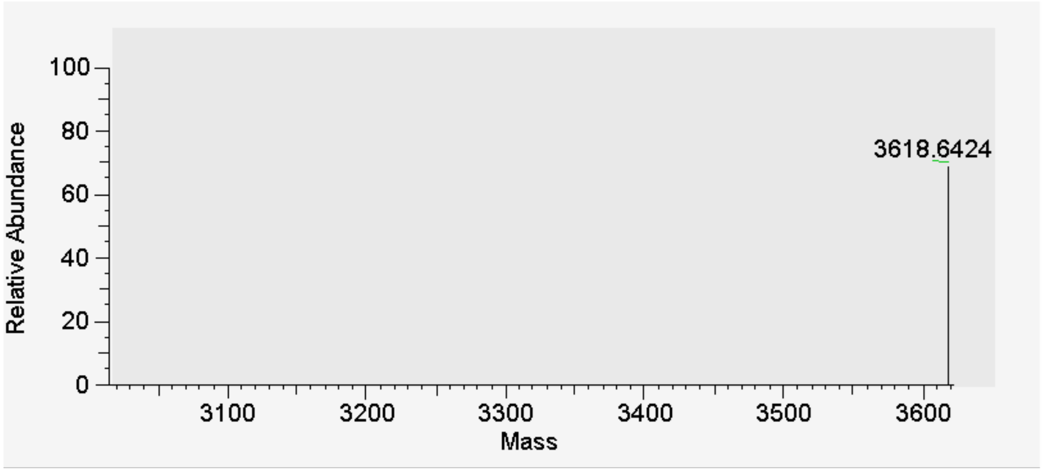

Supporting Figure 31: SAX-HPLC chromatograph of purified 5'-CAT XAT GAC GCT-3' ($X = O^4$-Et tT)

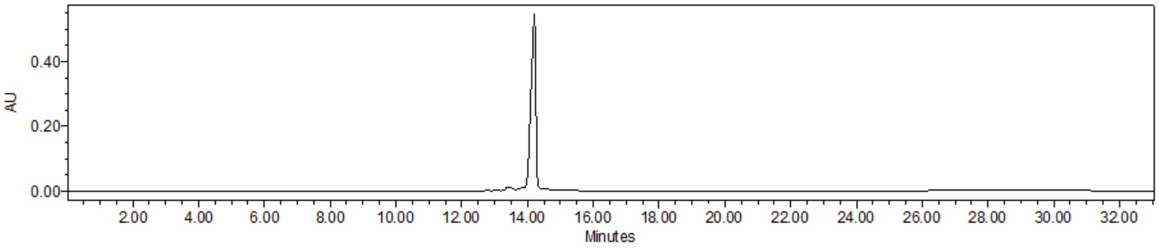

Gradient elution of 0-52.5 % B over 24 minutes. Buffer A: 100 mM Tris HCl, pH 7.5, 10% ACN and Buffer B: 100 mM Tris HCl, pH 7.5, 10% ACN, 1 M NaCl. The column was monitored at 260 nm.

Supporting Figure 32: ESI MS spectrum of purified 5'-CAT XAT GAC GCT-3' ($X = O^4$-Et tT)

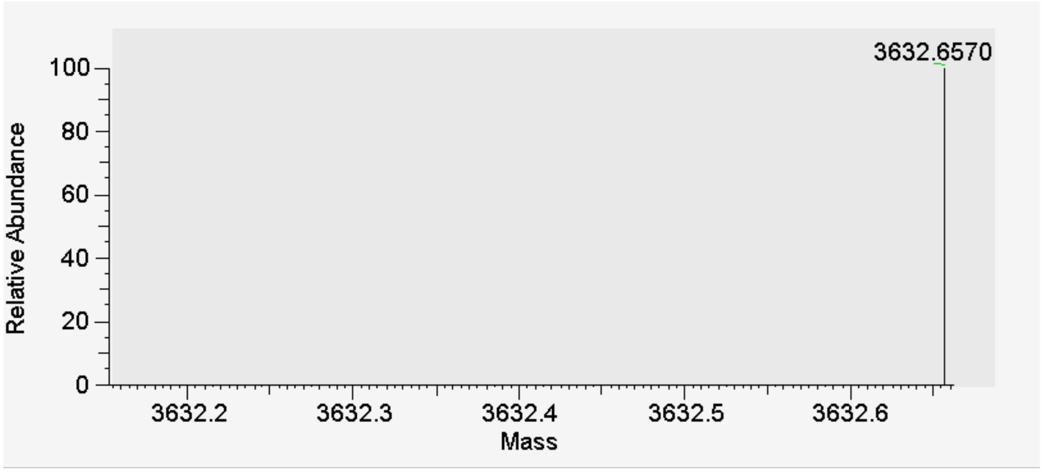

Supporting Figure 33: SAX-HPLC chromatograph of purified 5'-CAT GXT GAC GCT-3' (X = O^4-Me tT)

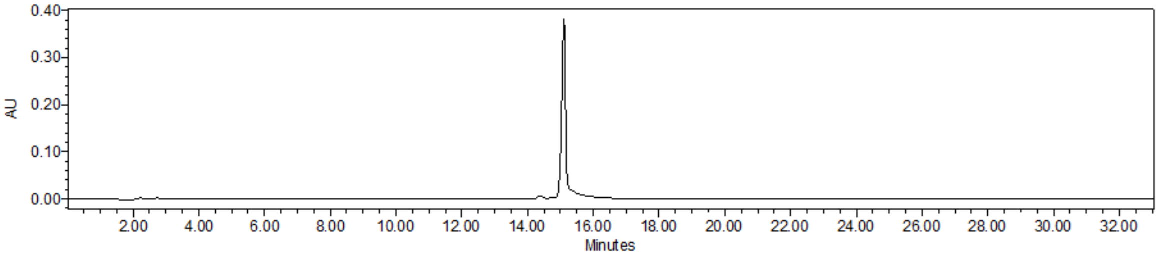

Gradient elution of 0-52.5 % B over 24 minutes. Buffer A: 100 mM Tris HCl, pH 7.5, 10% ACN and Buffer B: 100 mM Tris HCl, pH 7.5, 10% ACN, 1 M NaCl. The column was monitored at 260 nm.

Supporting Figure 34: ESI MS spectrum of purified 5'-CAT GXT GAC GCT-3' (X = O^4-Me tT)

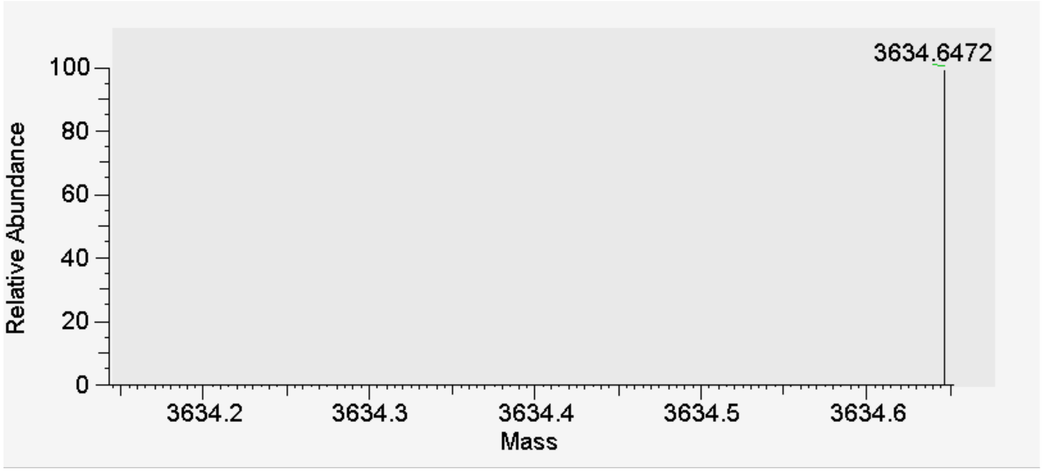

Supporting Figure 35: SAX-HPLC chromatograph of purified 5'-CAT GXT GAC GCT-3' (X = O^4-Et tT)

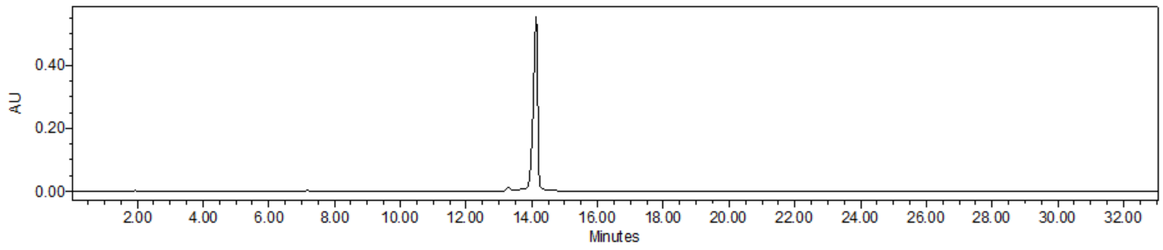

Gradient elution of 0-52.5 % B over 24 minutes. Buffer A: 100 mM Tris HCl, pH 7.5, 10% ACN and Buffer B: 100 mM Tris HCl, pH 7.5, 10% ACN, 1 M NaCl. The column was monitored at 260 nm.

Supporting Figure 36: ESI MS spectrum of purified 5'-CAT GXT GAC GCT-3' (X = O^4-Et tT)

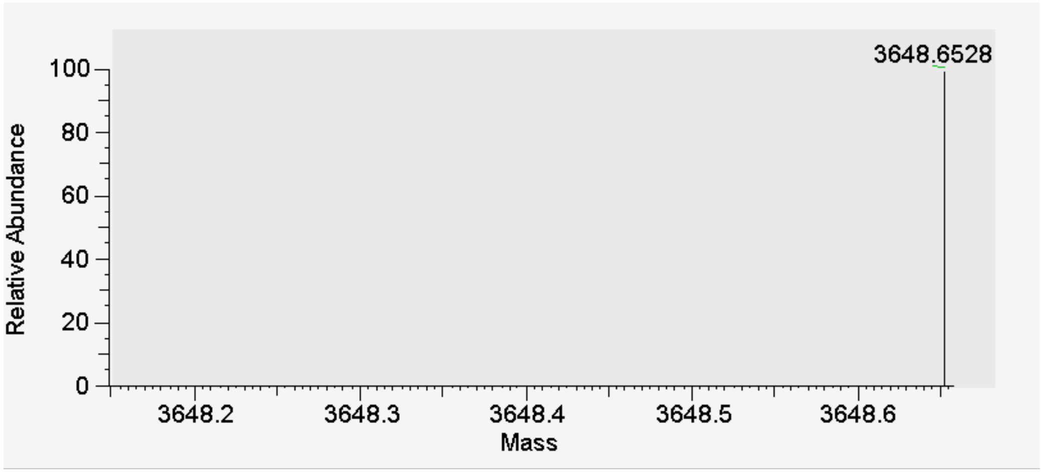

Supporting Figure 37: SAX -HPLC chromatograph of purified 5'-TCA TXA TGA CGC TTA CGA-3' (X = O^4-Me dT)

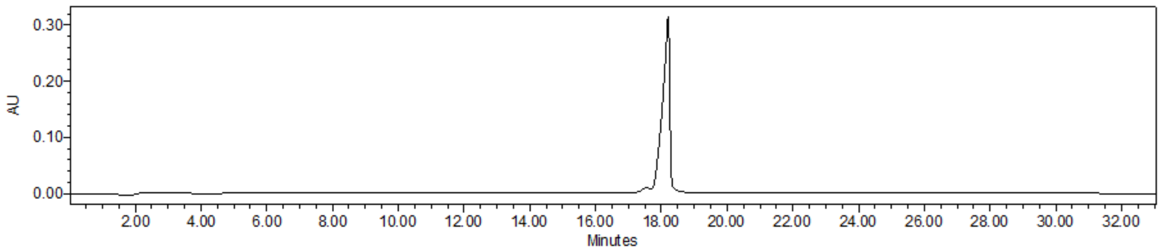

Gradient elution of 0-52.5 % B over 24 minutes. Buffer A: 100 mM Tris HCl, pH 7.5, 10% ACN and Buffer B: 100 mM Tris HCl, pH 7.5, 10% ACN, 1 M NaCl. The column was monitored at 260 nm.

Supporting Figure 38: ESI MS spectrum of purified 5'-TCA TXA TGA CGC TTA CGA-3' (X = O^4-Me dT)

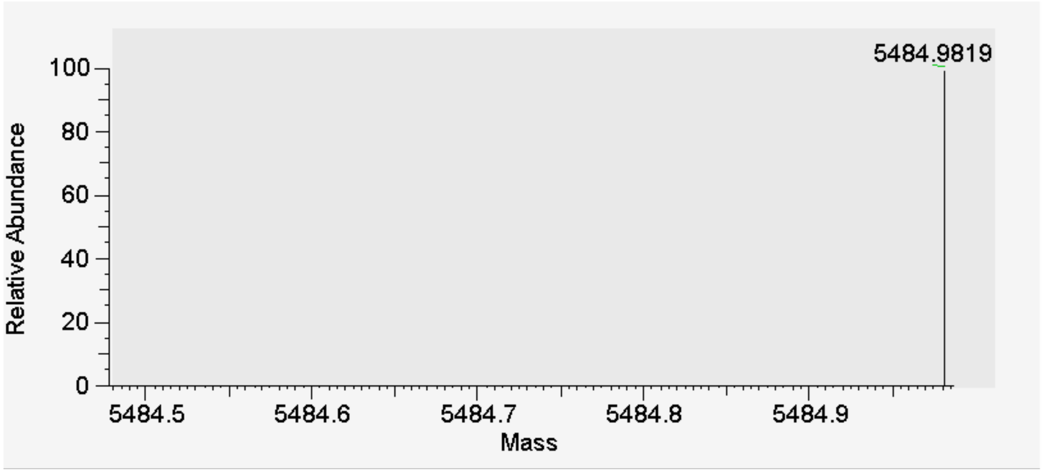

Supporting Figure 39: SAX-HPLC chromatograph of purified 5'-TCA TXA TGA CGC TTA CGA-3' (X = O^4-Et dT)

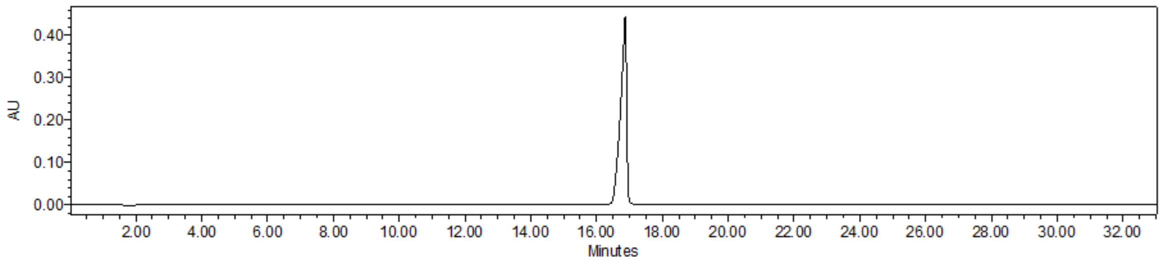

Gradient elution of 0-52.5 % B over 24 minutes. Buffer A: 100 mM Tris HCl, pH 7.5, 10% ACN and Buffer B: 100 mM Tris HCl, pH 7.5, 10% ACN, 1 M NaCl. The column was monitored at 260 nm.

Supporting Figure 40: ESI MS spectrum of purified 5'-TCA TXA TGA CGC TTA CGA-3' (X = O^4-Et dT)

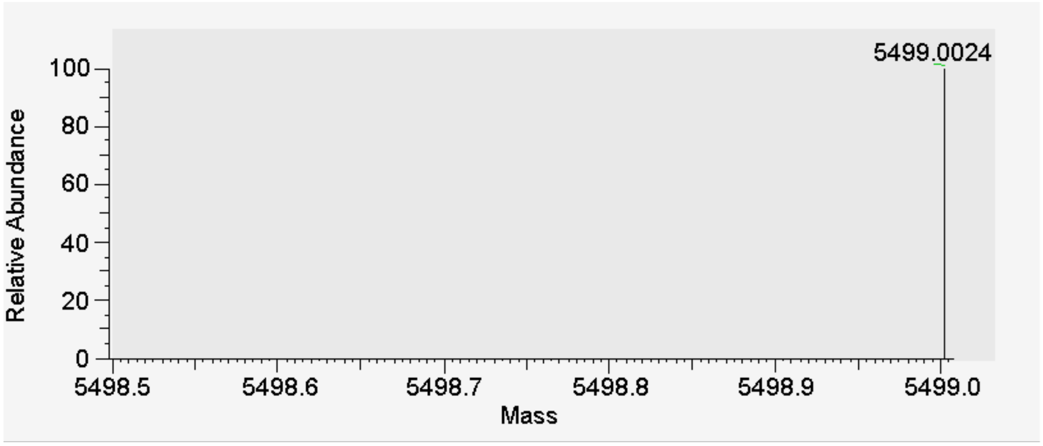

Supporting Figure 41: SAX-HPLC chromatograph of purified 5'-TCA TXA TGA CGC TTA CGA-3' (X = O^4-Me tT)

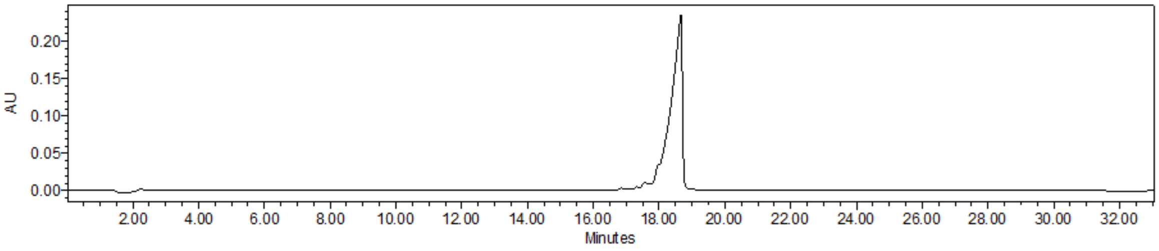

Gradient elution of 0-52.5 % B over 24 minutes. Buffer A: 100 mM Tris HCl, pH 7.5, 10% ACN and Buffer B: 100 mM Tris HCl, pH 7.5, 10% ACN, 1 M NaCl. The column was monitored at 260 nm.

Supporting Figure 42: ESI MS spectrum of purified 5'-TCA TXA TGA CGC TTA CGA-3' (X = O^4-Me tT)

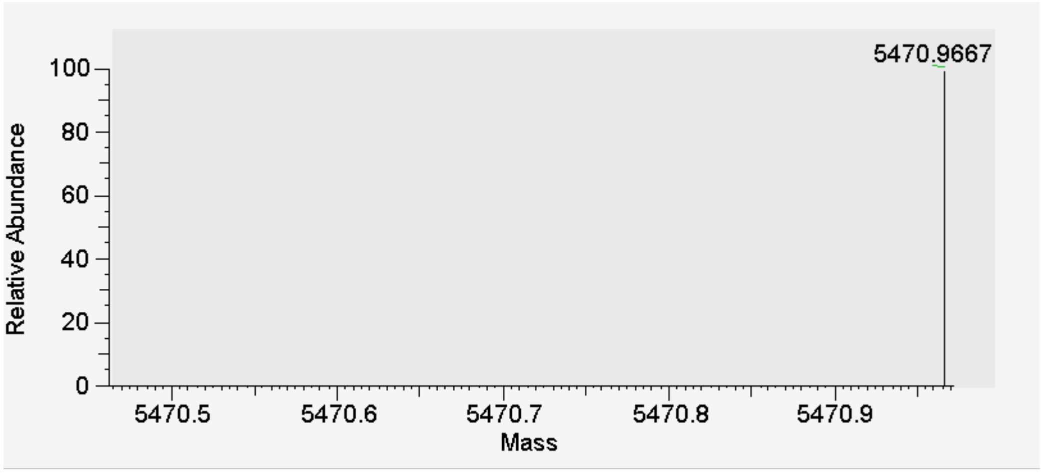

Supporting Figure 43: SAX-HPLC chromatograph of purified 5'-TCA TXA TGA CGC TTA CGA-3' (X = O^4-Et tT)

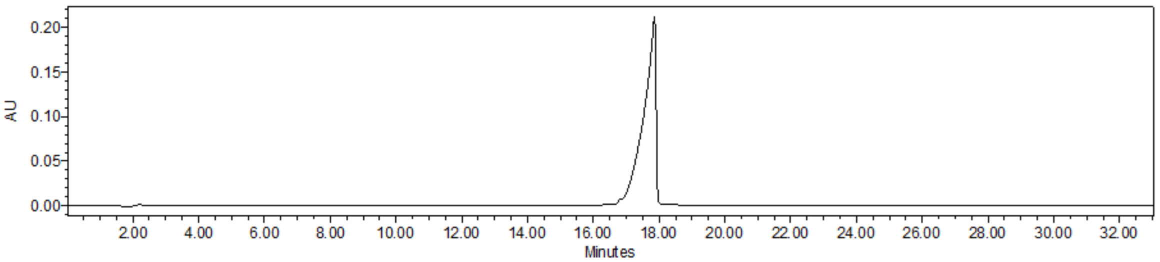

Gradient elution of 0-52.5 % B over 24 minutes. Buffer A: 100 mM Tris HCl, pH 7.5, 10% ACN and Buffer B: 100 mM Tris HCl, pH 7.5, 10% ACN, 1 M NaCl. The column was monitored at 260 nm.

Supporting Figure 44: ESI MS spectrum of purified 5'-TCA TXA TGA CGC TTA CGA-3' (X = O^4-Et tT)

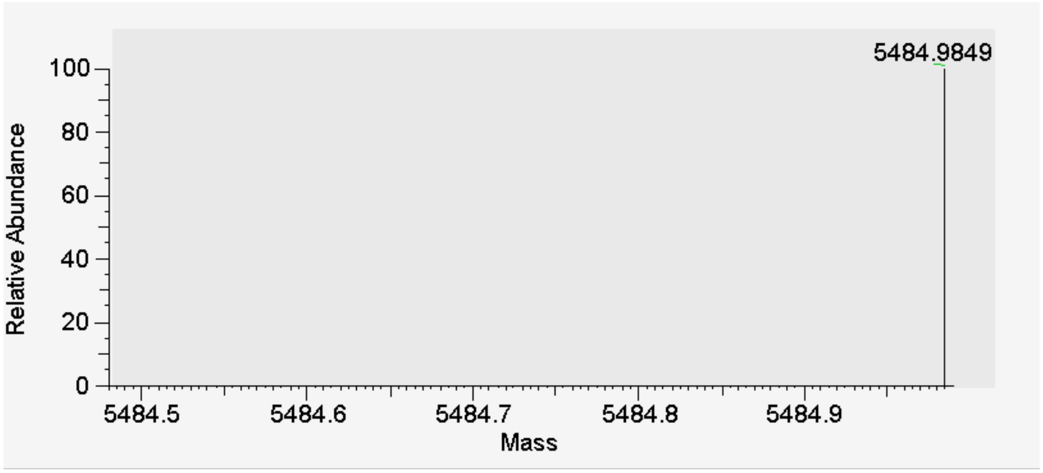

References

1. Sau, S. P.; Fahmi, N. E.; Liao, J.-Yu.; Bala, S.; Chaput, J. C. A scalable synthesis of α-L-threose nucleic acid monomers. *J. Org. Chem.* **2016**, *81*, 2302–2307.
